# Demystifying the link between periodontitis and oral cancer: a systematic review integrating clinical, pre-clinical, and *in vitro* data

**DOI:** 10.1007/s10555-025-10285-z

**Published:** 2025-09-09

**Authors:** Suzane C. Pigossi, Jovânia A. Oliveira, Marcell C. de Medeiros, Lélio F. F. Soares, Nisha J. D’Silva

**Affiliations:** 1 Department of Periodontics and Oral Medicine, University of Michigan School of Dentistry, 1011 North University Ave, Room G018, Ann Arbor, MI 48109-1078 USA; 2 Department of Periodontology and Implantodontology, Federal University of Uberlandia, 1102 República Do Piratini St, Uberlandia, Minas Gerais Brazil; 3 Department of Diagnosis and Surgery, School of Dentistry at Araraquara, Sao Paulo State University - UNESP, 1680 Humaita St, Araraquara, São Paulo, Brazil; 4 Department of Pathology, University of Michigan Medical School, 1500 E Medical Center Dr, Ann Arbor, MI USA; 5 Rogel Cancer Center, University of Michigan, 1500 E Medical Center Dr, Ann Arbor, MI USA

**Keywords:** Head and neck squamous cell carcinoma, Oral squamous cell carcinoma, Periodontal disease, Microbiota, Association or causation

## Abstract

Chronic inflammation and microbial dysbiosis have been implicated in the development of head and neck squamous cell carcinoma (HNSCC), particularly oral cavity squamous cell carcinoma (OSCC). Periodontitis is a common chronic inflammatory disease characterized by the progressive destruction of tooth-supporting structures. While periodontitis Has been associated with an increased risk of OSCC in epidemiological and mechanistic studies, the strength of this association is unclear. This systematic review and meta-analysis aimed to clarify this relationship by synthesizing current evidence, including potential biological mechanisms. A comprehensive search strategy was implemented across five databases to identify relevant publications up to June 2025. Eligible studies had human, *in vitro*, or animal data that evaluated the association between periodontitis and OSCC. A meta-analysis was conducted to assess the association between periodontitis and OSCC. From 6,992 records identified, 116 studies were included in the systematic review, and 26 were eligible for meta-analysis. The meta-analysis supports an association between both clinically assessed and self-reported periodontitis and an increased risk of OSCC, with similar associations observed for HNSCC overall. These clinical associations are supported by experimental evidence demonstrating that key periodontal pathogens, such as *Porphyromonas gingivalis* and *Fusobacterium nucleatum*, contribute to OSCC progression through mechanisms involving inflammation, epithelial-to-mesenchymal transition (EMT), and immune evasion. In conclusion, although epidemiological and mechanistic studies discussed in this review provide evidence of an association between periodontitis and OSCC, future studies using standardized diagnostic criteria, robust design, and clinically relevant experimental models are essential for causal inferences and to deepen knowledge of underlying mechanisms.

**Graphical Abstract:**

Summary graphic of the methodology and analysis of a systematic review on periodontitis and oral cancer, based on cohort, case-control, animal and *in vitro* studies. It highlights the observed associations, with recommendations for standardization of methodology in future studies and a summary of knowledge gaps.

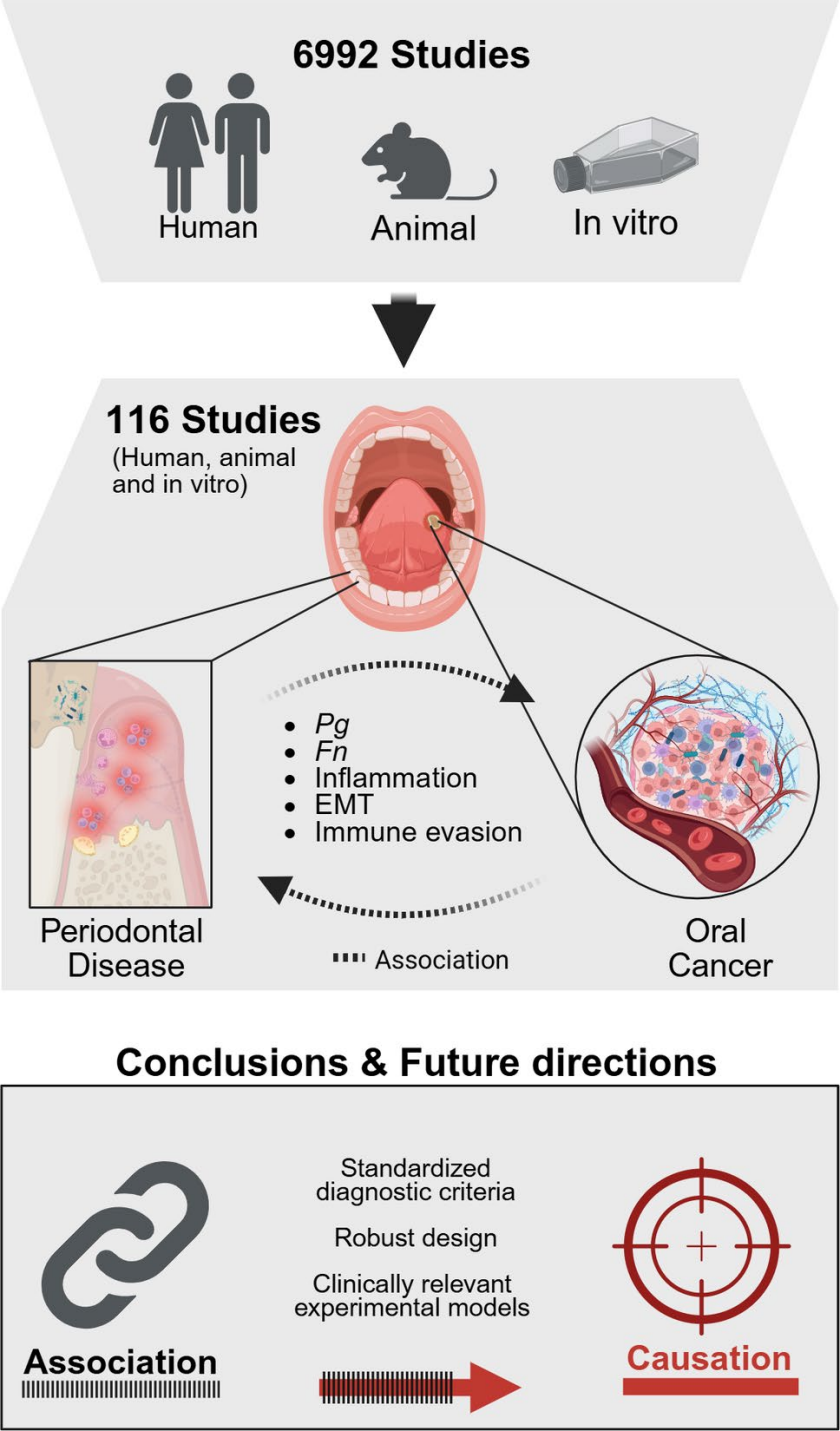

**Supplementary Information:**

The online version contains supplementary material available at 10.1007/s10555-025-10285-z.

## Introduction

Malignancies in the head and neck region are a significant cause of cancer mortality worldwide, with an estimated 758,020 new cases and 379,069 deaths in 2022 [1]. Most malignancies (~ 90%) in this region are squamous cell carcinomas (HNSCC) [2] that arise from mucosal epithelium of the oral cavity, pharynx, and larynx. HNSCC occurs more frequently in men than women, with a male-to-female ratio of ~ 2:1 [3], and predominantly affects individuals over 65 years [4, 5]. Based on Surveillance, Epidemiology, and End Results (SEER) data, 5-year Survival for oral cavity and pharynx cancers combined was 68.9% in 2016 and 69.5% in 2021, across all stages [6].

The oral cavity is the most common subsite for head and neck cancers [7]. Oral cavity squamous cell carcinomas (OSCC) arise from precancerous lesions (epithelial dysplasia, carcinoma-in-situ) and typically present as red or white patches (erythroplakia and leukoplakia, respectively), or exophytic or endophytic lesions with or without non-healing ulcers [2]. These cancers exhibit high recurrence rates, resistance to treatment, and metastasis. In 2022, it was estimated that there would be 389,846 new cases of OSCC and 188,438 OSCC-related deaths, making it the 15th leading cause of cancer-related mortality worldwide [1]. The number of incident cases is projected to rise to 579,096 by 2045, representing a 48.5% increase compared to 2022 [1]. Additionally, based on SEER cancer stat facts, 59,660 new cases of oral cavity and pharynx cancer and 12,770 related deaths are projected to occur in the United States in 2025 [6]. In addition, SEER 5-Year Relative Survival Rates for 2015–2021 demonstrate that Survival for OSCC varies by location, with rates ranging from 90.4% for Lip cancer to 52.4% for floor-of-mouth cancer [6].

Surgical resection with possible adjuvant radiation and chemotherapy, is the main treatment for OSCC, regardless of stage [8]. Adverse effects include speech and swallowing difficulties and may require a tracheostomy or feeding tube [9]. Despite interventions, 5-year Survival rates remain around 70% to 90% for early (localized) stages, varying by location, while overall survival for late-stage disease is ~ 20% to 40% [6]. Therefore, a comprehensive understanding of risk factors is crucial for effective prevention of OSCC [9].

The main risk factors associated with OSCC are tobacco, alcohol, and betel quid [10, 11]. Use of tobacco and alcohol significantly increased the odds of developing OSCC, particularly when smoked and smokeless tobacco, and alcohol were used together (OR = 16.17). Among individual products, unfiltered smoked (OR = 8.38) and smokeless (OR = 6.82) tobacco were strongly associated with OSCC. Alcohol consumption alone posed a substantial risk (OR = 5.01), reinforcing its role as an independent risk factor [12]. Cigarettes contain multiple carcinogens, such as nitrosamines, hydrocarbons, and volatile compounds, that lead to DNA adducts and mutations in genes playing a central role in carcinogenesis [13]. Alcohol is metabolized by alcohol dehydrogenase into acetaldehyde, a compound that reacts with DNA to form adducts, which can trigger mutations or disrupt DNA synthesis, contributing to carcinogenesis [14].

Bacterial infection and chronic inflammation have also been associated with increased risk of OSCC [15, 16]. Pro-inflammatory cytokines and mediators released by inflammatory cells during persistent chronic inflammation could lead to DNA damage, oncogene activation, and inactivation of tumor suppressor genes due to elevated oxidative stress [17]. Moreover, inflammation can promote cancer growth, invasion, and metastasis by stimulating angiogenesis, proliferation, and survival [18]. Inflammation is also linked to suppression of anti-tumor immune responses, allowing the tumor to evade host immune surveillance, which is crucial for tumor progression [19].

Periodontitis is a complex, multifactorial oral inflammatory disease triggered by an exaggerated immune response to dysbiosis in plaque biofilms, resulting in progressive destruction of tooth-supporting structures [20]. Over 1 billion people were affected by severe periodontitis in 2021, with a global age-standardized prevalence rate of 12.5% [21] that is projected to exceed 1.56 billion by 2050, reflecting an annual growth rate of 3.06% [21]. While age-standardized prevalence showed no major sex differences, it was slightly higher in males. Prevalence peaked in individuals aged 50–64 years before declining and stabilizing around 80 years [21]. Clinically, periodontitis manifests as gingival inflammation (erythema, swelling, bleeding on probing), clinical attachment loss, periodontal pocket formation, gingival recession, furcation involvement, and radiographic bone loss [22]. Symptoms include increased tooth mobility, migration, and tilting [22]. The consequence of periodontitis progression is loss of permanent teeth (edentulism), which impacts quality of life, masticatory function/food intake, and psychosocial well-being [23].

Periodontitis is epidemiologically associated with various chronic inflammation-driven conditions, including cardiometabolic diseases [24–26], neurodegenerative disorders [27, 28], gastrointestinal diseases [29] and cancer [30]. Since periodontitis is associated with chronic inflammation, oral pathogens may play a significant role in cancer initiation and progression. In particular, *Fusobacterium nucleatum (F.n.)* is linked to breast cancer, promoting growth and metastasis via lectin-mediated binding to Gal-GalNAc on tumor cells [31], with a modest increased risk of breast cancer reported in women with periodontitis [32]. In pancreatic cancer, *F.n.* is found in 15.5% of patients and associated with large tumors and poor prognosis by enhancing CXCL1 secretion, recruiting myeloid-derived suppressor cells, and suppressing CD8 + T cells [33]. *Porphyromonas gingivalis* (*P.g.*) is linked to pancreatic cancer, detected in both oral and tumor tissues from pancreatic cancer patients, and accelerates tumor development in mice through a neutrophil-dominated pro-inflammatory microenvironment with neutrophil-secreted chemokines and elastase [34, 35]. Periodontitis is also associated with colorectal cancer [36]. *F.n.*, identified in colorectal cancer [37, 38], promotes progression by binding to E-cadherin via FadA adhesin, activating β-catenin signaling, and triggering oncogenic pathways [39]. Additionally, *P.g.* potentiates colorectal cancer by increasing tumor counts and volume, recruiting myeloid cells, and inducing a pro-inflammatory signature [40].

Several epidemiological studies identified a significant association between periodontitis and increased risk of HNSCC [41–43] and OSCC [15, 44, 45]. Moreover, specific periodontal pathogens, including *P.g.* and *F.n.*, were more abundant in OSCC than normal tissue [15, 45]. Experimental studies demonstrate that *P.g.* and *F.n.* suppress epithelial and upregulate mesenchymal markers, indicating that these bacteria can induce epithelial-to-mesenchymal transition (EMT)-like changes in OSCC cells [46–48], a feature that promotes OSCC progression [49]. Additionally, *P.g.*-induced IL-8 production enhances invasion of OSCC via upregulation of MMP secretion [46, 50, 51]. Notably, chronic co-infection with *F.n.* and *P.g.* facilitates OSCC progression *in vivo* [52] and biofilm-induced periodontitis has been associated with large lesions in carcinogen-induced OSCC [53]. Conversely, some microbiota that are negatively correlated with periodontitis, such as *Neisseria sicca* and *Corynebacterium matruchotii*, suppress OSCC development *in vitro* [54].

In contrast, some studies failed to establish a clear association between OSCC and periodontitis [55, 56]. Furthermore, significant variation exists among methods to assess periodontitis. While some studies rely on clinical measurements, such as periodontal clinical parameters [15, 44, 45] and radiographic assessment of alveolar bone loss [42, 57], others used self-reported measures [58–60] contributing to inconsistencies. The goal of this systematic review is to address these gaps by synthesizing data from multiple studies to provide a comprehensive understanding of the potential association between OSCC and periodontitis. Based on literature, we will suggest a mechanism by which the microbiome and host response to periodontitis influence OSCC development and progression. Finally, this review will establish a framework to guide future epidemiological studies in assessing the relationship between periodontitis and OSCC with consistency and precision.

## Materials and methods

### Protocol and registration

The present systematic review with meta-analysis was conducted following the Preferred Reporting Items for Systematic Reviews and Meta-Analyses (PRISMA) statement [61], and the protocol was registered in PROSPERO (ID: CRD42024552444).

### Focused question

The focused question, elaborated by PECO framework (Population, Exposure, Comparator, and Outcome) was: “Does the microbiome and host response associated with periodontitis influence OSCC development/progression?”.

### Eligibility/Inclusion criteria

Original research articles reporting on human, animal, or *in vitro* studies that investigated the association between periodontitis and OSCC were included.

For human studies, eligible study design included case–control and cohort studies involving individuals with clinically or self-reported periodontitis and histologically confirmed OSCC. To be eligible, studies had to include data related to tumors located in the oral cavity. Studies reporting broader HNSCC populations were included only if oral cavity subsites were included, even if not isolated. Phenotypic functional studies that analyzed human tumor tissues or related biological samples to evaluate microbial profiles, immune responses, or molecular markers associated with both periodontitis and OSCC were also included, provided they were accompanied by periodontal data (clinical, radiographic, or self-reported) or were supported by *in vitro* or animal experiments that met the following eligibility criteria.

For *in vitro* studies, eligible studies reported experiments using human/animal OSCC cell lines, epithelial cells, or immune cells exposed to periodontopathogens or their components, which evaluated cancer-relevant mechanisms such as proliferation, apoptosis, invasion, inflammation and EMT.

For animal studies, eligible studies reported models of periodontitis (e.g., ligature-induced or microbial inoculation) in combination with tumor induction by injection of SCC cells or carcinogen (4NQO, 4-Nitroquinoline 1-oxide), and assessed cancer-related outcomes such as tumor growth, immune modulation, or signaling pathways. In studies involving more than one modality (e.g., human and *in vitro*), inclusion was allowed if at least one component met eligibility criteria.

The following were excluded: review articles, conference abstracts, editorials, letters to the editor, book chapters, and studies not published in English. Additionally, studies without assessment of periodontal status (either clinical, radiographic, or self-reported) or evaluation of microbial presence (e.g., in tumor tissue or saliva) without linking periodontal findings, were excluded.

### Literature search

Comprehensive search strategies were implemented across PubMed/MEDLINE, Embase, Web of Science, Scopus, and The Cochrane Library databases to identify relevant publications up to June 16, 2025. These strategies were developed using Medical Subject Headings (MeSH) and Embase Subject Headings (Emtree). Boolean operators (AND, OR, NOT) were employed to combine descriptors and optimize the search, adhering to the syntax rules of each database (Supplementary Table S1). No restrictions were applied regarding publication period.

Search results from electronic databases were managed with the Covidence® platform. Duplicate references were automatically identified and removed within the software. Then results were screened by titles and abstracts using the Covidence® screening tool. References in included articles were reviewed to identify additional relevant studies that were not identified during database searches.

### Data selection and extraction

Two investigators (S.C.P and J.A.O) independently performed the initial search to assess titles and abstracts for relevance. Results were checked for agreement; conflicts were resolved following discussion with a third investigator (M.C.M).

Two investigators (S.C.P and J.A.O) independently read and assessed all full texts of relevant articles based on selection criteria, and extracted data using a standardized form in the Covidence® platform. Data extracted were categorized per study design: For human studies, extracted data included: i) study design; ii) country; iii) number and gender of participants; iv) participants'age (mean and standard deviation); v) criteria for subject selection in case and control groups; vi) assessment methods for periodontitis and cancer; vii) confounding factors; viii) observed relationship between periodontitis and OSCC (positive, negative, or no relationship); ix) odds ratios; x) evaluation methods for periodontal pathogens; xi) tissue evaluation methods; and xii) main outcomes.

For *in vitro* studies, extracted data included: i) cell type; ii) periodontitis-related bacteria used; iii) co-culture protocol; iv) evaluation methods; v) main outcomes; and vi) investigated mechanisms.

For animal studies, extracted data included: i) animal model/type; ii) experimental groups; iii) sample size per group; iv) functional experiments (e.g., gene silencing, transgenic mice, or inhibitor use); v) periodontitis induction model; vi) cancer induction model; vii) euthanasia period; viii) evaluation methods; ix) microbiome data; x) main outcomes; and xi) underlying mechanisms.

### Quality assessment

Two reviewers (S.C.P and J.A.O) separately assessed the quality of included studies. Any disagreement was resolved via discussion with a third reviewer (M.C.M), after which the study was re-evaluated. Methodological quality of case–control and cohort studies was assessed using JBI’s critical appraisal tool [62]. For case–control studies, nine of 10 questions from the original checklist were applied, excluding question 9 since it was not applicable to the studies evaluated (Supplementary Table S2). For cohort studies, seven of 11 questions were used, with questions 6, 8, 9, and 10 excluded because they were not applicable to included studies (Supplementary Table S3). Each item was assessed “yes” (low risk), “no” (high risk), or “unclear” (insufficient information available to determine risk-of-bias). Bias severity was considered high when the study obtained up to 49% of “yes” answers, moderate when it obtained 50%–69%, and low when it reached more than 70% of “yes” scores; these criteria were used in other studies [63–65]. Animal studies were evaluated using SYRCLE's risk-of-bias tool [66] (Supplementary Table S4), which includes 10 questions addressing selection, performance, detection, attrition, reporting, and other biases. Each item was judged as low, high, or unclear risk-of-bias; “yes” indicated low, “no” indicated high, and “unclear” was assigned when available information was insufficient for appropriate assessment. Quality assessment analysis was not conducted for *in vitro* studies due to absence of a universally accepted standard tool for assessing the risk-of-bias.

### Data synthesis: meta-analysis

Meta-analysis was conducted to evaluate the association between periodontitis and OSCC, as well as overall HNSCC. Specifically, case–control studies were included if they either reported sufficient data to calculate cancer risk associated with periodontitis or allowed comparison of periodontal parameters (radiographic bone loss, probing depth, clinical attachment level and bleeding on probing) between cancer patients and controls. Included studies were categorized based on methods used to assess periodontitis; self-reported gingival bleeding (questionnaire), self-reported gum disease (questionnaire), clinical diagnosis of gingival inflammation, clinical diagnosis of periodontitis, and SCC location (HNSCC or OSCC). The odds ratio (OR) with a 95% confidence interval (CI) was calculated to evaluate the relationship between periodontitis and HNSCC/OSCC. Additionally, weighted mean difference (WMD) with a 95% CI was calculated to compare radiographic bone loss, probing depth, clinical attachment loss, and bleeding on probing between case and control groups. To assess heterogeneity among studies, inconsistency statistics (I^2^) were calculated, and significant heterogeneity was present when I^2^ was > 50% [67]. Random-effects analysis, generation of forest plots, and assessment of heterogeneity were performed with the ‘meta’ package version 4.18–0 [68] for RStudio version 4.0.4 [69]. Reporting bias analysis using funnel plots was also performed using Review Manager 5.4 software.

## Results

### Study selection

**Fig. 1:**
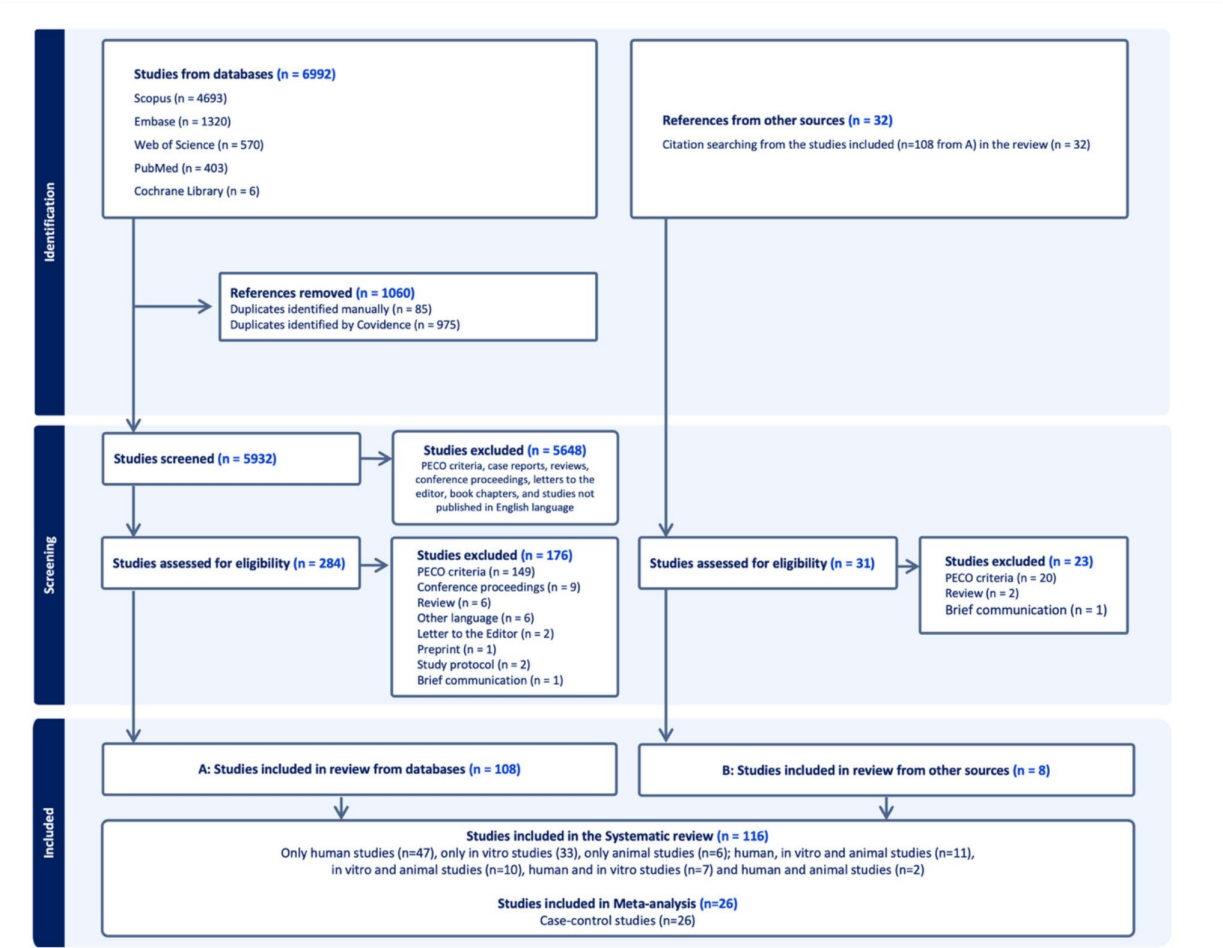
Flowchart of the search strategy of the study. PECO exclusion criteria were defined as studies without exposure (no clinical periodontal data), outcomes (no OSCC risk/severity analysis), and study design (no case–control or cohort study)

A search in online databases found 6,992 results, with 5,932 unique citations. 5,648 were excluded based on title and abstract. 316 publications (284 from database search and 32 from other search methods) were evaluated by full-text reading. Of these, 200 publications (176 from the database search and 24 via other search methods) were subsequently excluded based on inclusion criteria (Supplementary Table S5) and 116 were included in the systematic review (qualitative analysis). Of these 116 publications, 26 were used for the meta-analysis (Fig. 1).

### Characteristics of eligible studies

**Fig. 2:**
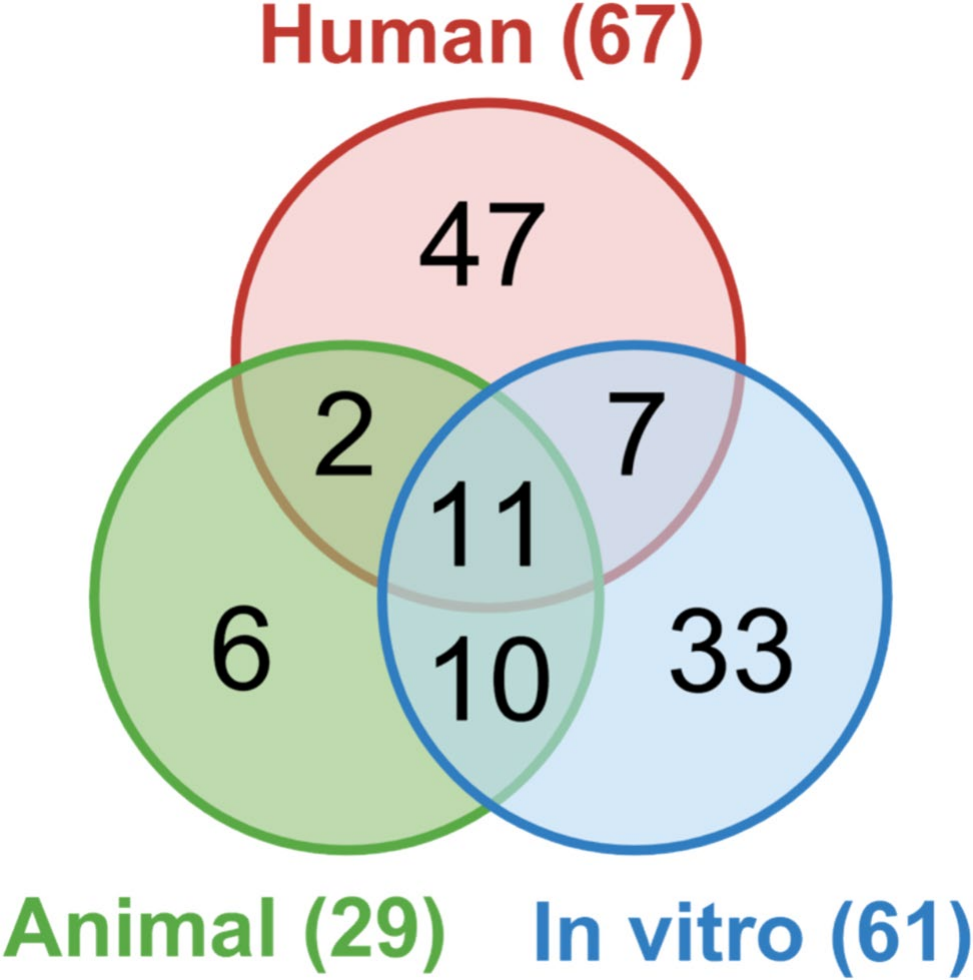
Venn diagram illustrating the distribution and overlap of included studies by category (human, *in vitro*, and animal)

A total of 116 studies were included and categorized as only human (47 studies), only *in vitro* (33 studies), only animal (6 studies), or combinations of these (30 studies) (e.g., 10 studies were *in vitro* and animal). For descriptive purposes, studies were grouped under the categories “human,” “*in vitro*,” and “animal.” A Venn diagram (Fig. 2) illustrates the distribution and overlap between study categories.

Sixty-seven studies included in the systematic review were categorized as human studies (Supplementary Extraction Tables), with main characteristics summarized in Table 1. Human studies were sub-categorized as case–control (thirty-five), cohort (thirteen), and phenotypic functional (nineteen) studies. The thirty-five case–control studies compared individuals with and without OSCC to evaluate periodontitis exposure, and the 13 cohort studies assessed presence of periodontitis in patients with OSCC. Some of these studies also evaluated periodontal pathogens and tumor-related markers in tumor biopsies. Among the case–control studies, 5 evaluated periodontal pathogens, 3 analyzed tumor-related markers in tumor biopsies, and 3 investigated both; one of these also included tongue swab and saliva samples, along with complementary animal experiments. Of the 13 cohort studies, 2 assessed only periodontal pathogens, and 1 evaluated both periodontal pathogens and tumor-related markers. Of the phenotypic functional studies, 2 included only tumor biopsy analyses to evaluate periodontal pathogens, 4 focused on cancer/immunological markers, and 5 investigated both, all in combination with *in vitro* and animal assessments. Moreover, 3 studies combined tumor biopsy evaluations for periodontal pathogens, 3 for cancer/immunological markers, and 1 study evaluated both, all in conjunction with *in vitro* analyses. Finally, one phenotypic functional study examined periodontal pathogens in saliva, dental and tongue plaque, and in tumor biopsies, complemented by animal evaluations.

**Table 1:** Main results of the qualitative analysis (systematic review) for human studies (*n* = 67)

Outcomes	Variables/value	Number of studies
**Study design**	Case–control studies Phenotypic functional studies Cohort studies	35 19 13
**Country**	United States China India Germany Brazil Egypt Japan Norway Turkey Canada Finland Hungary Korea Pakistan Sweden Spain Taiwan United Kingdom	17 16 7 6 4 2 2 2 2 1 1 1 1 1 1 1 1 1
**Cancer population characteristics**		
** Case group**		
Number of individuals	13,731 NA	65 2
Age	< 50 years > 50 years NA NI	16 47 1 21
Gender		
Female Male	3,208 9,152 NA NI	50 50 1 16
** Control group**		
Number of individuals	12,933 NA	51 16
Age^&^	< 50 years > 50 years NA NI	12 31 18 16
Gender		
Female Male	4,146 8,153 NA NI	36 28 16 15
**Periodontitis assessment**	Periodontal clinical examination Radiographic alveolar bone loss Self-reported diagnosis of gum disease Self-reported frequency of gingival bleeding Assessment of gingival inflammation by clinical examination Periodontal disease diagnosis from database Self-reported gingival inflammation/gingival recession NA NI	16 15 8 4 4 2 1 15 2
**Cancer assessment**	Biopsy Others NA NI	40 3 1 23
**Relationship between Periodontitis and OSCC** (only for case–control and cohort studies)	Positive Negative	37 11
**Periodontal pathogens evaluation**		23^*^
Sample	Tissue Saliva Subgingival plaque Mouth swab/smear samples Oral rinse Tongue plaque NI	15 5 4 2 2 1 1
Measurement method	16S rRNA gene sequencing Fluorescence *in situ* hybridization PCR Immunohistochemistry Immunofluorescence Culture and sensitivity test Smear preparation Multicolor immunofluorescence NI	8 6 5 4 3 1 1 1 2
Bacteria type	*Porphyromonas gingivalis* *Fusobacterium nucleatum* *Treponema denticola* *Tanerella forsythia* *Capnocytophaga* *Capnocytophaga gingivalis* *Peptostreptococcus* *Prevotella intermedia* *Streptococcus sanguinis* *Streptococcus gordonii* *Streptococcus mutans* *Streptococcus* NI	13 7 2 2 1 1 1 1 1 1 1 1 4
**Tissue evaluation**		20^#^
Methods of analysis	Immunohistochemistry Immunofluorescence Western blot ELISA Hematoxylin and eosin PCR RNA sequencing Chromatin Immunoprecipitation Colorimetric LDH Assay Fluorescence *in situ* hybridization Flow cytometry *In situ* hybridization	10 7 4 3 3 3 3 1 1 1 1 1
Markers	H3cit MPO CD8 AHR, AKT1, BIM, c-Jun, CD11b, CD4, CD44, CD68, CXCL2, CXCR4, DC-SIGN, DEFA4, Docking protein 3, FOXO1, FOXP3, Gr-1, hBD-1, hBD-2, hBD-3, HNP-1/3, IDO1, IFN-γ, IL-6, IL-8, IL-17, Ki-67, LDH, LTSCCAT, miR-21, P53, Pan-cytokeratin, PD-1, PDCD4, SDF1, SMYD3, STAT3, TDO2, TLR-4, TLR-9	2 2 2 1

^*^Refers to the number of studies that conducted periodontal pathogen evaluation. Some studies assessed more than one sample type, used multiple methods, and investigated more than one bacterial species. ^#^Refers to the number of studies that conducted tissue evaluation. Some studies used multiple methods and investigated more than one marker. ^&^Both categories, were included in some studies, i.e. the study population was individuals across a wider age range. Abbreviations: AHR: Aryl hydrocarbon receptor; AKT1: AKT serine/threonine kinase 1; BIM: BCL-2 interacting mediator of cell death; CD11b: Cluster of Differentiation 11b; CD4: Cluster of Differentiation 4; CD8: Cluster of Differentiation 8; CD44: Cluster of Differentiation 44; c-Jun**:** Jun proto-oncogene; CXCL2: C-X-C motif chemokine Ligand 2; CXCR4: C-X-C motif chemokine receptor 4; DC-SIGN: Dendritic cell-specific intercellular adhesion molecule-3-grabbing non-integrin; DEFA4: Defensin alpha 4; ELISA: enzyme linked immunosorbent assay; FOXO1: Forkhead box O1; FOXP3: Forkhead box P3; Gr-1: Granulocyte differentiation antigen 1; H3cit: Citrullinated histone H; HNP-1/3: Human neutrophil peptide 1/3; hBD-1: Human beta-defensin 1; hBD-2: Human beta-defensin 2; hBD-3: Human beta-defensin 3; IDO1: Indoleamine 2,3-dioxygenase 1; IFN-γ: Interferon gamma; IL: Interleukin; Ki-67: Kiel-67; LDH: Lactate dehydrogenase; LTSCCAT: Laryngeal squamous cell carcinoma-associated transcript; miR-21: MicroRNA 21; MPO: Myeloperoxidase; NA: Not applicable; NI: Not indicated; PCR: polymerase chain reaction; PDCD4: Programmed cell death protein 4; P53: Tumor protein p53; PD-1: Programmed cell death 1; SDF1: Stromal cell-derived factor 1; SMYD3: SET and MYND domain containing 3; STAT3: Signal transducer and activator of transcription 3; TDO2:Tryptophan 2,3-dioxygenase; TLR-4: Toll-like receptor 4; TLR-9: Toll-like receptor 9

Approximately Half of the 67 human studies were conducted in the United States and China (nearly 25% in the USA, 24% in China). The methods used most frequently for assessing periodontitis were periodontal clinical examinations (24% of the studies), radiographic evaluation of alveolar bone loss (22%), and self-reported diagnoses of gum disease (12%). Biopsies were used for cancer assessment in 60% of studies. Considering only case–control and cohort studies, 37 reported a positive association between OSCC and periodontitis, while 11 studies found no association.

Periodontal pathogens in human studies were primarily investigated in tumor biopsies (15 studies), with additional analyses performed on saliva (5 studies), subgingival plaque (4 studies), and other (e.g., mouth swab, oral rinse, tongue plaque) samples (Table 1). The most common methods used to evaluate pathogens were 16S rRNA gene sequencing (35% of studies), fluorescence in-situ-hybridization (26%), and polymerase chain reaction (PCR) (22%). *P.g.* (56% of studies) and *F.n.* (30%) were the most interrogated bacteria. Studies on tumor biopsies, assessed markers involved in immune regulation (CD4, CD8, CXCL2, CXCR4, FOXP3, IFN-γ, IL-6, IL-8, IL-17, PD-1, TLR-4, TLR-9), cell signaling (AHR, AKT1, c-Jun, FOXO1, PDCD4, SMYD3, STAT3), inflammatory and antimicrobial processes (CD68, DC-SIGN, DEFA4, H3cit, hBD-1, hBD-2, hBD-3, HNP-1/3, LDH, MPO), cell proliferation and apoptosis (BIM, CD44, Ki-67, P53), invasion and angiogenesis (IDO1, LTSCCAT, miR-21, SDF1, TDO2). Immunohistochemistry (50%) and immunofluorescence (35%) were the predominant techniques for these studies.

Sixty-one *in vitro* studies were included in the systematic review (Supplementary Extraction Tables). Of these, 33 were exclusively *in vitro*, 11 included human, *in vitro*, and animal data, 7 combined human and *in vitro* data, and 10 included animal and *in vitro* data (Fig. 2). Characteristics of these studies are summarized in Table 2. Thirty-eight *in vitro* studies used cell lines derived from human tongue SCC, including SCC25 (13 studies), CAL27 (13 studies) and HSC-3 (12 studies). *P.g.* was used in 49, and *F.n.* in 20 studies to stimulate cells. Other bacteria included *Escherichia coli* (7 studies), *Streptococcus gordonii* (4 studies) and *Treponema denticola* (*T.d*.) (4 studies), with some studies using multiple bacteria. Genes and proteins evaluated *in vitro* were associated with extracellular matrix degradation, tissue remodeling, and tumor invasion (CYP1A1, FN1, LAMC2, MMP-1, MMP-2, MMP-7, MMP-9, MMP-10); EMT, and cell migration/invasion (E-cadherin, N-cadherin, SNAI1, SLUG, Twist, vimentin, ZEB1); inflammation, tumor progression, and immune evasion (H3cit, IFN-γ, IL-1β, IL-6, IL-8, IL-17, MPO, PD-L1, PD-L2, TNF-α); regulation of signaling pathways, including inflammation, cell cycle, apoptosis, oncogenesis (Akt2, JAK1, MyD88, NF-κB, p21, p53, STAT3, TLR4, TLR2); cell proliferation, signaling, and cancer progression (β-catenin, Cyclin D1, Cytokeratin 13, Ets1, GSK3β, HSP27, Jun, Ki-67). Stemness markers linked to tumorigenesis and metastasis (CD133, CD44), as well as autophagy- and angiogenesis-related markers (LC3, VEGFA, VEGFC), were also analyzed. Methods employed to evaluate these markers and cellular behaviors, included western blot (38 studies), quantitative reverse transcription polymerase chain reaction (RT-qPCR) (36 studies), migration and invasion assays (32 studies), and other techniques (e.g., cell proliferation, immunofluorescence, RNA sequencing, enzyme-linked immunosorbent assay [ELISA], etc.). For normalization, housekeeping genes and structural proteins, usually GAPDH, β-actin, α-tubulin, and tubulin were used.

**Table 2:** Main results of the qualitative analysis (systematic review) for *in vitro* studies (*n *= 61)

Outcomes	Variables/value	Number of studies
**Cell type**	SCC25	13
	CAL27	13
	HSC-3	12
	Ca9-22, SAS, SCC-9	5
	PHGK, SCC-7	4
	HEK 293 T, HIGK, HSC-4, OSC-20, TIGKS, THP-1, HN12, HN6	3
	BHY, H400, HIOEC, Tca8113, HN4, HN30	2
	Other types (total of 41 cell types)	1
**Bacteria**	*Porphyromonas gingivalis*	49
	*Fusobacterium nucleatum*	20
	*Escherichia coli*	7
	*Streptococcus gordonii*	4
	*Treponema denticola*	4
	*Streptococcus mutans*	3
	*Neisseria sicca*	2
	*Streptococcus salivarius*	2
	Other types (total of 9 bacteria)	1
**Methods of evaluation**	Western blot	38
	RT-qPCR	36
	Migration and invasion assay	32
	Cell proliferation assay	23
	Cell Immunofluorescence assay	17
	RNA- sequencing	13
	ELISA	12
	Cell viability assay	12
	Gelatin zymography	10
	Scratch wound assay	10
	Flow cytometry (markers analysis)	9
	Flow cytometry (cell cycle analysis)	8
	RT-PCR	8
	Immunocytochemistry analysis	7
	Apoptosis Analysis	6
	Colony Formation Assay	5
	Multiplex bead assay	3
	Microarray	3
	Cell Transmission Electron Microscopy	2
	ChIP assay	2
	Tumorsphere assay	2
	Other (each study used a different method not mentioned above)	23
**Markers**		
Genes		
	GAPDH	24
	MMP9, Vimentin	8
	IL6	7
	IL8, SNAIL	6
	β-actin, Zeb1	5
	Ecadherin, MMP1, N-cadherin, TGFβ1	4
	Cyclin D1, PDL1, Zeb2	3
	CCL5, CYP1A1, CXCL2, FN1, LAMC2, ITGA5, PDL2, I**κ**Bα, MIP- 3α, MMP10, MMP2, NLRP3, PAR2, PAR4, Podoplanin, SLUG, TNFα, TSG6, Twist	2
	Other (176 genes were evaluated only once, each in one included study)	1
	β-actin	14
Protein	MMP-9, E-cadherin, GAPDH	12
	Vimentin	10
	MMP-2	9
	SNAI1	8
	PDL1	6
	IL-8, N-cadherin, NF-kB	5
	Cyclin D1, TNF-α	4
	Actin, CXCL-2, Cytokeratin 13, IL-6, Ki-67, MMP-1, MMP-7, MMP-10, MyD88, PD-L2, SLUG, STAT3, TLR4	3
	α-tubulin, Akt2, CD133, CD44, CXCL-2, Ets1, GSK3β, H3cit, HSP27, IL-1β, IL-17, IFN-γ, JAK1, Jun, LC3, MPO, p21, p53, TLR2, Tubulin, Twist, VEGFA, VEGFC, ZEB1, β-catenin	2
	Other (93 proteins were evaluated only once, each in one included study)	1

Akt2: Serine/Threonine Kinase 2; BHY: buccal mucosa SCC; CAL27: human tongue SCC; Ca9-22: human gingival SCC; CCL5: C–C Motif Chemokine Ligand 5; ChIP assay: Chromatin Immunoprecipitation; CXCL-2: CXC Motif Chemokine Ligand 2; CYP1A1: Cytochrome P450 family 1 Subfamily A member 1; ELISA: enzyme linked immunosorbent assay; Ets-1: Ets translocase 1; FN1: Fibronectin 1; GAPDH: Glyceraldehyde-3-Phosphate Dehydrogenase; GSK3β: Glycogen Synthase Kinase 3 beta; H3cit: Citrullinated Histone H3; H400: human alveolar process SCC; HIGK: Human immortalized gingival keratinocytes; HIOEC: human immortalized oral epithelial cells; HEK293: immortalized human embryonic kidney cells; HN12: tongue SCC; HN6: tongue SCC; HN4: laryngeal SCC, HN30: pharyngeal SCC; HSC-3: human tongue SCC; HSC-4: human tongue SCC; HSP27: Heat Shock Protein 27; IκBα: Inhibitor of kappa B alpha; IFN-γ: Interferon gamma; IL-1β: Interleukin-1 beta; IL-6: Interleukin 6; IL-8: Interleukin 8; IL-17: Interleukin-17; ITGA5: Integrin alpha 5; JAK1: Janus kinase 1; Jun: Transcription factor Jun; LAMC2: Laminin Subunit gamma 2; LC3: Microtubule-Associated Proteins 1A/1B Light Chain 3; MMP: Matrix metalloproteinase; MIP-3α: Macrophage inflammatory protein 3 alpha; MPO: Myeloperoxidase; MyD88: Myeloid Differentiation Primary Response 88; NLRP3: NOD-like receptor protein 3; NF-kB: Nuclear Factor kappa B; OSC-20: human tongue SCC; PAR2: Platelet activation receptor 2; PAR4: Platelet activation receptor 4; PD-L1: Programmed Death-Ligand 1; PD-L2: Programmed Death-Ligand 2; PHGK: human gingival keratinocytes; RT-PCR: reverse transcription polymerase chain reaction; RT-qPCR: quantitative reverse transcription polymerase chain reaction; SCC: squamous cell carcinoma cell line; SCC-25: human tongue SCC; SCC-7: mouse SCC; SCC-9: human tongue SCC; SAS: human tongue SCC; SLUG: Snail family transcriptional repressor 2; SNAIL1: Snail family transcriptional repressor 1; STAT3: Signal transducer and activator of transcription 3; THP-1: human monocyte leukaemia cell line; Tca8113: buccal mucosa SCC; TGF-β1: Transforming Growth Factor Beta 1; TIGKS: telomerase immortalized gingival keratinocytes; TLR2: Toll-Like Receptor 2; TLR4: Toll-Like Receptor 4; TNF-α: Tumor Necrosis Factor-alpha; TSG-6: Tumor necrosis factor-stimulated gene 6; Twist: Twist family BHLH transcription factor 1; VEGFA: Vascular endothelial growth factor A; VEGFC: Vascular endothelial growth factor C; Zeb1: Zinc finger E-box-binding homeobox 1; Zeb 2: Zinc finger E-box-binding homeobox 2

Twenty-nine animal studies were included in the systematic review (Supplementary Extraction Tables); 11 integrated human, *in vitro*, and animal approaches, 10 *in vitro* and animal experiments, 6 were animal only, and 2 involved both human and animal approaches (Fig. 2). Characteristics of these studies are summarized in Table 3. C57BL/6, BALB/c, and BALB/c nude mice, were most frequently used. Periodontitis was primarily induced by oral administration of *P.g.*, either alone or in combination with *F.n.*. Cancer induction was usually by injection of SCC7 (mouse SCC cell line derived from the abdominal wall [70]) (10 studies) or 4NQO carcinogen (7 studies). CAL27 and OSC-20 cells were also used. Various injection sites were used in animal models, including subcutaneous in the dorsal region, such as back (2 mentions), dorsal skin (2), and flanks (2) (some studies administered injections bilaterally on the back). In addition, cells were injected submucosally in the mouse oral cavity, i.e. buccal mucosa (6) and floor-of-mouth (1). Other sites included the right footpad (1), axilla (1) and tail vein (1), reflecting the diverse strategies employed to model tumor initiation and progression.

**Table 3:** Main results of the qualitative analysis (systematic review) for animal studies (n = 29)

Outcomes	Variables/value	Number of studies
Species^*^	C57BL/6 mice BALB/c mice BALB/c mice nude C3H/He mice NOD-SCID mice Wistar rat BALB/c SPF mice NCr nude mice NI	10 6 6 2 2 2 1 1 1
**Functional experiments**	TCR γδ monoclonal antibody PD-1mAb inhibitor CXCL2/CXCR2 inhibitor AHR inhibitor (CH-223191) Clodronate liposomes DNase I DiO labelling Fer-1 Uptake inhibitor (LatA) Mob1a^flox/flox^ Mob1b^−/−^ NF-κB inhibitor (BAY 11–7082) Rosa26^CreERT^ TDO2 inhibitor	2 2 1 1 1 1 1 1 1 1 1 1 1 1
**Periodontitis induction model**	*P.g.* orally administered *P.g.* and *F.n.* orally administered Ligature + microbiota inoculation Pg intraperitoneal injection Pg injection in the tumor Ligature Other (each study used a different method not mentioned above) NA	5 3 2 2 2 2 12 1
**Cancer induction model**	SCC7 cells injection 4NQO carcinogen solution CAL27 cells injection OSC-20 cells injection Other (each study used a different method not mentioned above) NA	10 7 4 2 4 2
**Time to endpoint after bacteria and/or cancer induction**		
** Minimal**	1 h	1
** Maximum**	27 weeks	1
Most frequent^*^	4 weeks 18 weeks 1 day (or 24 h) 22 weeks 8 weeks 21 days 20 days Other (each study used a different endpoint not mentioned above)	4 3 3 2 2 2 2 17
**Methods of evaluation**	Histopathological analysis Immunohistochemistry analysis Immunofluorescence Tumor volume RT-qPCR Western blot µCT (alveolar bone loss) Flow cytometry 16S rRNA sequencing analysis ELISA Morphometrical analysis (using methylene blue (1%)) Multiplex assay Other (each study used a different method not mentioned above)	19 16 12 11 9 5 4 4 3 3 3 2 9
**Markers**		
Genes	IL6 GAPDH, IL1β IL18 ASC, Caspase-1, Foxo3, NFκΒ, NLRP3, TNF-α Other (26 genes were evaluated only once, each in one included study)	6 4 3 2 1
Protein	Ki-67 CD4, CD8 CD11b, CD206, CD3, Cyclin D1, F4/80, γ-H2AX, CD45, IFN-γ, IL6 ATR, CHK1, Foxp3, IL-17, PD-1, RAD51, STAT, TNF-α Other (49 proteins were evaluated only once, each in one included study)	10 4 3 2 1

*Some studies used more than one species, methods of evaluation and included multiple endpoints for euthanasia. Abbreviations: 4NQO: 4-Nitroquinoline 1-oxide; AHR: Aryl hydrocarbon receptor; ASC: Apoptosis-associated speck-like protein containing a CARD; ATR: Ataxia telangiectasia and Rad3 related; CAL27: human tongue squamous cell carcinoma; Caspase-1: Cysteine-aspartic acid protease 1; CD11b: Cluster of differentiation 11b; CD3: Cluster of differentiation 3; CD4: Cluster of differentiation 4; CD8: Cluster of differentiation 8; CD45: Cluster of differentiation 45; CD206: Cluster of differentiation 206; CHK1: Checkpoint kinase 1; CXCL2: CXC Motif Chemokine Ligand 2; DiO: 3,3'-Dioctadecyloxacarbocyanine Perchlorate; ELISA: enzyme linked immunosorbent assay; Fer-1: Ferrostain-1; Fn: *Fusobacterium nucleatum*; Foxo3: Forkhead box O3; Foxp3: Forkhead box P3; GAPDH: Glyceraldehyde-3-phosphate dehydrogenase; IL-1β: Interleukin 1 beta; IL-6: Interleukin 6; IL-17: Interleukin 17; IL-18: Interleukin 18; IFN-γ: Interferon gamma; Ki-67: Kiel-67; LatA: Latrunculin A; Mob1: Mob kinase activator 1 A; Mob1b: Mob kinase activator 1B; NA: Not applied; NI: Not indicated; NFκB: Nuclear factor kappa-light-chain-enhancer of activated B cells; NLRP3: NOD-,LRR- and pyrin domain-containing protein 3; OSC-20: human tongue squamous cell carcinoma; PD-1: Programmed cell death 1; PD-1mAb: Programmed Cell Death Protein 1 monoclonal antibody; Pg: *Porphyromonas gingivalis*; RAD51: Radiation-sensitive 51; RT-qPCR: quantitative reverse transcription polymerase chain reaction; SCC7: Mouse squamous cell carcinoma; SPF: Specific pathogen free; STAT: Signal transducer and activator of transcription; TCR γδ: T-Cell Receptor gamma delta; TDO2: Tryptophan 2,3-Dioxygenase; TNF-α: Tumor Necrosis Factor-alpha; µCT: micro-computed tomography; γ-H2AX: Gamma H2A histone family member X

Genes and proteins evaluated in animal studies were associated with inflammation and immune response (Foxp3, IL1β, IL18, IL6, IL-17, NF-κB, NLRP3, TNF-α); regulation of the inflammasome and apoptosis (ASC, Caspase-1, Foxo3, NLRP3); immune cell activity and macrophage polarization (CD11b, CD206, CD3, CD4, CD45, CD8, F4/80, PD-1); cell proliferation and DNA damage response (ATR, CHK1, Cyclin D1, γ-H2AX, Ki-67, RAD51); and signaling pathways involved in inflammation, genomic stability, and cancer progression (IL6, STAT). For normalization, the housekeeping gene GAPDH was most frequently used. Methods in animal studies to assess tissue changes and quantify protein and gene expression levels included histopathological analysis, immunocytochemistry, immunofluorescence, RT-qPCR, etc.

### Risk of bias of eligible studies

**Fig. 3:**
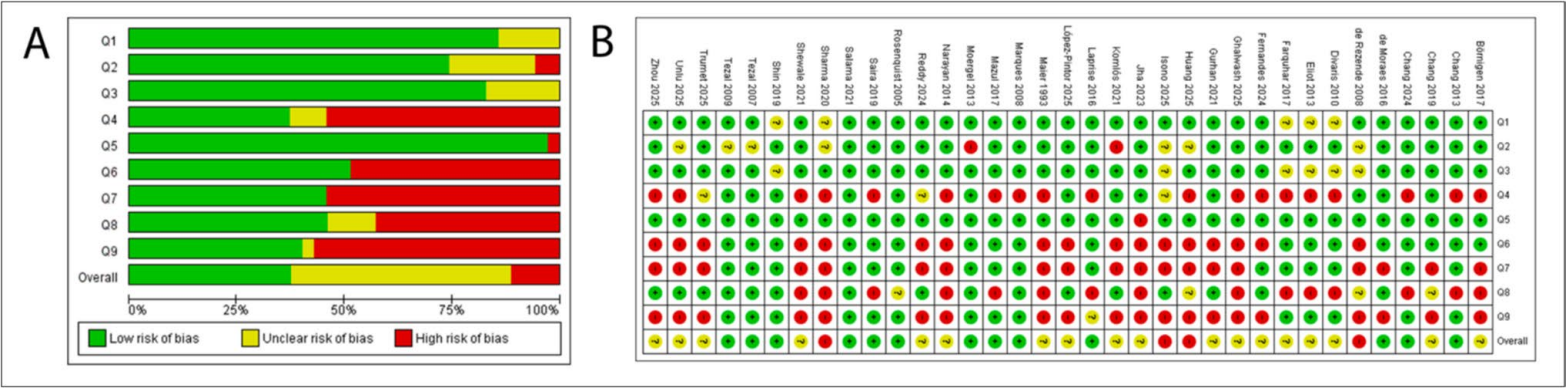
Risk of bias assessed using the JBI critical appraisal tool for case–control studies. (**a**) Summary of the individual study’s authors'judgment on each checklist item, presented as percentages across all included studies. (**b**) Detailed review of the authors'judgments on each checklist item for each included study

For case–control studies, 18 were classified as Having moderate, 13 as low and 4 as high risk-of-bias (Fig. 3). Primary sources of bias included measurement of periodontitis (exposure), infrequently performed with standardized and reliable methods. Other significant issues were absence of confounding factor identification or strategies to address them, inconsistent or unreliable assessment of outcomes (cancer risk/severity) for both cases and controls, and inappropriate statistical analyses.

**Fig. 4:**
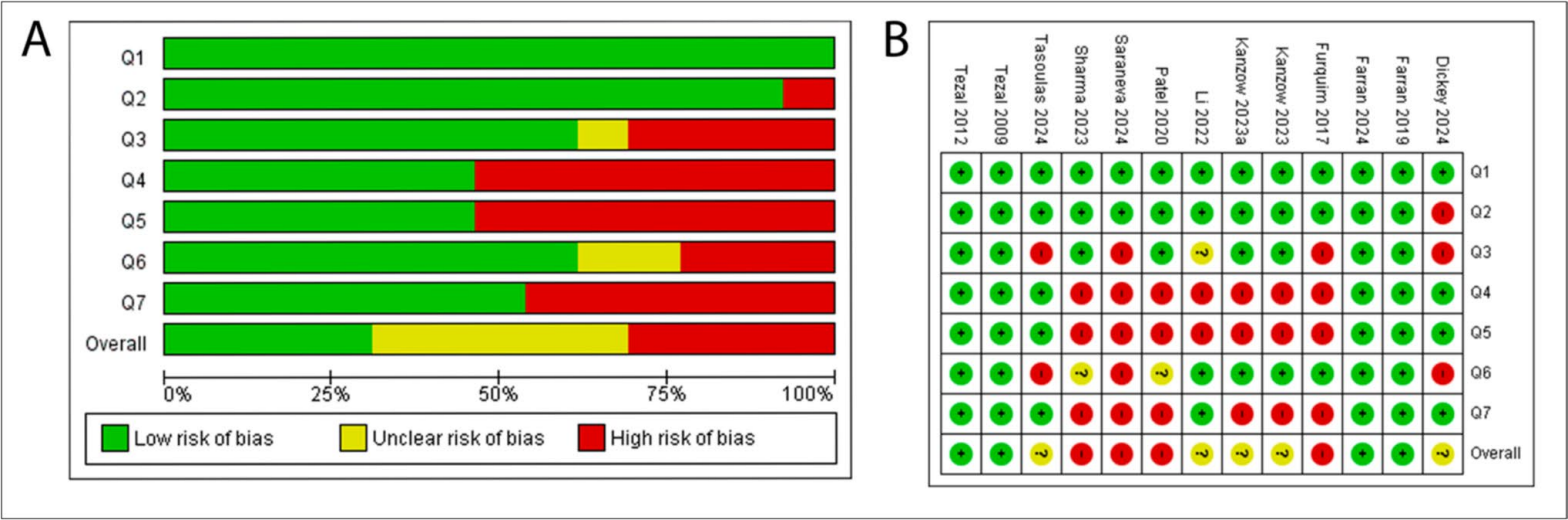
Risk of bias assessed using the JBI critical appraisal tool for cohort studies. (**a**) Summary of the individual study’s authors'judgment on each checklist item, presented as percentages across all included studies. (**b**) Detailed review of the authors'judgments on each checklist item for each included study

For cohort studies, 5 were assessed as Having moderate, 4 as low, and 4 as high risk-of-bias (Fig. 4); main sources of bias were lack of confounding factor identification or methods to mitigate their effects, and inconsistent or unreliable assessment of outcomes (cancer risk/severity).

**Fig. 5:**
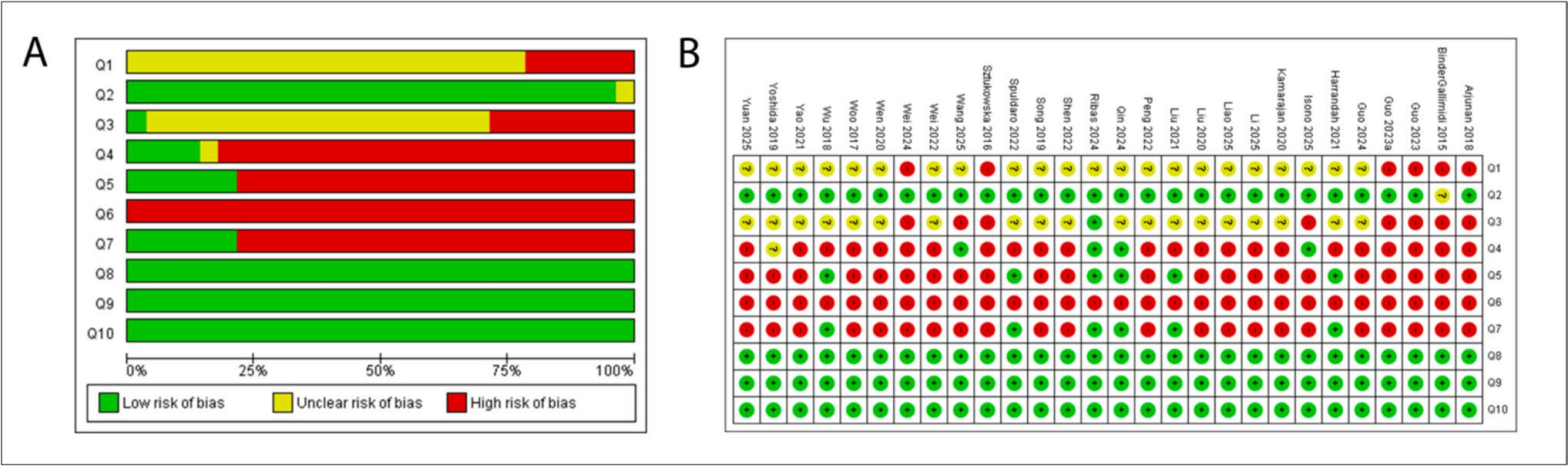
Risk of bias assessed using the SYRCLE risk of bias tool for animal studies. (**a**) Summary of the individual study’s authors'judgment on each domain, presented as percentages across all included studies. (**b**) Detailed review of the authors'judgments on each domain for each included study

For animal studies, primary sources of bias were lack of randomization, intervention blinding, and outcome assessments (Fig. 5).

### Results of individual studies included in the systematic review

#### Human studies associating periodontitis and OSCC

Several studies have highlighted the role of periodontitis-related microbial and immunological factors in OSCC progression and prognosis. Here, we summarize underlying mechanisms reported in human studies that evaluated these factors in tumor biopsies. The relative abundance of *F.n.* and *P.g.* in cancer tissues was positively correlated with subgingival plaque levels [15]. Increased expression of TLR-4/9 mRNA/protein expression in cancer tissue, presence of periodontal pathogens [*F.n.*, *P.g.*, *T.d.*] in mouth smear samples, and gingival inflammation/recession were associated with OSCC incidence [71]. *P.g.* was implicated in OSCC recurrence by upregulating DOK3 protein in tumor-associated macrophages, potentially activating TNF and MAPK pathways [72]. Immunological changes, such as increased CD4 + and CD8 + T cells, were observed during transition from normal to precancer (epithelial dysplasia) and OSCC, correlating with p53 immunostaining [73]. Genes associated with immunosuppression (e.g., *IDO1*, *IL-10*, *CD274*, *ARG1*, and *CD80*) and oncogenesis (e.g., *CXCR4*, *SDF-1*, *AKT1*, and *STAT3*) were significantly upregulated in *ex vivo* isolated blood myeloid and plasmacytoid dendritic cells of patients with chronic periodontitis, while *SOCS3* and *SOCS1* were downregulated [74]. Moreover, the transcript for *IDO1* was upregulated fivefold relative to controls [74]. Increased pAKT1, pFOXO1, and FOXP3 along with decreased BIM proteins, were observed in gingival tissues from patients with chronic periodontitis compared with healthy controls [74]. Downregulation of tumor suppressor genes including IRF1, LDOC1, and FOXO3 was also evident in tissues from patients with chronic periodontitis [74]. Higher transcript of *Mfa1* than *FimA*, was reported in blood dendritic cells [74]. High expression of neutrophil extracellular trap–related markers (H3cit and MPO) in advanced-stage OSCC tissues and serum were associated with clinical indicators such as T stage, N stage, clinical stage, and patient survival [75], supporting previous findings that *P.g.* and neutrophil extracellular traps were elevated in advanced compared to early-stage OSCC, with a positive correlation between MPO-DNA complexes and *P.g* levels [45]. Strong expression of *P.g.*, CXCL2, and tumor-associated neutrophils was observed in advanced stages (III–IV) of OSCC, while weaker expression was found in early stages (I–II). These markers were correlated with clinical indicators and identified as independent prognostic factors for OSCC [76]. In OSCC tissues specimens from patients with periodontitis, higher expression of lipopolysaccharide (LPS)-induced TSCC-associated transcript (LTSCCAT) and SMYD3 was associated with progression, lymph node metastasis, and decreased overall survival [77]. Similarly, OSCC-with-periodontitis samples showed higher proportions of IL-17 + γδ T cells and STAT3 phosphorylation [78]. In addition, OSCC samples from patients with periodontitis exhibited a reduced proportion of IFN-γ⁺ CD8⁺ T cells and significantly elevated PD-L1 expression compared to those without periodontitis [79]. A positive correlation was observed between tumor grade and salivary levels of sCD44, IL-6, and IL-8, with the strongest association seen for sCD44, followed by IL-6 and IL-8 [80]. Colonization by *P.g.* was significantly associated with shorter overall survival in OSCC [81], while *P.g.* DNA levels correlated positively with miR-21 and c-Jun expression and negatively with PDCD4 expression in OSCC tissue [82]. Tryptophan metabolism plays a key role in modulating immune response and cancer immunotherapy, with TDO2 and AHR among its crucial mediators [83]. In HNSCC tissue, TDO2 expression positively correlated with AHR and *F.n.* abundance, and high levels of both markers were associated with poorer overall survival. Moreover, elevated TDO2 and *F.n.* expression were observed in PD-1 mAb non-responders, while lower levels of these markers were linked to longer progression-free survival.

Human studies consistently associate periodontitis with OSCC progression, highlighting the role of *P.g.* and *F.n.* in tumor microenvironment alterations, immune modulation, and poor prognosis. Upregulation of oncogenic pathways, immunosuppressive markers, and inflammatory responses in OSCC tissues from patients with concurrent periodontitis, suggest a strong mechanistic link; functional/mechanistic studies are needed to establish causation.

#### Role of periodontopathogens in EMT, cell proliferation and invasion (*in vitro* studies)

During EMT, epithelial cells lose cell–cell junctions and polarity, acquiring a mesenchymal phenotype that enhances motility and invasiveness, playing a central role in OSCC progression [49, 84]. EMT involves cytoskeletal reorganization, and changes in signaling that enhance movement. Key markers include loss of E-cadherin, claudins, and occludins, and increased N-cadherin, vimentin, fibronectin, and integrins, which remodel cell-extracellular matrix interactions [85]. EMT is controlled by transcription factors including SNAIL, ZEB, and TWIST, while MMPs degrade extracellular matrix to enable invasion [85]. While *P.g.* and *F.n.* induce EMT in various cancer cell models, EMT appears to be dependent on bacterial species, strain variations, and specific molecular interactions [48]. Various studies included in this review demonstrated that *P.g.* and *F.n.* upregulate mRNA expression of key EMT transcription factors, such as *Snail*, *Slug, Twist1*, and *Zeb1*, in OSCC cells [46, 47, 77, 86], primary oral keratinocytes [87], and telomerase immortalized keratinocytes [88], while promoting expression of mesenchymal markers (Vimentin, N-cadherin, MMPs) and suppressing E-cadherin. Additionally, *P.g.* processes β-catenin via gingipains (RgpA/B > Kgp), leading to its nuclear translocation and β-catenin-mediated activation of TCF/LEF transcription in immortalized keratinocytes. This effect was independent of GSK3β signaling [89], but involves degradation of Axin1 and APC, key components of the β-catenin destruction complex. Mutants lacking RgpA/B showed reduced β-catenin cleavage, while FimA loss had no impact [89]. In contrast, Lee et al. (2017), showed that *P.g.*-induced EMT in primary oral keratinocytes involves inhibition of GSK3β, contributing to loss of E-cadherin and nucleo-cytoplasmic accumulation of β-catenin, further promoting mesenchymal traits.

Beyond these mechanisms, *P.g.* drives ZEB1 upregulation, contributing to partial EMT in gingival keratinocytes. This effect requires direct bacterial contact and is mediated by FimA; mutants lacking RgpA/B do not induce ZEB1 expression. ZEB1 deficiency prevents *P.g.*-induced vimentin and MMP-9 upregulation, reducing tumor cell migration, indicating its crucial role in EMT [88]. In addition, outer membrane vesicles of *P.g*. promote EMT in human OSCC cell lines by modulating ferroptosis through the NF-κB signaling pathway. Ferroptosis, an iron-dependent form of cell death driven by lipid peroxidation and anti-oxidant system failure, has been implicated in tumor progression via regulation of EMT, angiogenesis, invasion, and metastasis [90]. Similarly, *F.n.* modulates EMT via MIR4435-2HG, a long non-coding RNA that downregulates miR-296-5p, thereby activating Akt2 and SNAI1 in immortalized oral epithelial and OSCC cell lines. This activation leads to suppression of E-cadherin and upregulation of vimentin and N-cadherin, ultimately increasing cell migration and invasion [91]. Notably, *F.n.*-induces EMT independent of bacterial viability, with heat-inactivated *F.n.* capable of inducing similar molecular changes [91]. Extracellular metabolites from periodontopathic bacteria, such as sodium butyrate, promote EMT by inducing mRNA/protein expression of podoplanin, vimentin, Slug, N-cadherin, and Snail1, while altering E-cadherin localization from membrane to cytoplasm, reinforcing EMT-like changes in OSCC cell lines [92, 93].

OSCC progression is driven by an altered tumor microenvironment, where immune suppression, stromal changes, and hypoxia promote metastasis [7]. Tregs, tumor-associated macrophages, and myeloid-derived suppressor cells inhibit T-cell activity via PD-1/PD-L1 and CTLA-4/CD80/86 pathways, while OSCC cells release VEGF, TGFβ, IL-6, and IL-10 to suppress immunity [7]. Cancer-associated fibroblasts and exosomal miRNAs support tumor growth, with dominance of M2 macrophages enhancing carcinogenesis [7]. In this context, *P.g.* and *F.n.* contribute to tumor growth and immune evasion by modulating inflammatory pathways, enhancing proliferative signals, and disrupting immune surveillance mechanisms. *P.g.* and *F.n.* upregulate IL-1β mRNA/protein via *AIM2* and *NLRP3* inflammasome dysregulation in an OSCC cell line, promoting a pro-tumor inflammatory phenotype [94]. Chronic *P.g.* infection increases *IL-6* secretion via TLR signaling, which activates *STAT1* and *STAT3*, driving tumor progression through a feedback loop involving *CXCL10*, *PD-L1,* and *GAS6* genes. These effects have been observed in both OSCC cells [52] and human immortalized oral keratinocytes [95, 96]. *P.g.* also induces immune evasion by upregulating PD-L1 and PD-L2 in OSCC cells and keratinocytes, impairing anti-tumor immune responses [97–99]. *P.g.* achieves this via outer membrane vesicles that deliver virulence factors to activate RIP2- and MAPK-dependent signaling, leading to PD-L1 overexpression in SCC-25 cells [97]. *Pg*-LPS activated MAPK signaling, mainly through increased expression of p38 and pp38, with pp38 upregulated in SCC9, and p38 in HSC3 [100].

Additionally, *P.g.* exploits DC-SIGN-TLR2/CXCR4 signaling, activating AKT1 and inhibiting FOXO1 by ligation via *P.g.* Mfa1-FimA, thereby promoting tumor-associated immunosuppression via myeloid-derived suppressor cells and Tregs [74]. *P.g.* also manipulates inflammatory signaling in SCC-25 cells by activating NF-κB and MAPK pathways, driving tumor-associated inflammation and metastasis via upregulation of *CCL20*, *TNFAIP6*, *CXCL8*, *TNFAIP3*, *TRAF5*, *CYP1A1*, and *NOD2* gene expression [101]. *P.g.* induces macrophage polarization into tumor-associated M2, upregulating gene expression of pro-tumoral molecules (*suprabasin*, *IL-1R2*, *IL-18*, and *TGF-α*) while suppressing multiple anti-tumor cytokines (*TNF-β*, *IFN-β, TRAIL*, and *TNF-α*) in Cal-27 cells. This process promotes an immunosuppressive tumor microenvironment, facilitating OSCC immune evasion and growth [102]. In addition, extracellular vesicles derived from *F.n.* carry tryptophanase, which stimulates indole production in macrophages and activates the TDO2–AHR signaling pathway [83]. This cascade upregulates IL-10 and immune checkpoint molecules, reducing infiltration of CD8⁺ T cells while increasing regulatory T cells, thereby promoting an immunosuppressive tumor microenvironment that supports tumor progression.

In contrast, some bacteria that are negatively associated with periodontitis, exhibit tumor-suppressive effects. *Neisseria sicca* and *Corynebacterium matruchotii* restrain OSCC development by maintaining genome stability via activation of DNA damage signaling, increasing ATR and CHK1 phosphorylation, and inducing NLRP3/GSDMD-mediated pyroptosis. These bacteria also suppress inflammatory cytokines, including mRNA of *NF*-*κB* and *IL-6* in the tumor microenvironment, and reduce CD4 + and CD206 + T cells in the spleen, contributing to an anti-tumor immune response [103].

*P.g.* and *F.n.* influence cell cycle regulation and proliferation through diverse mechanisms, including G1/S phase arrest, autophagy induction, and cyclin modulation. In OSCC cells, *P.g.* induces G1 cell cycle arrest by downregulating cyclin D1 and cdk4 and upregulating p21, leading to ROS-dependent autophagy and suppression of proliferation [104]. Repeated exposure of OSCC cells to *P.g.* slowed proliferation, conferred chemoresistance, enhanced stemness (CD44/CD133 upregulation), and increased tumor sphere formation [46]. Consistent with this, *P.g.* has been linked to chemoresistance in OSCC cells via Notch1 activation, suggesting a mechanism that enables tumor growth despite treatment [105]. Co-culture with *P.g.* reduced apoptosis in HN4 and CAL27 cells induced by chemotherapeutic agents (cisplatin and TNF-α + SM-164), and in HN4 cells blocked PARP cleavage and phosphorylation of H2AX [106]. In contrast to its inhibitory effects on OSCC proliferation, *P.g.* exhibits tumor-promoting properties in immortalized oral keratinocytes, where infection increases proliferation, S-phase fraction, and proMMP9 secretion, contributing to tumorigenic transformation [95]. In another study, *P.g.* enhances OSCC proliferation via modulation of miR-21/PDCD4/AP-1 signaling, cyclin D1 upregulation and S-phase entry [82]. The outer membrane vesicles of *P.g.* greatly promote proliferation of OSCC cells in a dose-dependent manner [107, 108]. In addition, neutrophil extracellular traps enhanced tumor cell proliferation and invasiveness when stimulated by *P.g.*, and this effect was significantly reduced upon neutrophil extracellular degradation by DNase I [75].

*F.n.* promotes proliferation [46, 86], reducing cells in G1 while increasing S-phase entry, accompanied by p27 and Ku70/p53 downregulation, and facilitating uncontrolled cell cycle progression in OSCC cells [46]. Conflicting evidence suggests that *F.n.* does not impact proliferation; *F.n.* in immortalized oral keratinocytes and SCC-9 did not accelerate cell cycle progression but increased apoptosis [91]. Similarly, extracellular vesicles from *F.n.* inhibited proliferation and increased apoptosis in both SCC-24A and HSC-3 cells [109]. Beyond direct cell cycle effects, *F.n.* alters long-term gene expression in gingiva-derived mesenchymal stem cells, influencing the NLRP3 inflammasome and transcription factors (*CREB3, GATA2, SOX4*) that facilitate remodeling of the microenvironment [110]. Further supporting a role in tumor progression, *P.g.* and *F.n.* inhibit CHK1 activation while upregulating *NLRP3* gene expression in OSCC cells, promoting growth [111]. Additionally, BHY cells (human bone invasive OSCC) respond to bacterial challenges by increasing defensin expression, suggesting a link between infection and tumorigenesis via EGFR-NF-κB [112]. *P.g.* and *Aggregatibacter actinomycetemcomitans (A.a.)* influenced defensin expression; while *A.a.* reduced BHY proliferation by 50% and exhibited mild cytotoxic effects (11%), *P.g.* increased proliferation by 125%, upregulating *hBD-1*, *hBD-2*, *hBD-3* and *HNP-1/3* while downregulating *DEFA4* transcripts. Defensin modulation corresponded with *cyclin D1* transcript upregulation, suggesting a role in proliferation. Moreover, bacterial stimulation increased nuclear NF-κB expression, reinforcing involvement in infection-driven tumorigenesis [112]. Extracellular vesicles from *A.a.* increased proliferation and invasion in HNSCC cell lines (FaDu and UMSCC1) [113]*.* On the other hand, extracellular vesicles from a wild-type *A.a.* strain isolated from a patient with aggressive periodontitis reduced proliferation, invasion, and increased apoptosis in HSC-3 cells, while its mutant derivative lacking the cdtABC had no significant effects. The LPS O-antigen-deficient variant had mixed effects, including a slight reduction in apoptosis in SCC-24A [109]. In addition, *Capnocytophaga gingivalis* promotes EMT-like changes by E-cadherin and β-catenin downregulation and vimentin and SNAIL upregulation without significantly affecting OSCC proliferation [114].

Beyond bacterial infections, microbial metabolites, like sodium butyrate, inhibit proliferation in a dose-dependent manner by altering G1/S progression in OSCC cells [92, 93]. In contrast, *T.d.* enhances OSCC proliferation via upregulation of *TGFβ1, TGFβ2*, and *TGFβ3* gene expression, and activation of the TLR4-MyD88-NF-κB pathway. In *T*.*d.*-infected OSCC cells, the percentage of S + G2 phase cells was enhanced, apoptosis was suppressed, and proliferation increased in a concentration-dependent manner; these effects were reversed by inhibition of *TGF-β* receptor [115].

*P.g.* and *F.n.* enhance metastatic and invasive properties by promoting cell migration, invasion, and remodeling of the extracellular matrix. Both pathogens increase migration in OSCC cells [47, 86, 100, 107, 109], and induce IL-6, IL-8, MMP-9, and Cyclin-D1 expression supporting a role in OSCC progression [54]. *P.g.* and *F.n.* also stimulate tumor-associated neutrophils via CXCL2/CXCR2, accelerating tumor invasion [76]. *P.g.* drives IL-8-mediated invasion by upregulating MMP-1 and MMP-10 in OSCC cells, while IL-8 knockdown suppresses invasion [46, 50]. *P.g.* promotes IL-17 production and upregulation of pro-migratory genes/proteins, e.g. *DUSP10, IL1B,* MMP-2 uPA and uPAR further contributing to invasion [100, 116]. Mechanistically, *P.g.* activates ERK1/2-Ets1, p38/HSP27, and PAR2/NF-κB pathways, increasing proMMP-9 production, while MMP-9 activated by gingipains facilitates invasion [117]. Gingipain-deficient mutants do not induce *PAR2* or *PAR4* transcripts, confirming their role in this process [118]. *P.g.* exploits PD-L1-mediated immune evasion, while bacterial LPS enhances OSCC invasion via TLR4-dependent suppression of E-cadherin [119]. The LPS/TLR4 pathway induces IL-8 secretion but does not directly impact MMP1 or MMP9 expression in OSCC cells, suggesting an alternative invasion-promoting mechanism [120]. *P.g.* can target and degrade DSC2 through outer membrane vesicle-associated sRNA23392, affecting OSCC migration and invasion [121]. Similarly, conditioned medium from macrophages stimulated by *P.g.* LPS promoted invasion of human primary (HN18, HN30 and HN4) and metastatic (HN17, HN31, and HN12) HNSCC cell lines, and inhibited proliferation of HN18, HN30, HN31 and HN4; this effect is linked to increased nitric oxide and decreased TNF-α in macrophages [122]. *P.g.* also recruits myeloid-derived suppressor cells to precancer increasing *CCL2, CXCL2, IL-6,* and *IL-8* gene expression, which promotes migration [81].

In addition to *P.g.*, *F.n.* enhances invasion of OSCC cells in a strain-dependent manner [48]. The *F.n.*-induced change in morphology of primary oral keratinocytes to rounded and dispersed cells suggests transition toward an invasive state [87]. Interestingly, *F.n.* alone had comparable or greater effects than polyinfection with other oral bacteria, emphasizing its impact on invasion [120]. In HSC-3 cells, treatment with dexamethasone, an NF-κB inhibitor, reversed the effects induced by *F.n.*, suppressing EMT-like changes, cell proliferation, invasion, and migration [86]. Beyond bacterial infections, microbial metabolites enhance tumor aggressiveness. Sodium butyrate significantly increases MMP-1, −2, −9, and −13 in OSCC cell lines, facilitating EMT-mediated invasion [92]. Together *P.g.*, *F.n.*, and *T.d.* activate integrin/FAK and TLR/MyD88 pathways, increasing stemness, sphere formation, and metastatic potential of OSCC cells [123]. *T.d.* infection upregulates integrin αV, FAK phosphorylation, and MyD88-dependent signaling, and its suppression prevents FAK phosphorylation and migration [123]. Lipooligosaccharide and LPS from *T.d.*, *P.g.*, and *F.n.* enhance OSCC migration, reinforcing the role of bacterial components in metastasis [123].

Together these studies support the role of *P.g.* and *F.n.* in OSCC progression via EMT, invasion, and immune evasion. Their ability to upregulate EMT transcription factors, enhance mesenchymal markers, and modulate β-catenin signaling, highlights their contribution to metastasis. These pathogens also influence cell cycle regulation and proliferation by inducing G1/S phase arrest, modulating cyclin, and enhancing cancer stemness, contributing to chemoresistance and tumor growth. Their involvement in inflammatory pathway dysregulation and immune suppression reinforces their impact on OSCC progression.

#### Experimental periodontitis and oral microbiota in OSCC (animal studies)

Animal studies using experimental periodontitis models explored the intricate relationship between periodontitis, oral microbiota, and OSCC, revealing significant interactions that impact growth, treatment resistance, and inflammatory responses. *P.g.* exacerbates 4NQO-induced OSCC by altering fatty acid metabolism, increasing FASN and ACC1, and promoting inflammatory cell infiltration; increased crestal bone loss, large tumors, and severe hepatic steatosis were observed in *P.g.*-exposed mice [124]. Similarly, mice with periodontitis treated with 4NQO had lesions with large surface areas, increased bone loss, and higher incidence of precancer than controls [53, 125]. Conversely, periodontitis-negative bacteria did not alter tumor volume induced by SCC-7 in buccal mucosa but decreased angiogenesis and cyclin D1, while increasing γH2AX-positive cells, suggesting a protective role against tumor progression [103].

When co-infected, *F.n.* and *P.g.* increased invasion, proliferation and tumor size, with marked upregulation of cyclin D1. Co-infection also led to extensive hyperplasia, increased nuclear/cytoplasmic ratios, and large lesions [52, 111]. Mice infected with *P.g.* exhibited larger, more proliferative lesions and significant weight loss than controls in a carcinogenesis model [81]. In contrast, *P.g.* delays tumor growth induced by OSC-20 human OSCC cells injected subcutaneously in nude mice while provoking resistance to paclitaxel, likely due to cytokine modulation, particularly IL-6. Although no significant histopathologic differences were observed, tumor xenografts in *P.g.*-treated mice grew slower than controls. However, exposure to paclitaxel increased tumor growth and volume, which were reversed by ibuprofen that significantly modulated cytokine levels, suppressing MCP-1 and increasing TNF-α, IL-2, VEGF, and MMP-9 [126].

In mouse models of periodontitis with SCC7 cells injected in the buccal mucosa [78, 127], γδ T cells affected the gut microbiome and host metabolism. Targeting γδ T cells reduced metabolites associated with cancer promotion while increasing anti-cancer metabolites. In advanced OSCC, increased tumor weight, cell proliferation, and Ki67 staining were observed in periodontitis-affected groups. IL-17A-producing γδ T cells were significantly higher in OSCC with periodontitis; inhibiting these cells reduced tumor development, IL-17A, and M2 macrophages. Additionally, STAT3 phosphorylation was activated in OSCC with periodontitis but declined upon γδ T cell inhibition, suggesting a pivotal role of γδ T cells in tumor progression via IL-17A and STAT3 signaling [78, 127]. Using the same murine SCC7 tumor model, intratumoral administration of *F.n.* outer membrane vesicles promoted tumor growth by suppressing CD8⁺ T cell infiltration and increasing AHR-positive macrophages. These vesicles also upregulated immune checkpoint molecules, including PD-L1, PD-L2, and SIGLEC-15, contributing to an immunosuppressive tumor microenvironment. The reduction in IFN-γ⁺ and TNF-α⁺ CD8⁺ T cells was partially reversed by AHR and phagocytosis inhibition. Notably, the combination of PD-1 monoclonal antibody with a TDO2 inhibitor resulted in a more pronounced antitumor response and greater CD8⁺ T cell infiltration than either treatment alone [83].

Ligature-induced periodontitis combined with *F.n.* promoted growth of subcutaneously injected SCC7 cells, as shown by increased expression of proliferation markers Ki-67 and PCNA. This was associated with reduced infiltration of CD8⁺ and IFN-γ⁺CD8⁺ T cells, and elevated levels of regulatory T cells, M2 tumor-associated macrophages, PD-1⁺CD8⁺ T cells, and PD-L1⁺Tumor-Associated Macrophages in both spleen and tumor. Anti-PD-1 therapy reversed these effects, leading to decreased tumor volume and weight, reduced Ki-67 and PCNA expression, and increased CD8⁺ and IFN-γ⁺CD8⁺ T cell infiltration [79]. Moreover, intraperitoneal administration of *P.g.* significantly enhanced growth of SCC7-induced tumors, promoted lung metastasis, and increased neutrophil extracellular trap-related markers H3cit and MPO, in both implanted and metastatic tumors. These effects were attenuated by DNase I treatment, which suppressed tumor progression, reduced the number of metastatic nodules, and lowered H3cit and MPO levels [75].

The role of *P.g.* in promoting OSCC through autophagy modulation was demonstrated by intratumoral injection of P.g., which significantly increased tumor weight and diameter in a model where OSCC cells were injected subcutaneously into the right axilla of mice. This group exhibited decreased expression of apoptotic and DNA damage markers (cleaved caspase-3 and p-H2AX), reduced p62, and increased LC3A/B, indicating enhanced autophagy. Ki-67 levels were unchanged, suggesting no direct effect on cell proliferation. Treatment with hydroxychloroquine (*P.g.* + HCQ), an autophagy inhibitor, reversed these changes by reducing tumor size, restoring p62 levels, increasing apoptosis, and lowering LC3A/B expression [106]. In addition, treatment with outer membrane vesicles from *P.g.* significantly increased tumor volume induced by SCC-9 cells injected subcutaneously into the right flank, as well as cell proliferation (Ki-67) and expression of EMT markers such as N-cadherin, Vimentin, and MMP9, while reducing E-cadherin expression. Inhibition of NF-κB with BAY 11–7082 reduced both tumor growth and Ki-67 expression, whereas co-treatment with BAY 11–7082 and the ferroptosis-inducer erastin reversed these effects. These results support the role of *P.g.* outer membrane vesicles in OSCC progression via NF-κB–mediated ferroptosis inhibition [107].

*Porphyromonas intermedia* infection promoted growth of SCC-7 cells injected into the buccal submucosa, leading to angiogenesis, muscle invasion, lymph node metastasis, and elevated expression of Ki-67, VEGF-A, IL-6, IL-17, and ISG15. It also enhanced M2 macrophage and Treg infiltration while reducing CD4⁺ and CD8⁺ T cells [128]. These effects were significantly mitigated by antibiotic treatment, which reduced inflammation and slowed tumor progression [128].

Animal studies reinforce the impact of *P.g.* and *F.n.* on OSCC progression, demonstrating their role in enhancing growth, invasion, and proliferation, particularly in the presence of periodontitis. These bacteria upregulate cyclin D1, promote hyperplasia, and alter inflammatory and metabolic pathways, contributing to a tumor-permissive environment. Additionally, *P.g.* influences fatty acid metabolism and cytokine profiles that can drive tumor progression or induce chemoresistance depending on the microenvironment. The involvement of γδ T cells and IL-17A/STAT3 signaling highlights the immunomodulatory effects of periodontitis in OSCC.

### Meta-analysis results

Twenty-six case–control studies evaluating the association between periodontitis and OSCC were included in a meta-analysis, along with an overall assessment of HNSCC. The results revealed significant association between self-reported gingival bleeding (assessed by questionnaire) and HNSCC (n = 5 studies; OR = 3.37, 95% CI: 1.17–9.74; p = 0.02), with high heterogeneity (I^2^ = 93.2%) (Table 4; Supplementary Figure S1). Conversely, no statistically significant association was observed between self-reported gingival bleeding (assessed by questionnaire) and OSCC (n = 3 studies; OR = 5.83, 95% CI: 0.62–54.65; p = 0.12), with high heterogeneity among included studies (I^2^ = 73.6%) (Table 4; Supplementary Figure S2).

**Table 4:** Meta-analysis of the association between periodontitis and oral squamous cell carcinoma prevalence

**Self-reported gingival bleeding by questionnaire (*n=*5)**						
** Random effects model**	**HNSCC**		**No HNSCC**		**OR**	**95%CI**
	**Events**	**Total**	**Events**	**Total**		
	395	930	244	1111	3.37	[1.17; 9.74]
** Heterogeneity**	***I***^2^		**τ**^2^		**p**	
	93.2%		1.1319		<0.0001	
** Test for overall effect**	**Z**			**p**		
	2.25			0.00246		
**Self-reported gingival bleeding by questionnaire (***n=***3)**						
** Random effects model**	**OSCC**		**No OSCC**		**OR**	**95%CI**
	**Events**	**Total**	**Events**	**Total**		
	116	288	107	430	5.83	[0.62; 54.65]
** Heterogeneity**	***I***^2^		**τ**^2^		**p**	
	73.6%		3.07		0.0226	
** Test for overall effect**	**Z**			**p**		
	1.54			0.1227		
**Self-reported gum disease by questionnaire (***n=***8)**						
** Random effects model**	**HNSCC**		**No HNSCC**		**OR**	**95%CI**
	**Events**	**Total**	**Events**	**Total**		
	946	4679	1150	5847	1.30	[0.94; 1.80]
** Heterogeneity**	***I***^2^		**τ**^2^		**p**	
	74.5%		0.1625		0.0003	
** Test for overall effect**	**Z**			**p**		
	1.56			0.1191		
**Self-reported gum disease by questionnaire (***n=***4)**						
** Random effects model**	**OSCC**		**No OSCC**		**OR**	**95%CI**
	**Events**	**Total**	**Events**	**Total**		
	139	2116	280	2376	1.38	[0.47; 4.07]
** Heterogeneity**	***I***^2^		**τ**^2^		**p**	
	88.1%		0.9539		<0.0001	
** Test for overall effect**	**Z**			**p**		
	0.58			0.5591		
**Clinical diagnosis of gingival inflammation (***n=***3)**						
** Random effects model**	**OSCC**		**No OSCC**		**OR**	**95%CI**
	**Events**	**Total**	**Events**	**Total**		
	489	811	281	790	2.84	[2.22; 3.64]
** Heterogeneity**	***I***^2^		**τ**^2^		**p**	
	31%		0.0125		0.23	
** Test for overall effect**	**Z**			**p**		
	8.24			<0.01		
**Clinical diagnosis of periodontitis (***n=***4)**						
** Random effects model**	**OSCC**		**No OSCC**		**OR**	**95%CI**
	**Events**	**Total**	**Events**	**Total**		
	367	483	395	645	3.32	[1.84; 5.99]
** Heterogeneity**	***I***^2^		**τ**^2^		**p**	
	70.5%		0.234		0.0171	
** Test for overall effect**	**Z**			**p**		
	3.97			<0.0001		
**Radiographic bone loss (mm) (***n=***3)**						
** Random effects model**	**OSCC**		**No OSCC**		**WMD**	**95%CI**
	**Total**		**Total**			
	495		384		1.55	[1.33; 1.77]
** Heterogeneity**	***I***^2^		**τ**^2^		**p**	
	22%		0.0127		0.28	
** Test for overall effect**	**Z**			**p**		
	13.91			<0.01		
**Probing depth (mm) (***n=***7)**						
** Random effects model**	**OSCC**		**No OSCC**		**WMD**	**95%CI**
	**Total**		**Total**			
	260		266		2.09	[1.43; 2.76]
** Heterogeneity**	***I***^2^		**τ**^2^		**p**	
	92%		0.7418		<0.0001	
** Test for overall effect**	**Z**			**p**		
	6.17			<0.0001		
**Clinical attachment level (mm) (***n=***5)**						
** Random effects model**	**OSCC**		**No OSCC**		**WMD**	**95%CI**
	**Total**		**Total**			
	240		211		3.33	[2.85; 3.81]
** Heterogeneity**	***I***^2^		**τ**^2^		**p**	
	84.2%		0.2502		<0.0001	
** Test for overall effect**	**Z**			**p**		
	13.55			<0.0001		
**Bleeding on probing (%) (***n=***6)**						
** Random effects model**	**OSCC**		**No OSCC**		**WMD**	**95%CI**
	**Total**		**Total**			
	252		240		20.74	[11.65; 29.83]
** Heterogeneity**	***I***^2^		**τ**^2^		**p**	
	72.8%		75.5401		0.0053	
** Test for overall effect**	**Z**			**p**		
	4.47			<0.0001		

*HNSCC* Head and neck squamous cell carcinoma, *OSCC *oral squamous cell carcinoma, *OR *odds ratio, *WMD *weighted mean difference; 95% CI: 95% confidence intervals

Self-reported gum disease assessed by questionnaire, did not demonstrate significant association with HNSCC (n = 8 studies; OR = 1.3, 95% CI: 0.94–1.8; p = 0.11), with high heterogeneity (I^2^ = 74.5%) (Table 4; Supplementary Figure S3). Similarly, self-reported gum disease was not associated with increased risk of OSCC (n = 4 studies; OR = 1.38, 95% CI: 0.47–4.07; p = 0.55), with high heterogeneity (I^2^ = 88.1%) (Table 4; Supplementary Figure S4).

Gingival inflammation evaluated by clinical examination was associated with increased risk of OSCC (n = 3 studies; OR = 2.84, 95% CI: 2.22–3.64; p < 0.01), with moderate heterogeneity (I^2^ = 31%) (Table 4; Supplementary Figure S5). Moreover, positive association was identified between periodontitis, assessed by clinical examination, and OSCC (*n *= 4 studies; OR = 3.32, 95% CI: 1.84–5.99; p < 0.0001), although with high heterogeneity (I^2^ = 70.5%) (Table 4; Supplementary Figure S6).

For periodontal clinical parameters, higher radiographic bone loss (n = 3 studies) was observed in patients with OSCC than in those without (WMD = 1.55, 95% CI: 1.33–1.77; p < 0.01), with low heterogeneity (I^2^ = 22%) (Table 4; Supplementary Figure S7). Similar findings were reported for periodontal probing depth (mm) (*n *= 7 studies; WMD = 2.09, 95% CI: 1.43–2.76; p < 0.0001; I^2^ = 92%; Table 4; Supplementary Figure S8), clinical attachment level (mm) (*n *= 5 studies; WMD = 3.33, 95% CI: 2.85–3.81; p < 0.0001; I^2^ = 84.2%; Table 4; Supplementary Figure S9), and bleeding on probing (%) (*n *= 6 studies; WMD = 20.74, 95% CI: 11.65–29.83; p < 0.0001; I^2^ = 72.8%; Table 4; Supplementary Figure S10). High heterogeneity was observed for all these periodontal parameters evaluated.

## Discussion

The goal of this systematic review was to clarify association between periodontitis and OSCC by integrating findings from epidemiological and mechanistic studies. Despite methodological variability between included studies, our meta-analysis supports an association between both clinical and self-reported measures of periodontitis and increased OSCC risk, though with varying degrees of heterogeneity. These clinical associations are reinforced by experimental evidence showing that periodontitis pathogens like *P.g.* and *F.n.* contribute to OSCC progression via inflammation, EMT, and immune evasion. Collectively, these findings suggest a potential biological link between periodontal disease and OSCC.

Previous meta-analyses have also demonstrated positive associations between periodontitis and head and neck cancer (subtype not specified). Gopinath et al. [129], including only studies with objective assessments of clinical periodontal parameters, reported a significant association with head and neck cancer (OR = 3.17), which remained robust after adjusting for smoking and alcohol. A subgroup analysis of studies using alveolar bone loss showed a similar association (OR = 3.54) with lower heterogeneity. Zeng et al. [130] found significant overall association with head and neck cancer (OR = 2.63), but did not distinguish between self-reported and clinical assessments. In a subgroup analysis restricted to alveolar bone loss, the association remained significant (OR = 2.73). Yao et al. [131] also reported significant association between periodontitis and oral cancer (OR = 3.53), but did not separate results by type of assessment or type of oral cancer. In contrast, our review separately analyzes self-reported indicators (such as gingival bleeding and gum disease) and clinical parameters, including radiographic bone loss, probing depth, clinical attachment level, bleeding on probing, as well as gingival inflammation and periodontitis assessed by clinical examination, while also distinguishing between HNSCC and OSCC. Despite high heterogeneity across outcomes, the overall evidence from human association studies supports a positive link between periodontitis and cancer risk, observed both in the pooled analysis of all HNSCC subsites and in the subgroup analysis specific to OSCC.

Expanding on these epidemiological findings, mechanistic studies explored plausible pathways by which periodontitis may drive initiation, progression, and poor prognosis of OSCC. In human studies, *P.g.* and *F.n.* have been frequently identified in subgingival plaque and OSCC tissue, with abundance positively correlated [15]. This supports the hypothesis that bacteria in tumor tissue, but absent from adjacent non-tumor tissue, may originate from patients’ periodontal pockets [71]. In addition, OSCC samples from patients with periodontitis had less IFN-γ⁺ CD8⁺ T cells and higher PD-L1 expression, suggesting a more immunosuppressive tumor environment than those without periodontitis [79]. *P.g.* may contribute to OSCC recurrence by upregulating DOK3 in tumor-associated macrophages, activating TNF and MAPK signaling [72]. DOK3 acts as a key modulator of innate immune responses and may promote aggressive tumor behavior by shaping a macrophage-rich immune microenvironment [72]. Immune alterations also include increased CD4 +/CD8 + and IL-17 + γδ T cells, and elevated expression of immunosuppressive (*IDO1, IL-10, PD-L1*) and oncogenic (*AKT1, STAT3, CXCR4*) genes with key roles in tumor progression and immune evasion [73, 74, 78]. IL-17 + γδ T cells directly promote progression via immunosuppressive effects, particularly via IL-17/STAT3 [78]. STAT3 is a transcription factor essential for tumor cell proliferation [78]. Moreover, periodontitis-associated oral microbiota may drive activation of IL-17 + γδ T cells, creating a pathway that links chronic inflammation to tumor growth [78].

Downregulation of tumor suppressors, such as FOXO3 and IRF1, and increased FOXP3 and pAKT1, were observed [74]. Interestingly, *P.g.* appears to exploit two distinct fimbrial pathways: Mfa1-DC-SIGN, which promotes immunosuppression via pAKT1–pFOXO1–STAT3–IDO1–FOXP3 leading to Treg activation and myeloid-derived suppressor cell differentiation, and FimA that activates oncogenes such as CXCR4 and SDF-1, and silences tumor suppressors like LDOC1 and FOXO3 [74]. In advanced-stage OSCC, higher *P.g.*, neutrophil extracellular traps, CXCL2, and tumor-associated neutrophils correlated with poor prognosis [45, 75, 76]. Recruitment of tumor-associated neutrophils by *P.g.*-infected tumors amplifies inflammatory cytokine release and reinforces positive feedback to promote tumor progression [76].

*In vitro* studies support multifactorial roles for *P.g.* and *F.n.* in OSCC by promoting EMT, deregulating proliferation, and enabling immune evasion. EMT via upregulation of transcription factors Snail and ZEB1, E-cadherin internalization, and cadherin switching, enhances motility/invasion [47, 87, 88, 91]. *P.g.*’s gingipains, particularly RgpA/B, promote nuclear translocation of β-catenin via GSK3β inhibition, inducing EMT-related targets such as MMP-2, −3, and −9 that degrade extracellular matrix and support metastasis [47, 89]. FimA fimbriae also induce ZEB1 in non-transformed keratinocytes, reinforcing a potential role in early carcinogenesis [88]. In parallel, these pathogens affect proliferation via modulation of cyclin D1, p21, and p27, although outcomes vary, ranging from apoptosis to proliferation, depending on strain, bacterial viability, and type of tumor cells [46, 91, 104, 132]. These discrepancies, especially regarding *P.g.’s* dual impact on cell survival, likely reflect complex host–pathogen dynamics [104]. While cyclin D1 is commonly elevated in OSCC, its expression may be suppressed by commensals such as *Neisseria sicca* and *Corynebacterium matruchotii*, suggesting protective microbial influences [103]. Meanwhile, *P.g.* fosters stemness, autophagy, and chemoresistance [82, 105, 106], while amplifying IL-6 and inflammasome signaling (AIM2, NLRP3), thus sustaining STAT3-mediated inflammation [52, 94–96]. Immunoescape is reinforced via PD-L1/PD-L2 overexpression [96, 97], with implications for metastasis and poor prognosis [119]. Additionally, microbial outer membrane vesicles and metabolites like sodium butyrate promote EMT [92] while dysregulation of defensins (e.g., increased hBD-1/2/3, decreased DEFA4) promotes EGFR–NF-κB activation and proliferation [112]. Moreover, *P.g.* outer membrane vesicles promote EMT and OSCC progression via NF-κB–mediated ferroptosis inhibition [107]. *T.d.* also participates in this network, enhancing EMT and cell cycle progression via TGF-β and TLR4–MyD88–NF-κB pathways [115].

Animal studies support a causal role of periodontopathogens in OSCC. Chronic oral infection with *P.g.* accelerates 4NQO-induced murine oral carcinogenesis by enhancing inflammation, fatty acid metabolism, and cyclin D1 expression, leading to large tumors and alveolar bone loss [52, 53, 111, 124]. Co-infection with *F.n.* exacerbates tumor growth and invasion [52, 111]. Mechanistically, this progression was linked to activation of γδ T cells and STAT3 [78, 127]. Furthermore, *F.n.*, *P.g.* and *Porphyromonas intermedia* promote OSCC progression in murine models via immunosuppression [79, 83, 128], enhanced proliferation [79, 106, 128]), angiogenesis [128], EMT [107], and activation of pathways such as TDO2-AHR [83], NF-κB [107], autophagy [106], and neutrophil extracellular trap formation [75]. These effects were partially reversed by anti-PD-1 therapy [79], TDO2 inhibition [83], DNase I [75], antibiotics [128], hydroxychloroquine [106] and BAY 11–7082 [107]. Conversely, commensal bacteria such as *Neisseria sicca* and *Corynebacterium matruchotii* are tumor-suppressive by promoting DNA damage responses and attenuating inflammatory signaling [103], suggesting that microbiome composition can influence OSCC outcomes.

Taken together, these findings suggest that targeting microbiome-driven immunological pathways may offer therapeutic benefits in management of chronic periodontitis and OSCC [76, 78, 121]. Modulation of oral commensal bacteria and immune mediators such as IL-17–producing γδ T cells or CXCL2/CXCR2 could aid in monitoring OSCC progression and in developing adjunct therapies to mitigate tumor-promoting inflammation [76, 78, 121]. Emerging evidence indicates that periodontal pathogens, particularly *P.g.*, may undermine efficacy of conventional anti-cancer therapies by promoting chemoresistance and enhancing tumor adaptability via pathways regulated by Notch1 and IL-6 [46, 105, 106, 126]. *P.g.*-mediated reduction of tumor cell proliferation while promoting stemness, survival, and resistance, illustrates that chronic microbial inflammation could impair treatment response [46, 104]. Such findings emphasize the therapeutic relevance of integrating periodontal management in cancer care, particularly for patients with refractory or metastatic disease [126]. However, it is unknown whether periodontal treatment can influence OSCC development, progression, or therapeutic response.

This review has several limitations due to heterogeneity and methodological gaps across studies. The main challenge with human studies is variability in parameters used to diagnose periodontitis. While some relied on self-reported diagnoses or presence of clinical signs, others used radiographic bone loss without considering inflammatory status, such as bleeding on probing, of periodontal tissue [22]. Moreover, definitions based solely on marginal bone loss identified on radiographs have limitations due to lack of specificity and often failure to detect early or moderate disease [133]. Although self-reported questionnaires show acceptable validity for identifying periodontitis, sensitivity is generally lower than specificity, so some cases may go undetected, possibly due to lack of patient awareness or recall [134]. Importantly, the lack of long-term longitudinal studies limits the ability to establish temporality or causality between periodontitis and cancer, monitor disease progression, control confounding factors, or evaluate the impact of periodontal treatment on patient prognosis [135]. An alternative explanation is that periodontitis may have developed secondary to OSCC, rather than preceding it, highlighting the potential for reverse causality in cross-sectional studies [135]. OSCC itself may impair oral hygiene, alter the local environment, or cause pain during chewing or toothbrushing, factors that can promote biofilm accumulation, inflammation, and ultimately, periodontal breakdown. Some studies did not identify or appropriately control for confounding variables, which may bias observed associations [16, 71]. While many studies detected periodontopathogens in tumor tissues, frequently these findings were not correlated with subgingival biofilm composition or clinical periodontal status. Finally, site-specific differences in association could not be evaluated due to data limitations, including inability to assess proximity of OSCC to periodontal pockets, which may influence risk.

*In vitro* studies exhibited several limitations, including absence of systemic interactions, lack of tumor microenvironment, simplified microbial conditions, and high variability in experimental designs [136]. Similarly, animal studies had limitations such as tumor induction at non-oral sites, use of non OSCC cell lines (e.g., SCC-7) [70], and extensive variation in methods to induce OSCC and periodontitis, which complicates data comparison and interpretation across studies.

Future research should address current methodological Limitations by implementing more rigorous and standardized approaches. In Human studies, there is great need for standardized clinical assessments of periodontitis, ideally following the 2018 periodontitis classification system, which incorporates staging and grading to improve diagnosis [20]. Incorporating stage and grade with clinical parameters such as clinical attachment loss, probing depth, and bleeding on probing, would enhance accuracy [133]. Furthermore, future studies should report OSCC sub-sites and assess local periodontal conditions to enable investigation of whether site-specific disease severity and anatomical proximity to periodontal lesions modulate cancer risk. Additionally, long-term longitudinal studies are essential to clarify temporal relationships and causality. Investigations should also explore correlation between periodontopathogens in biofilm, saliva, and tissue, with OSCC risk, progression, and prognosis. Moreover, strategies to control for confounding factors, such as age, gender, smoking, alcohol consumption, and systemic health conditions, are essential. *In vitro*, use of OSCC cell Lines and 3D culture models that mimic the tumor microenvironment and immune interactions, would improve relevance and reproducibility of findings [136]. For *in vivo* studies, future work should compare different models of periodontitis and OSCC to identify the most suitable approaches for studying interaction, particularly those that best replicate natural progression.

In conclusion, this systematic review provides evidence supporting an association between periodontitis and OSCC, grounded in epidemiology data and mechanistic insights. While studies suggest a biological link mediated by chronic inflammation, EMT, and immune evasion driven by key periodontopathogens, these should be interpreted with caution due to heterogeneity in methods, limited longitudinal data, and variability in periodontitis assessment across studies. Future studies using standardized diagnostic criteria, robust design, and clinically-relevant experimental models, are essential for causal inferences and improved understanding of underlying mechanisms of association between periodontitis and OSCC.

## Supplementary Information

Below is the link to the electronic supplementary material.

Supplementary file1 (DOCX 27 KB)

Supplementary file2 (XLSX 169 KB)

Supplementary file3 (DOCX 1506 KB)

## Data Availability

No datasets were generated or analysed during the current study.
