# Supplementary material for: Demystifying the link between periodontitis and oral cancer: a systematic review integrating clinical, pre-clinical, and in vitro data
Source: Cancer Metastasis Rev. 2025 Sep 9;44(3):67. doi: 10.1007/s10555-025-10285-z (PMC12420769; doi:10.1007/s10555-025-10285-z)
Supplement: Supplementary file 1 — Supplementary file1 (DOCX 27 KB) [file 10555_2025_10285_MOESM1_ESM.docx]

**SUPPLEMENTARY MATERIAL**

**List of proteins/genes abbreviations**

γ-H2AX Gamma H2A histone family member X

*A.a Aggregatibacter actinomycetemcomitans*

AHR Aryl hydrocarbon receptor

AIM2 Absent in Melanoma 2

AKT Protein Kinase B

AP-1 Activator Protein 1

ARG1 Arginase 1

ASC Apoptosis-associated speck-like protein containing a CARD

ATR Ataxia Telangiectasia and Rad3-Related

BIM Bcl-2-interacting mediator of cell death

c-Jun Cellular Jun proto-oncogene

CCL20 C-C motif chemokine ligand 20

CD Cluster of Differentiation

CDK4 Cyclin dependent kinase

CHK1 Checkpoint Kinase 1

CREB3 cAMP responsive element binding protein 3

CTLA-4 Cytotoxic T-Lymphocyte Antigen 4

CTGF Connective tissue growth factor

CXCL C-X-C motif chemokine ligand

CXCR C-X-C chemokine receptors

CYP1A1 Cytochrome P450 family 1 subfamily A member 1

DC-SIGN Dendritic Cell-Specific Intercellular Adhesion Molecule-3-Grabbing Non-Integrin

DEFA Human α-defensins

DOK3 Docking Protein 3

DSC2 Desmocollin-2

DUSP10 Dual specificity phosphatase 10

EGR3 Early growth response 3

ERK Extracellular Signal-Regulated Kinase

Ets1 ETS Proto-Oncogene 1, Transcription Factor

F4/80 EGF-like module-containing mucin-like hormone receptor-like 1

FadA Fusobacterium Adhesin A

FAK Focal Adhesion Kinase

FASN Fatty Acid Synthase

FimA Fimbrial Adhesin A

FOXO Forkhead Box O

FOXP3 Forkhead Box Protein 3

Gal-GalNAc D-galactose-β-N-acetyl-D-galactosamine

GAPDH Glyceraldehyde 3-phosphate dehydrogenase

GAS6 Growth arrest specific 6

GATA2 Guanina-adenina-timina-adenina -binding protein 2

GSDMD Gasdermin D

GSK3β Glycogen synthase kinase 3

H2AX H2A histone family member X

H3cit Histone H3 Citrullination

Hbd Human β-defensins

HBEGF Heparin binding EGF like growth factor

HNP Human Neutrophil Peptide

HSP27 Heat Shock Protein 27

IFN-γ Interferon gama

IDO1 Indoleamine 2,3-dioxygenase 1

IL Interleukin

IRF1 Interferon regulatory factor 5

ISG15 ISG15 ubiquitin like modifier

JAK1 Janus Kinase 1

Jun Jun proto-oncogene

Kgp Lysine-specific gingipain

Ki-67 Ki-67 antigen

Ku70 ATP-dependent DNA helicase

LC3 Microtubule-Associated Proteins 1A/1B Light Chain 3

LDH Lactate dehydrogenase

LDOC1 Leucine Zipper Downregulated in Cancer 1

LOS Lipooligosaccharide

LPS Lipopolysaccharides

LTSCCAT LPS-induced TSCC-associated transcript

mAb Monoclonal antibody

MAPK Mitogen-activated protein kinase

MCP-1 Monocyte chemoattractant protein-1

Mfa1 Mating Factor A1

miR-21 MicroRNA-21

miR-296-5P MicroRNA

MIR4435-2HG MicroRNA 4435-2 Host Gene

MMP Matrix metalloproteinase

MPO Mieloperoxidase

MyD88 Myeloid Differentiation Primary Response 88

NF- κB Transcription factor nuclear factor-kappa B

NLRP3 NOD-like receptor family, pyrin domain containing 3

NOD2 nucleotide binding oligomerization domain containing 2

p21 Cyclin-Dependent Kinase Inhibitor 1A

p38 Mitogen-activated protein kinase 1

p53 Tumor Protein 53

pAKT1 Phosphorylated AKT1

PAR Proteinase-activated receptor

PARP Polyadenosine-diphosphate-ribose polymerase

PCNA Proliferating cell nuclear antigen

PDCD4 Programmed Cell Death 4

PD-1 Programmed cell death 1

PD-L1 Programmed cell death 1 ligand

pFOXO1 Phosphorylated Forkhead Box O1

PLAU Plasminogen activator urokinase

PTGS2 Prostaglandin-endoperoxide synthase 2

RAD51 RAD51 recombinase

RIP2 Receptor-interacting protein kinase 2

SDF1 Stromal Cell-Derived Factor 1

SEMA7A Semaphorin 7A

SGK1 Serum/Glucocorticoid Regulated Kinase 1

SIGLEC-15 Sialic acid-binding immunoglobulin-like lectin 15

SLUG Snail family transcriptional repressor 2

SMYD3 SET and MYND domain-containing protein 3

SNAI1 Snail family transcriptional repressor 1

SNAIL Snail family transcriptional repressor 1

SOCS Suppressor of Cytokine Signaling

SOX4 SRY-Box Transcription Factor 4

STAT Signal transducers and activators of transcription

TDO2 Tryptophan 2,3-dioxygenase

TGF Transforming growth factor

TLR Toll like receptor

TNF Tumor necrosis factor

TNFAIP Tumor Necrosis Factor Alpha-Induced Proteins

TRAF5 TNF receptor-associated factor 5

TRAIL TNF-Related Apoptosis-Inducing Ligand

Twist Twist family bHLH transcription factor 1

uPA Urokinase plasminogen activator

uPAR Urokinase plasminogen receptor

VEGF Vascular endothelial growth factor

ZEB Zinc finger E-box binding homeobox
