# Supplementary material for: Demystifying the link between periodontitis and oral cancer: a systematic review integrating clinical, pre-clinical, and in vitro data
Source: Cancer Metastasis Rev. 2025 Sep 9;44(3):67. doi: 10.1007/s10555-025-10285-z (PMC12420769; doi:10.1007/s10555-025-10285-z)
Supplement: Supplementary file 3 — Supplementary file3 (DOCX 1506 KB) [file 10555_2025_10285_MOESM3_ESM.docx]

**SUPPLEMENTARY MATERIALS**

**MATERIALS AND METHODS**

**Literature search**

| Table S1: Strategies for database search | |
| --- | --- |
| **Database** | **Search Strategy (June 2025)** |
| **PubMed/**  **MEDLINE** | **#1**(((("Periodontal Diseases"[All Fields] OR "disease periodontal"[All Fields] OR "diseases periodontal"[All Fields] OR "Periodontal Disease"[All Fields] OR "Periodontitis"[All Fields] OR "Periodontal Attachment Loss"[All Fields] OR "attachment loss periodontal"[All Fields] OR "loss periodontal attachment"[All Fields] OR "bone loss periodontal"[All Fields] OR ("alveolar bone loss"[MeSH Terms] OR ("alveolar"[All Fields] AND "bone"[All Fields] AND "loss"[All Fields]) OR "alveolar bone loss"[All Fields] OR ("bone"[All Fields] AND "losses"[All Fields] AND "periodontal"[All Fields])) OR "Periodontal Bone Losses"[All Fields] OR "Periodontal Bone Loss"[All Fields] OR "Periodontal Resorption"[All Fields] OR ("alveolar bone loss"[MeSH Terms] OR ("alveolar"[All Fields] AND "bone"[All Fields] AND "loss"[All Fields]) OR "alveolar bone loss"[All Fields] OR ("periodontal"[All Fields] AND "resorptions"[All Fields])) OR "resorption periodontal"[All Fields] OR "Periodontal Pocket"[All Fields] OR "pocket periodontal"[All Fields] OR "Periodontal Pockets"[All Fields] OR "pockets periodontal"[All Fields]) |
|  | **#2** ("carcinoma squamous cell"[All Fields] OR "carcinomas squamous cell"[All Fields] OR "Squamous Cell Carcinomas"[All Fields] OR "Squamous Cell Carcinoma"[All Fields] OR "carcinoma squamous"[All Fields] OR "carcinomas squamous"[All Fields] OR "Squamous Carcinoma"[All Fields] OR "Squamous Carcinomas"[All Fields] OR "Squamous Cell Carcinoma of Head and Neck"[All Fields] OR "Head And Neck Squamous Cell Carcinomas"[All Fields] OR "squamous cell carcinoma head and neck"[All Fields] OR "Squamous Cell Carcinoma of the Head and Neck"[All Fields] OR "Head and Neck Squamous Cell Carcinoma"[All Fields] OR "HNSCC"[All Fields] OR "carcinoma squamous cell of head and neck"[All Fields] OR "Squamous Cell Carcinoma of the Larynx"[All Fields] OR "Laryngeal Squamous Cell Carcinoma"[All Fields] OR "Squamous Cell Carcinoma of Larynx"[All Fields] OR "Oral Tongue Squamous Cell Carcinoma"[All Fields] OR "Hypopharyngeal Squamous Cell Carcinoma"[All Fields] OR "Oral Squamous Cell Carcinoma"[All Fields] OR "Oral Cavity Squamous Cell Carcinoma"[All Fields] OR "Oral Squamous Cell Carcinomas"[All Fields] OR "Squamous Cell Carcinoma of the Mouth"[All Fields] OR "Oropharyngeal Squamous Cell Carcinoma"[All Fields])) |
|  | **#3** "Skin"[All Fields]) |
|  | **#4** ("Lung"[All Fields] OR "Lungs"[All Fields])) |
|  | **#5** "Esophagus"[All Fields] |
|  | **#1 AND #2 AND #3 AND NOT #4 AND NOT #5** |
| **Embase** | **#1** 'periodontal diseases'/exp OR 'periodontal diseases' OR 'disease, periodontal' OR 'diseases, periodontal' OR 'periodontal disease' OR 'periodontitis' OR 'periodontal attachment loss' OR 'attachment loss, periodontal' OR 'loss, periodontal attachment' OR 'bone loss, periodontal' OR 'bone losses, periodontal' OR 'periodontal bone losses' OR 'periodontal bone loss' OR 'periodontal resorption' OR 'periodontal resorptions' OR 'resorption, periodontal' OR 'periodontal pocket' OR 'pocket, periodontal' OR 'periodontal pockets' OR 'pockets, periodontal' |
|  | **#2** 'carcinoma, squamous cell'/exp OR 'carcinoma, squamous cell' OR 'carcinomas, squamous cell' OR 'squamous cell carcinomas' OR 'squamous cell carcinoma' OR 'carcinoma, squamous' OR 'carcinomas, squamous' OR 'squamous carcinoma' OR 'squamous carcinomas' OR 'squamous cell carcinoma of head and neck' OR 'head and neck squamous cell carcinomas' OR 'squamous cell carcinoma, head and neck' OR 'squamous cell carcinoma of the head and neck' OR 'head and neck squamous cell carcinoma' OR 'hnscc' OR 'carcinoma, squamous cell of head and neck' OR 'squamous cell carcinoma of the larynx' OR 'laryngeal squamous cell carcinoma' OR 'squamous cell carcinoma of larynx' OR 'oral tongue squamous cell carcinoma' OR 'hypopharyngeal squamous cell carcinoma' OR 'oral squamous cell carcinoma' OR 'oral cavity squamous cell carcinoma' OR 'oral squamous cell carcinomas' OR 'squamous cell carcinoma of the mouth' OR 'oropharyngeal squamous cell carcinoma' |
|  | **#3** 'skin'/exp OR skin |
|  | **#4** 'lung'/exp OR 'lung' OR lungs |
|  | **#5** 'esophagus'/exp OR esophagus |
|  | **#1 AND #2 AND #3 AND NOT #4 AND NOT #5** |
| **Web of Science** | **#1** (((((((((((((((((((ALL=(“Periodontal Diseases”)) OR ALL=(“Disease, Periodontal”)) OR ALL=(“Diseases, Periodontal”)) OR ALL=(“Periodontal Disease”)) OR ALL=(“Periodontitis”)) OR ALL=(“Periodontal Attachment Loss”)) OR ALL=(“Attachment Loss, Periodontal”)) OR ALL=(“Loss, Periodontal Attachment”)) OR ALL=(“Bone Loss, Periodontal”)) OR ALL=(“Bone Losses, Periodontal”))) OR ALL=(“Periodontal Bone Losses”)) OR ALL=(“Periodontal Bone Loss”)) OR ALL=(“Periodontal Resorption”)) OR ALL=(“Periodontal Resorptions”)) OR ALL=(“Resorption, Periodontal”)) OR ALL=(“Periodontal Pocket”)) OR ALL=(“Pocket, Periodontal”)) OR ALL=(“Periodontal Pockets”)) OR ALL=(“Pockets, Periodontal”) |
|  | #2 ((((((((((((((((((((((((ALL=(“Carcinoma, Squamous Cell”)) OR ALL=(“Carcinomas, Squamous Cell”)) OR ALL=(“Squamous Cell Carcinomas”)) OR ALL=(“Squamous Cell Carcinoma”)) OR ALL=(“Carcinoma, Squamous”)) OR ALL=(“Carcinomas, Squamous”)) OR ALL=(“Squamous Carcinoma”)) OR ALL=(“Squamous Carcinomas”)) OR ALL=(“Squamous Cell Carcinoma of Head and Neck”)) OR ALL=(“Head And Neck Squamous Cell Carcinomas”)) OR ALL=(“Squamous Cell Carcinoma, Head And Neck”)) OR ALL=(“Squamous Cell Carcinoma of the Head and Neck”)) OR ALL=(“Head and Neck Squamous Cell Carcinoma”)) OR ALL=(“HNSCC”)) OR ALL=(“Carcinoma, Squamous Cell of Head and Neck”)) OR ALL=(“Squamous Cell Carcinoma of the Larynx”)) OR ALL=(“Laryngeal Squamous Cell Carcinoma”)) OR ALL=(“Squamous Cell Carcinoma of Larynx”)) OR ALL=(“Oral Tongue Squamous Cell Carcinoma”)) OR ALL=(“Hypopharyngeal Squamous Cell Carcinoma”)) OR ALL=(“Oral Squamous Cell Carcinoma”)) OR ALL=(“Oral Cavity Squamous Cell Carcinoma”)) OR ALL=(“Oral Squamous Cell Carcinomas”)) OR ALL=(“Squamous Cell Carcinoma of the Mouth”)) OR ALL=(“Oropharyngeal Squamous Cell Carcinoma”) |
|  | **#3** ALL=(“Skin”) |
|  | **#4** (ALL=(“Lung”)) OR ALL=(“Lungs”) |
|  | **#5** ALL=(“Esophagus”) |
|  | **#1 AND #2 AND #3 AND NOT #4 AND NOT #5** |
| **Scopus** | **#1**("Periodontal Diseases" OR "Disease, Periodontal" OR "Diseases, Periodontal" OR "Periodontal Disease" OR "Periodontitis" OR "Periodontal Attachment Loss" OR "Attachment Loss, Periodontal" OR "Loss, Periodontal Attachment" OR "Bone Loss, Periodontal" OR "Bone Losses, Periodontal" OR "Periodontal Bone Losses" OR "Periodontal Bone Loss" OR "Periodontal Resorption" OR "Periodontal Resorptions" OR "Resorption, Periodontal" OR "Periodontal Pocket" OR "Pocket, Periodontal" OR "Periodontal Pockets" OR "Pockets, Periodontal" ) |
|  | **#2** ( "Carcinoma, Squamous Cell" OR "Carcinomas, Squamous Cell" OR "Squamous Cell Carcinomas" OR "Squamous Cell Carcinoma" OR "Carcinoma, Squamous" OR "Carcinomas, Squamous" OR "Squamous Carcinoma" OR "Squamous Carcinomas" OR "Squamous Cell Carcinoma of Head and Neck" OR "Head And Neck Squamous Cell Carcinomas" OR "Squamous Cell Carcinoma, Head And Neck" OR "Squamous Cell Carcinoma of the Head and Neck" OR "Head and Neck Squamous Cell Carcinoma" OR "HNSCC" OR "Carcinoma, Squamous Cell of Head and Neck" OR "Squamous Cell Carcinoma of the Larynx" OR "Laryngeal Squamous Cell Carcinoma" OR "Squamous Cell Carcinoma of Larynx" OR "Oral Tongue Squamous Cell Carcinoma" OR "Hypopharyngeal Squamous Cell Carcinoma" OR "Oral Squamous Cell Carcinoma" OR "Oral Cavity Squamous Cell Carcinoma" OR "Oral Squamous Cell Carcinomas" OR "Squamous Cell Carcinoma of the Mouth" OR "Oropharyngeal Squamous Cell Carcinoma" ) AND NOT ( "Skin" ) AND NOT ( "Lung" OR "Lungs" ) AND NOT ( "Esophagus") |
|  | **#3** ( "Skin" ) |
|  | **#4** ( "Lung" OR "Lungs" ) |
|  | **#5** ( "Esophagus" ) |
|  | **#6** LIMIT-TO ( DOCTYPE , "ar" ) OR LIMIT-TO ( DOCTYPE , "re" ) ) |
|  | **#1 AND #2 AND #3 AND NOT #4 AND NOT #5 AND #6** |
| **The Cochrane Library** | **#1** ("Periodontal Diseases" OR "Disease, Periodontal" OR "Diseases, Periodontal" OR "Periodontal Disease" OR "Periodontitis" OR "Periodontal Attachment Loss" OR "Attachment Loss, Periodontal" OR "Loss, Periodontal Attachment" OR "Bone Loss, Periodontal" OR "Bone Losses, Periodontal" OR "Periodontal Bone Losses" OR "Periodontal Bone Loss" OR "Periodontal Resorption" OR "Periodontal Resorptions" OR "Resorption, Periodontal" OR "Periodontal Pocket" OR "Pocket, Periodontal" OR "Periodontal Pockets" OR "Pockets, Periodontal") |
|  | #2 ("Carcinoma, Squamous Cell" OR "Carcinomas, Squamous Cell" OR "Squamous Cell Carcinomas" OR "Squamous Cell Carcinoma" OR "Carcinoma, Squamous" OR "Carcinomas, Squamous" OR "Squamous Carcinoma" OR "Squamous Carcinomas" OR "Squamous Cell Carcinoma of Head and Neck" OR "Head And Neck Squamous Cell Carcinomas" OR "Squamous Cell Carcinoma, Head And Neck" OR "Squamous Cell Carcinoma of the Head and Neck" OR "Head and Neck Squamous Cell Carcinoma" OR "HNSCC" OR "Carcinoma, Squamous Cell of Head and Neck" OR "Squamous Cell Carcinoma of the Larynx" OR "Laryngeal Squamous Cell Carcinoma" OR "Squamous Cell Carcinoma of Larynx" OR "Oral Tongue Squamous Cell Carcinoma" OR "Hypopharyngeal Squamous Cell Carcinoma" OR "Oral Squamous Cell Carcinoma" OR "Oral Cavity Squamous Cell Carcinoma" OR "Oral Squamous Cell Carcinomas" OR "Squamous Cell Carcinoma of the Mouth" OR "Oropharyngeal Squamous Cell Carcinoma") |
|  | **#3** ("Skin") |
|  | **#4** ("Lung" OR "Lungs") |
|  | **#5** ("Esophagus") |
|  | **#1 AND #2 AND #3 AND NOT #4 AND NOT #5** |

**Quality assessment**

Table S2: Joanna Briggs Institute critical appraisal checklist for case-control studies.

| **CHECKLIST** | Yes | No | Unclear |
| --- | --- | --- | --- |
| 1.Were the groups comparable other than the presence of disease in cases or the absence of disease in controls? |  |  |  |
| 2.Were cases and controls matched appropriately? |  |  |  |
| 3.Were the same criteria used for identification of cases and controls? |  |  |  |
| 4.Was exposure measured in a standard, valid and reliable way? |  |  |  |
| 5.Was exposure measured in the same way for cases and controls? |  |  |  |
| 6.Were confounding factors identified? |  |  |  |
| 7.Were strategies to deal with confounding factors stated? |  |  |  |
| 8.Were outcomes assessed in a standard, valid and reliable way for cases and controls? |  |  |  |
| 10.Was appropriate statistical analysis used? |  |  |  |
| Overall appraisal (total number of questions is 9 because question 9 was excluded as it did not apply to the studies evaluated) | **High risk of bias:**  up to 49% “Yes” responses  **Moderate or unclear risk of bias:**  50%–69% “Yes” responses  **Low risk of bias:**  70% or more “Yes” | | |

Table S3: Joanna Briggs Institute critical appraisal checklist for cohort studies.

| **CHECKLIST** | Yes | No | Unclear |
| --- | --- | --- | --- |
| 1.Were the two groups similar and recruited from the same population? |  |  |  |
| 2.Were the exposures measured similarly to assign people to both exposed and unexposed groups? |  |  |  |
| 3.Was the exposure measured in a valid and reliable way? |  |  |  |
| 4.Were confounding factors identified? |  |  |  |
| 5.Were strategies to deal with confounding factors stated? |  |  |  |
| 7.Were the outcomes measured in a valid and reliable way? |  |  |  |
| 11.Was appropriate statistical analysis used? |  |  |  |
| Overall appraisal (total number of questions is 7 because questions 6, and 8 to 10 were excluded as they did not apply to the studies evaluated) | **High risk of bias:**  up to 49% “Yes” responses  **Moderate or unclear risk of bias:**  50%–69% “Yes” responses  **Low risk of bias:**  70% or more “Yes” | | |

.

Table S4: SYRCLE’s critical appraisal checklist for animal studies.

| **CHECKLIST** | Low | High | Unclear |
| --- | --- | --- | --- |
| 1.Was the allocation sequence adequately generated and applied? |  |  |  |
| 2.Were the groups similar at baseline or were they adjusted for confounders in the analysis? |  |  |  |
| 3.Was the allocation adequately concealed? |  |  |  |
| 4.Were the animals randomly housed during the experiment? |  |  |  |
| 5.Were the caregivers and /or investigators blinded from knowledge which intervention each animal received during the experiment? |  |  |  |
| 6.Were animals selected at random for outcome assessment? |  |  |  |
| 7.Was the outcome assessor blinded? |  |  |  |
| 8.Were incomplete outcome data adequately addressed? |  |  |  |
| 9.Are reports of the study free of selective outcome reporting? |  |  |  |
| 10.Was the study apparently free of other problems that could result in high risk of bias? |  |  |  |

**RESULTS**

**Study selection**

| Table S5: Articles excluded with the reasons after the full text evaluation. | |
| --- | --- |
| **Author/Year** | **Exclusion reasons** |
| Ahn et al. (2012) | PECO exclusion: cancer type not specified (only HNSCC/OSCC included) |
| Ahlstrand (2024) | Study protocol |
| Al-Hebshi et al. (2017) | PECO exclusion: cancer type not specified (only HNSCC/OSCC included) |
| Al-Nawas and Grötz (2006) | PECO exclusion: cancer type not specified (only HNSCC/OSCC included) |
| Al-Wesabi et al. (2021) | Preprint |
| Ali et al. (2022) | PECO exclusion: cancer type not specified (only HNSCC/OSCC included) |
| Anjali et al. (2023) | PECO exclusion criteria (no clinical periodontal data) |
| Aral et al. (2020) | PECO exclusion: cancer type not specified (only HNSCC/OSCC included) |
| Arduino et al. (2021) | PECO exclusion criteria (no clinical periodontal data) |
| Bachar et al. (2011) | PECO exclusion criteria (no clinical periodontal data) |
| Barbarisi et al. (2024) | PECO exclusion criteria (no clinical periodontal data) |
| Benito-Ramal et al. (2024) | PECO exclusion: cancer type not specified (only HNSCC/OSCC included) |
| Benjamin et al. (2023) | PECO exclusion criteria (no clinical periodontal data) |
| Bertl et al. (2016) | PECO exclusion: cancer type not specified (only HNSCC/OSCC included) |
| Bertl et al. (2022) | PECO exclusion: cancer type not specified (only HNSCC/OSCC included) |
| Bhat et al. (2019) | PECO exclusion: cancer type not specified (only HNSCC/OSCC included) |
| Bloching et al. (2007) | PECO exclusion: cancer type not specified (only HNSCC/OSCC included) |
| Bloching et al. (2008) | PECO exclusion: cancer type not specified (only HNSCC/OSCC included) |
| Boia et al. (2018) | Conference proceeding |
| Bolyarova-Konova et al. (2025) | PECO exclusion: OSCC and periodontitis were analyzed in separate groups |
| Bonan et al. (2006) | PECO exclusion: cancer type not specified (only HNSCC/OSCC included) |
| Brandt et al. (2023) | PECO exclusion: cancer type not specified (only HNSCC/OSCC included) |
| Brennan et al. (2022) | PECO exclusion: cancer type not specified (only HNSCC/OSCC included) |
| Brennan et al. (2021) | PECO exclusion: cancer type not specified (only HNSCC/OSCC included) |
| Bueno et al. (2013) | PECO exclusion: cancer type not specified (only HNSCC/OSCC included) |
| Bueno et al. (2015) | PECO exclusion: cancer type not specified (only HNSCC/OSCC included) |
| Bundugji et al. (2021) | PECO exclusion: cancer type not specified (only HNSCC/OSCC included) |
| Buurman et al. (2022) | PECO exclusion: cancer type not specified (only HNSCC/OSCC included) |
| Byakodi et al. (2011) | PECO exclusion: cancer type not specified (only HNSCC/OSCC included) |
| Castañeda-Corzo et al. (2023) | PECO exclusion criteria (no clinical periodontal data) |
| Chan et al. (2021) | PECO exclusion: cancer type not specified (only HNSCC/OSCC included) |
| Chellappa and Leelavathi (2020) | PECO exclusion: cancer type not specified (only HNSCC/OSCC included) |
| Chen et al. (2017) | PECO exclusion criteria (no clinical periodontal data) |
| Chen et al. (2018) | PECO exclusion criteria (no clinical periodontal data) |
| P. J. Chen et al. (2021) | PECO exclusion: cancer type not specified (only HNSCC/OSCC included) |
| Q. Chen et al. (2021) | PECO exclusion criteria (no clinical periodontal data) |
| J. W. Chen et al. (2021) | PECO exclusion criteria (no clinical periodontal data) |
| M. F. Chen et al. (2021) | PECO exclusion: cancer type not specified (only HNSCC/OSCC included) |
| Chen et al. (2022) | PECO exclusion criteria (no clinical periodontal data) |
| Cheng et al. (2017) | PECO exclusion criteria (no clinical periodontal data) |
| Cheng et al. (2014) | PECO exclusion criteria (no clinical periodontal data) |
| Chung et al. (2016) | PECO exclusion: cancer type not specified (only HNSCC/OSCC included) |
| Cochrane et al. (2020) | PECO exclusion: cancer type not specified (only HNSCC/OSCC included) |
| Contaldo et al. (2020) | Review |
| Conway (2009) | Letter to the Editor |
| Critchlow et al. (2014) | PECO exclusion: cancer type not specified (only HNSCC/OSCC included) |
| da Silva et al. (2024) | PECO exclusion: cancer type not specified (only HNSCC/OSCC included) |
| Daily et al. (2025) | PECO exclusion: cancer type not specified (only HNSCC/OSCC included) |
| Dar et al. (2013) | PECO exclusion: cancer type not specified (only HNSCC/OSCC included) |
| Dhingra (2022) | Review |
| Dholam et al. (2021) | PECO exclusion criteria (no clinical periodontal data) |
| Di Spirito et al. (2022) | Review |
| Dizdar et al. (2017) | PECO exclusion: cancer type not specified (only HNSCC/OSCC included) |
| Epstein et al. (1999) | PECO exclusion: cancer type not specified (only HNSCC/OSCC included) |
| Epstein et al. (1998) | PECO exclusion: cancer type not specified (only HNSCC/OSCC included) |
| Erira et al. (2021) | PECO exclusion criteria (no clinical periodontal data) |
| Erira et al. (2022) | Other language |
| Escoda-Francolí et al. (2011) | PECO exclusion: cancer type not specified (only HNSCC/OSCC included) |
| Eun et al. (2021) | PECO exclusion criteria (no clinical periodontal data) |
| Fitzsimonds et al. (2020) | Review |
| Fotedar et al. (2019) | PECO exclusion criteria (no clinical periodontal data) |
| Friemel et al. (2016) | PECO exclusion criteria (no clinical periodontal data) |
| Frydrych and Slack-Smith (2011) | PECO exclusion criteria (no clinical periodontal data) |
| Fu and Zheng (2023) | PECO exclusion criteria (no clinical periodontal data) |
| Gabusi et al. (2023) | PECO exclusion criteria (no clinical periodontal data) |
| Gaetti-Jardim et al. (2018) | PECO exclusion: cancer type not specified (only HNSCC/OSCC included) |
| Ganly et al. (2019) | PECO exclusion criteria (no clinical periodontal data) |
| Gao et al. (2015) | PECO exclusion: cancer type not specified (only HNSCC/OSCC included) |
| Garrote et al. (2001) | PECO exclusion: cancer type not specified (only HNSCC/OSCC included) |
| Ghanem et al. (2025) | PECO exclusion: cancer type not specified (only HNSCC/OSCC included) |
| Gopinath, Kunnath Menon, et al. (2021) | PECO exclusion criteria (no clinical periodontal data) |
| Gopinath, Menon, et al. (2021) | PECO exclusion criteria (no clinical periodontal data) |
| Guerrero-Preston et al. (2017) | PECO exclusion criteria (no clinical periodontal data) |
| Guha et al. (2007) | PECO exclusion criteria (no clinical periodontal data) |
| Guo et al. (2021) | PECO exclusion: cancer type not specified (only HNSCC/OSCC included) |
| Gupta and Johnson (2017) | Conference proceedings |
| Güven et al. (2019) | PECO exclusion: cancer type not specified (only HNSCC/OSCC included) |
| Han et al. (2014) | Review |
| Hashim et al. (2016) | PECO exclusion: cancer type not specified (only HNSCC/OSCC included) |
| Hashimoto et al. (2019) | PECO exclusion criteria (no clinical periodontal data) |
| Hashimoto et al. (2022) | PECO exclusion criteria (no clinical periodontal data) |
| Hasnat et al. (2025) | PECO exclusion (No periodontopathogens assessed) |
| Hayes et al. (2018) | PECO exclusion criteria (no clinical periodontal data) |
| Heikkilä et al. (2018) | PECO exclusion: cancer type not specified (only HNSCC/OSCC included) |
| Heng et al. (2022) | PECO exclusion criteria (no clinical periodontal data) |
| Heredia et al. (2017) | Other language |
| Herreros-Pomares et al. (2023) | PECO exclusion: cancer type not specified (only HNSCC/OSCC included) |
| Hosainzadegan et al. (2022) | PECO exclusion criteria (no clinical periodontal data) |
| Hsiao et al. (2018) | PECO exclusion criteria (no clinical periodontal data) |
| Iturbide et al. (2017) | Conference proceedings |
| Jácome-Santos et al. (2022) | PECO exclusion: cancer type not specified (only HNSCC/OSCC included) |
| Jebril et al. (2024) | PECO exclusion: cancer type not specified (only HNSCC/OSCC included) |
| John et al. (2019) | PECO exclusion: cancer type not specified (only HNSCC/OSCC included) |
| Jouhi et al. (2022) | PECO exclusion: cancer type not specified (only HNSCC/OSCC included) |
| Kaliamoorthy et al. (2021) | PECO exclusion criteria (no clinical periodontal data) |
| Kamarajan et al. (2016) | Conference proceedings |
| Kang et al. (2009) | PECO exclusion criteria (no clinical periodontal data) |
| Karri et al. (2024) | PECO exclusion: OSCC and periodontitis were analyzed in separate groups |
| Kasimov et al. (2021) | Other language |
| Katz et al. (2011) | PECO exclusion criteria (no clinical periodontal data) |
| Keskin et al. (2020) | PECO exclusion: cancer type not specified (only HNSCC/OSCC included) |
| Khan et al. (2021) | Review |
| Kim et al. (2022) | PECO exclusion: cancer type not specified (only HNSCC/OSCC included) |
| Kim et al. (2025) | PECO exclusion: cancer type not specified (only HNSCC/OSCC included) |
| Kindler et al. (2021) | PECO exclusion: cancer type not specified (only HNSCC/OSCC included) |
| Kioi et al. (2023) | Conference proceeding |
| Ko et al. (2025) | PECO exclusion criteria (no clinical periodontal data) |
| Kozłowski et al. (2009) | Other language |
| Krüger et al. (2013) | PECO exclusion criteria (study design) |
| Kylmä et al. (2018) | PECO exclusion criteria (no clinical periodontal data) |
| Kylmae et al. (2022) | PECO exclusion criteria (no clinical periodontal data) |
| Laheij et al. (2013) | PECO exclusion: cancer type not specified (only HNSCC/OSCC included) |
| Laliberte et al. (2021) | PECO exclusion: cancer type not specified (only HNSCC/OSCC included) |
| Lalla et al. (2017) | Study protocol |
| Lenz et al. (2005) | PECO exclusion criteria (no clinical periodontal data) |
| Li et al. (2018) | PECO exclusion criteria (no clinical periodontal data) |
| Li et al. (2020) | PECO exclusion: cancer type not specified (only HNSCC/OSCC included) |
| Li et al. (2023) | PECO exclusion: cancer type not specified (only HNSCC/OSCC included) |
| Li et al. (2021) | PECO exclusion: cancer type not specified (only HNSCC/OSCC included) |
| Lim et al. (2018) | PECO exclusion: cancer type not specified (only HNSCC/OSCC included) |
| Lin et al. (2015) | PECO exclusion: cancer type not specified (only HNSCC/OSCC included) |
| Listyarifah et al. (2018) | PECO exclusion criteria (no clinical periodontal data) |
| Liu et al. (2025) | Other language |
| Lockhart and Clark (1994) | PECO exclusion: cancer type not specified (only HNSCC/OSCC included) |
| Longo et al. (2023) | PECO exclusion: cancer type not specified (only HNSCC/OSCC included) |
| Lyu et al. (2023) | Conference proceedings |
| Mager et al. (2005) | PECO exclusion: cancer type not specified (only HNSCC/OSCC included) |
| Magrin et al. (2020) | PECO exclusion criteria (no clinical periodontal data) |
| Mäkinen et al. (2023) | PECO exclusion criteria (no clinical periodontal data) |
| Marinescu-Gava et al. (2019) | Conference proceedings |
| Mauceri et al. (2023) | PECO exclusion: cancer type not specified (only HNSCC/OSCC included) |
| Medeiros et al. (2023) | PECO exclusion: cancer type not specified (only HNSCC/OSCC included) |
| Meng et al. (2020) | PECO exclusion criteria (no clinical periodontal data) |
| Meyle et al. (2022) | Conference proceedings |
| Michaud et al. (2008) | PECO exclusion: cancer type not specified (only HNSCC/OSCC included) |
| Michaud et al. (2017) | Review |
| Mok et al. (2017) | PECO exclusion: cancer type not specified (only HNSCC/OSCC included) |
| Monier et al. (2020) | PECO exclusion criteria (no clinical periodontal data) |
| Moreno-López et al. (2000) | PECO exclusion criteria (no clinical periodontal data) |
| Na et al. (2013) | PECO exclusion criteria (no clinical periodontal data) |
| Naavaal et al. (2022) | PECO exclusion: cancer type not specified (only HNSCC/OSCC included) |
| Nagy et al. (1998) | PECO exclusion: cancer type not specified (only HNSCC/OSCC included) |
| Nie et al. (2022) | PECO exclusion: cancer type not specified (only HNSCC/OSCC included) |
| Nieminen et al. (2018) | PECO exclusion: cancer type not specified (only HNSCC/OSCC included) |
| Nierengarten (2024) | Brief communication |
| Nikakhlagh et al. (2012) | PECO exclusion criteria (no clinical periodontal data) |
| Nitescu et al. (2017) | PECO exclusion: cancer type not specified (only HNSCC/OSCC included) |
| Nuñez-Aguilar et al. (2018) | PECO exclusion: cancer type not specified (only HNSCC/OSCC included) |
| Oballe et al. (2018) | PECO exclusion criteria (no clinical periodontal data) |
| Ohshima et al. (2019) | PECO exclusion: cancer type not specified (only HNSCC/OSCC included) |
| Panda et al. (2020) | PECO exclusion: cancer type not specified (only HNSCC/OSCC included) |
| Park et al. (2019) | PECO exclusion criteria (no clinical periodontal data) |
| Perera et al. (2018) | Letter to the Editor |
| Pranata et al. (2020) | PECO exclusion: cancer type not specified (only HNSCC/OSCC included) |
| Pushalkar et al. (2012) | PECO exclusion: cancer type not specified (only HNSCC/OSCC included) |
| Rai et al. (2021) | PECO exclusion: cancer type not specified (only HNSCC/OSCC included) |
| Rani et al. (2023) | PECO exclusion: cancer type not specified (only HNSCC/OSCC included) |
| Rodrigues et al. (2023) | PECO exclusion: cancer type not specified (only HNSCC/OSCC included) |
| Rouers et al. (2016) | Other language |
| Rupe et al. (2022) | PECO exclusion: cancer type not specified (only HNSCC/OSCC included) |
| Sahni (2023) | Review |
| Sarkar et al. (2021) | PECO exclusion: cancer type not specified (only HNSCC/OSCC included) |
| Sawant et al. (2021) | PECO exclusion: cancer type not specified (only HNSCC/OSCC included) |
| Saxena et al. (2022) | PECO exclusion: cancer type not specified (only HNSCC/OSCC included) |
| Schmidt et al. (2014) | PECO exclusion: cancer type not specified (only HNSCC/OSCC included) |
| Shankarram et al. (2015) | PECO exclusion: cancer type not specified (only HNSCC/OSCC included) |
| Shay et al. (2020) | PECO exclusion: cancer type not specified (only HNSCC/OSCC included) |
| Shin et al. (2017) | PECO exclusion criteria (no clinical periodontal data) |
| Silver et al. (2023) | Conference proceedings |
| Singh et al. (2019) | PECO exclusion: cancer type not specified (only HNSCC/OSCC included) |
| Singh et al. (2023) | PECO exclusion: cancer type not specified (only HNSCC/OSCC included) |
| Sobczynska-Rak et al. (2018) | PECO exclusion: cancer type not specified (only HNSCC/OSCC included) |
| Stashenko et al. (2019) | PECO exclusion: cancer type not specified (only HNSCC/OSCC included) |
| Su et al. (2021) | PECO exclusion: cancer type not specified (only HNSCC/OSCC included) |
| Talamini et al. (2000) | Brief communication |
| Tezal et al. (2005) | PECO exclusion: cancer type not specified (only HNSCC/OSCC included) |
| Ueda et al. (2021) | PECO exclusion: cancer type not specified (only HNSCC/OSCC included) |
| Vesty et al. (2018) | PECO exclusion criteria (no clinical periodontal data) |
| Vozza et al. (2015) | PECO exclusion: cancer type not specified (only HNSCC/OSCC included) |
| Wen et al. (2014) | PECO exclusion: cancer type not specified (only HNSCC/OSCC included) |
| Wu et al. (2023) | PECO exclusion: cancer type not specified (only HNSCC/OSCC included) |
| Xiao et al. (2025) | PECO exclusion: cancer type not specified (only HNSCC/OSCC included) |
| Xiong et al. (2024) | PECO exclusion: cancer type not specified (only HNSCC/OSCC included) |
| Yan et al. (2023) | PECO exclusion: cancer type not specified (only HNSCC/OSCC included) |
| C. Y. Yang et al. (2018) | PECO exclusion criteria (no clinical periodontal data) |
| Yang et al. (2022) | PECO exclusion: cancer type not specified (only HNSCC/OSCC included) |
| Yang et al. (2021) | PECO exclusion: cancer type not specified (only HNSCC/OSCC included) |
| S. F. Yang et al. (2018) | PECO exclusion: cancer type not specified (only HNSCC/OSCC included) |
| Yang et al. (2023) | PECO exclusion: cancer type not specified (only HNSCC/OSCC included) |
| Ye et al. (2021) | PECO exclusion: cancer type not specified (only HNSCC/OSCC included) |
| Yost et al. (2018) | PECO exclusion: cancer type not specified (only HNSCC/OSCC included) |
| Zhang et al. (2020) | PECO exclusion: cancer type not specified (only HNSCC/OSCC included) |
| Zhang et al. (2022) | PECO exclusion criteria (no clinical periodontal data) |
| Zhang et al. (2025) | PECO exclusion criteria (no clinical periodontal data) |
| Zhao et al. (2017) | PECO exclusion: cancer type not specified (only HNSCC/OSCC included) |
| Zheng et al. (1990) | PECO exclusion criteria (no clinical periodontal data) |
| Zhong et al. (2021) | PECO exclusion: cancer type not specified (only HNSCC/OSCC included) |
| Zhong et al. (2020) | PECO exclusion criteria (no clinical periodontal data) |
| Zhou et al. (2020) | PECO exclusion: cancer type not specified (only HNSCC/OSCC included) |

76

**
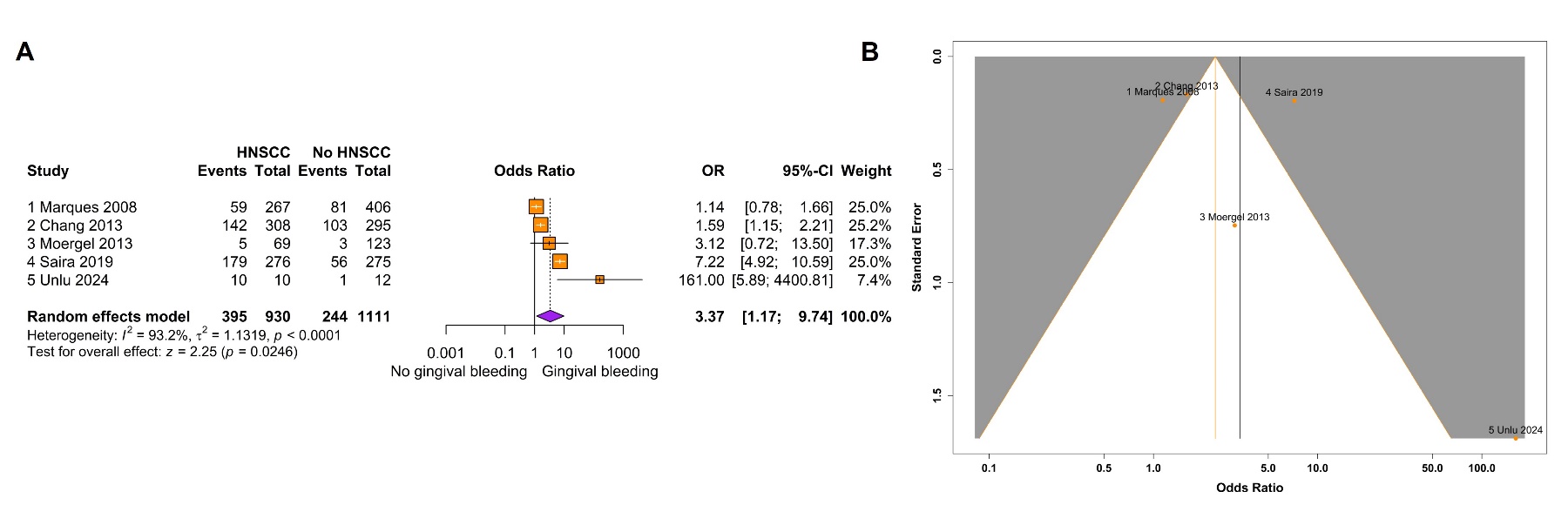
Meta-analysis results**

Figure S1: Head and neck squamous cell carcinoma (HNSCC) occurrence based on self-reported gingival bleeding by questionnaire. A. Forest plot. B: Funnel plot.


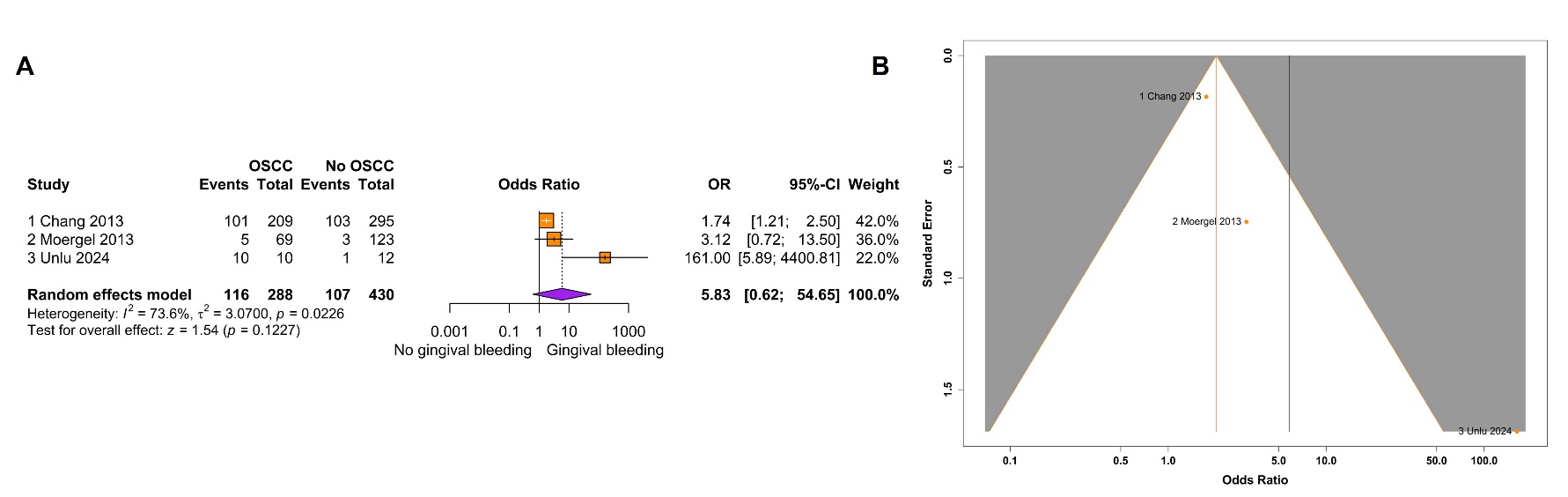


Figure S2: Oral squamous cell carcinoma (OSCC) occurrence based on self-reported gingival bleeding by questionnaire. A. Forest plot. B: Funnel plot.


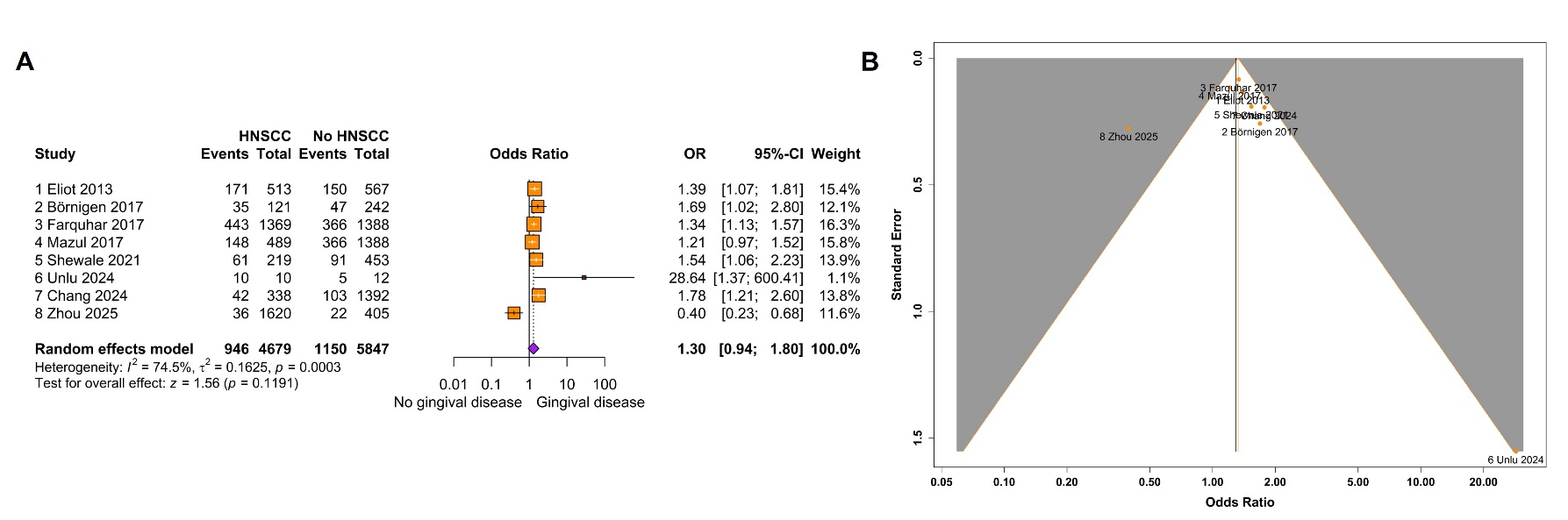
Figure S3: Head and neck squamous cell carcinoma (HNSCC) occurrence based on self-reported gum disease by questionnaire. A. Forest plot. B: Funnel plot.


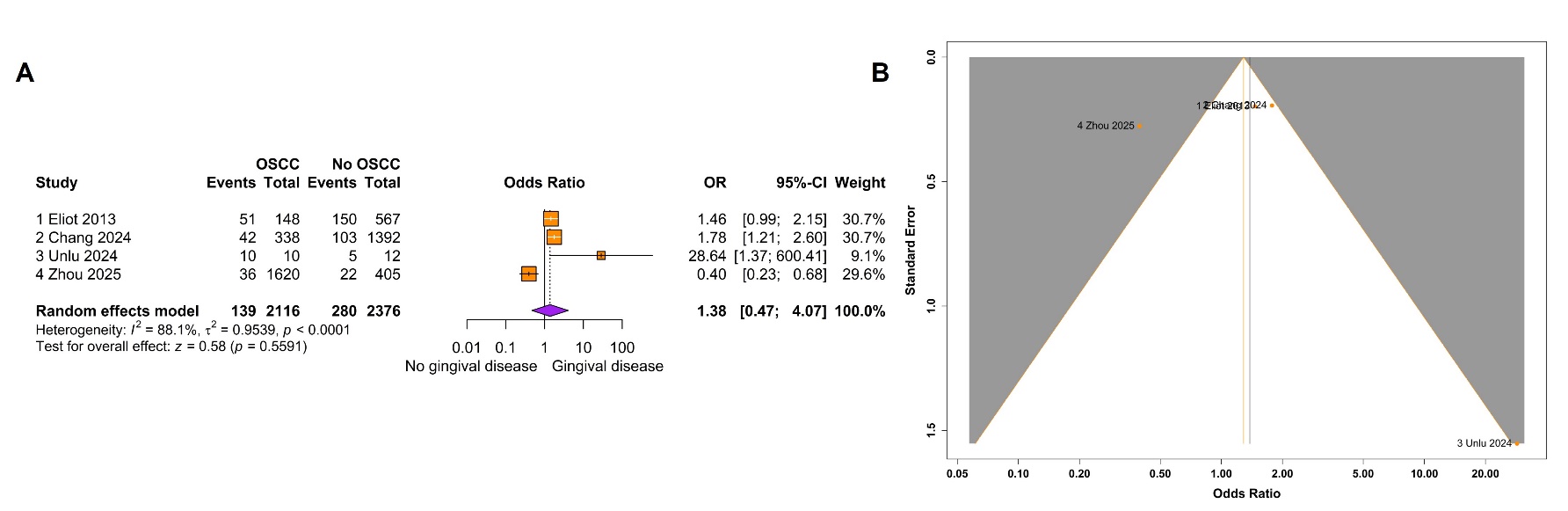


Figure S4: Oral squamous cell carcinoma (OSCC) occurrence based on self-reported gum disease by questionnaire. A. Forest plot. B: Funnel plot.


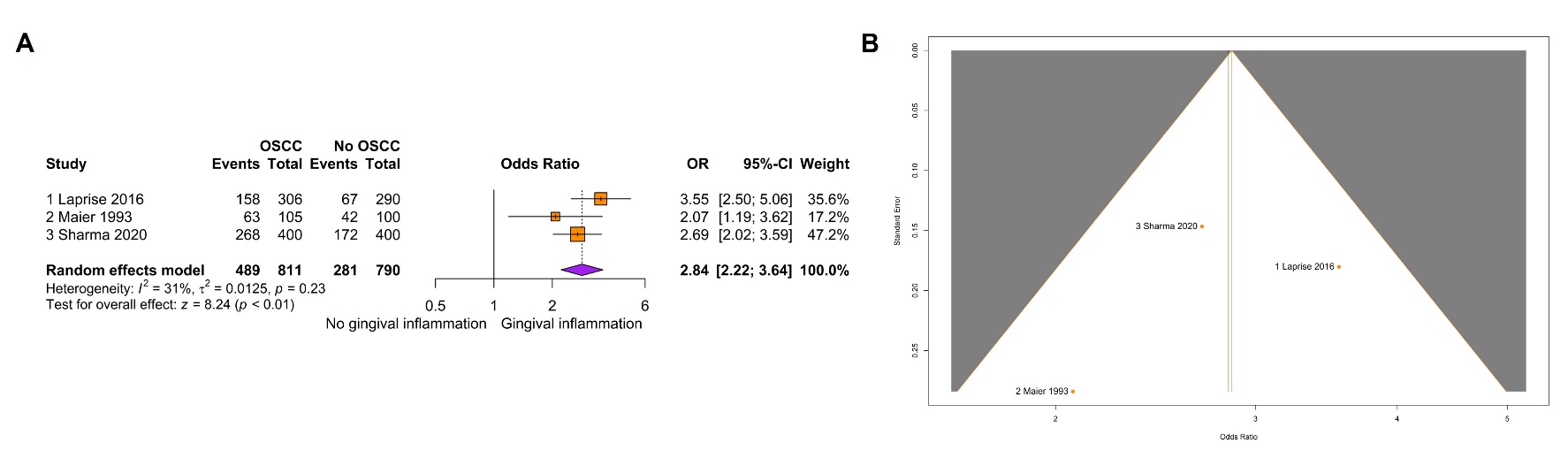


Figure S5: Oral squamous cell carcinoma (OSCC) occurrence based on clinical diagnosis of gingival inflammation. A. Forest plot. B: Funnel plot.


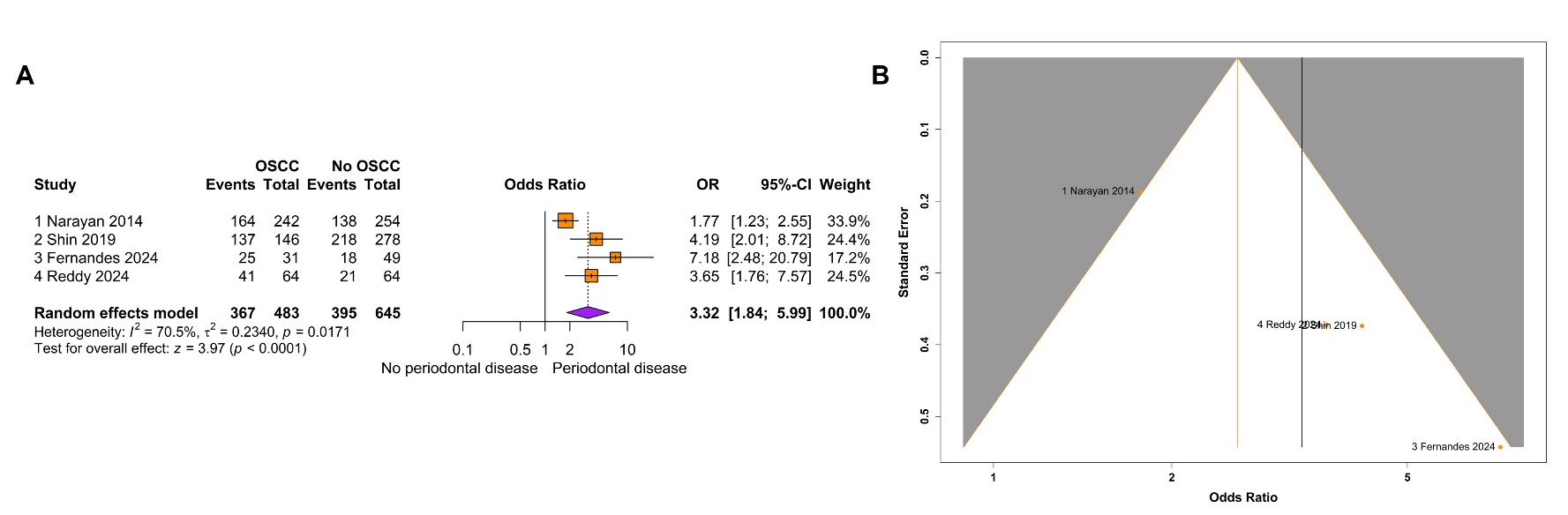


Figure S6: Oral squamous cell carcinoma (OSCC) occurrence based on clinical diagnosis of periodontal disease. A. Forest plot. B: Funnel plot.


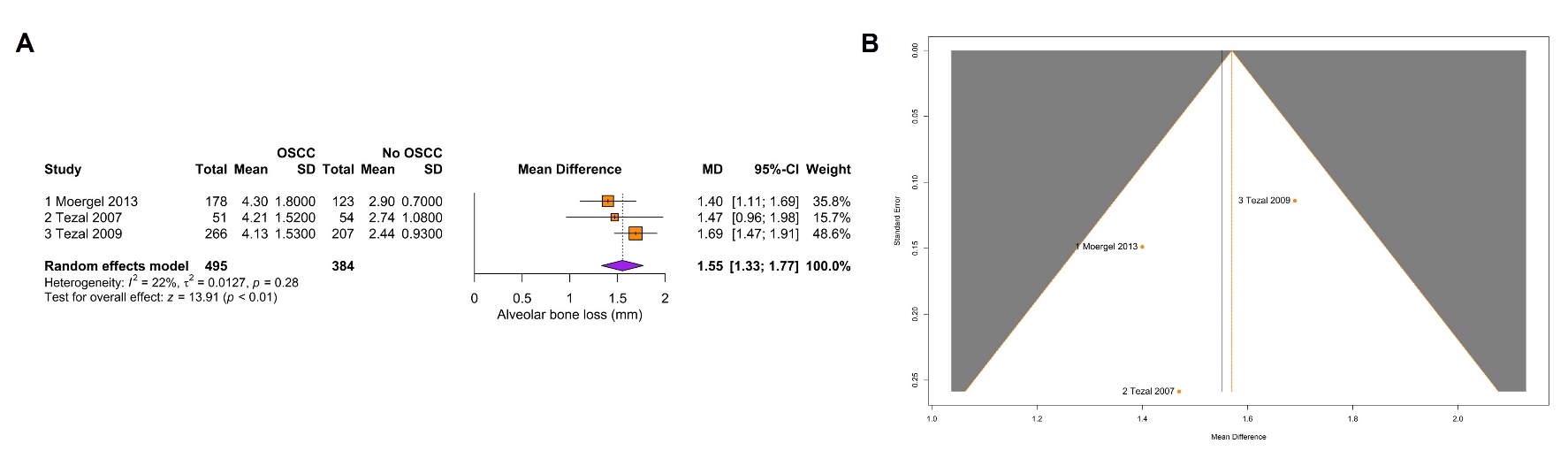


Figure S7: Oral squamous carcinoma cells (OSCC) occurrence based on alveolar bone loss (mm). A. Forest plot. B: Funnel plot.


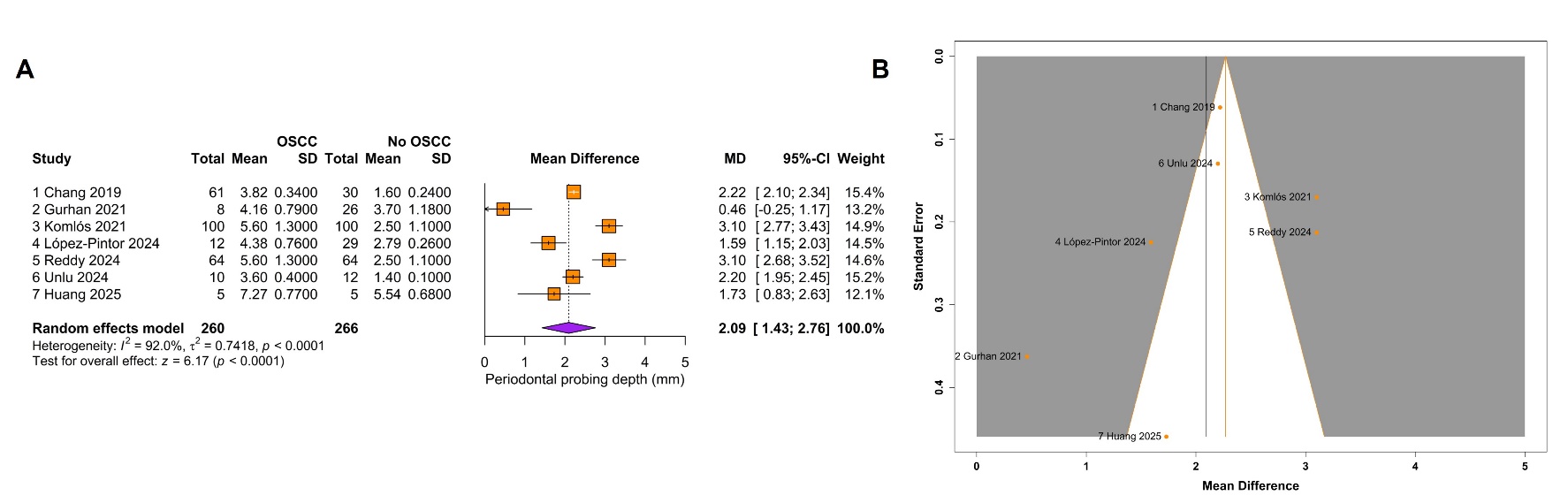


Figure S8: Oral squamous cell carcinoma (OSCC) occurrence based on periodontal probing depth (mm). A. Forest plot. B: Funnel plot.

**
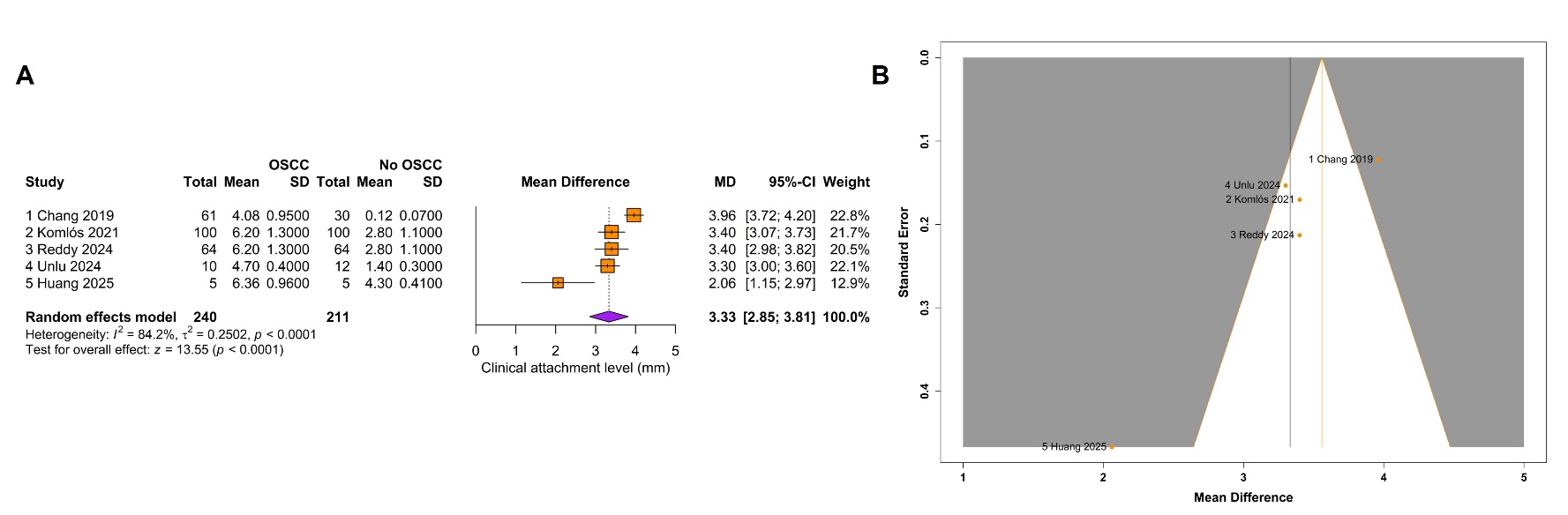
**

Figure S9: Oral squamous cell carcinoma (OSCC) occurrence based on clinical attachment level (mm). A. Forest plot. B: Funnel plot.


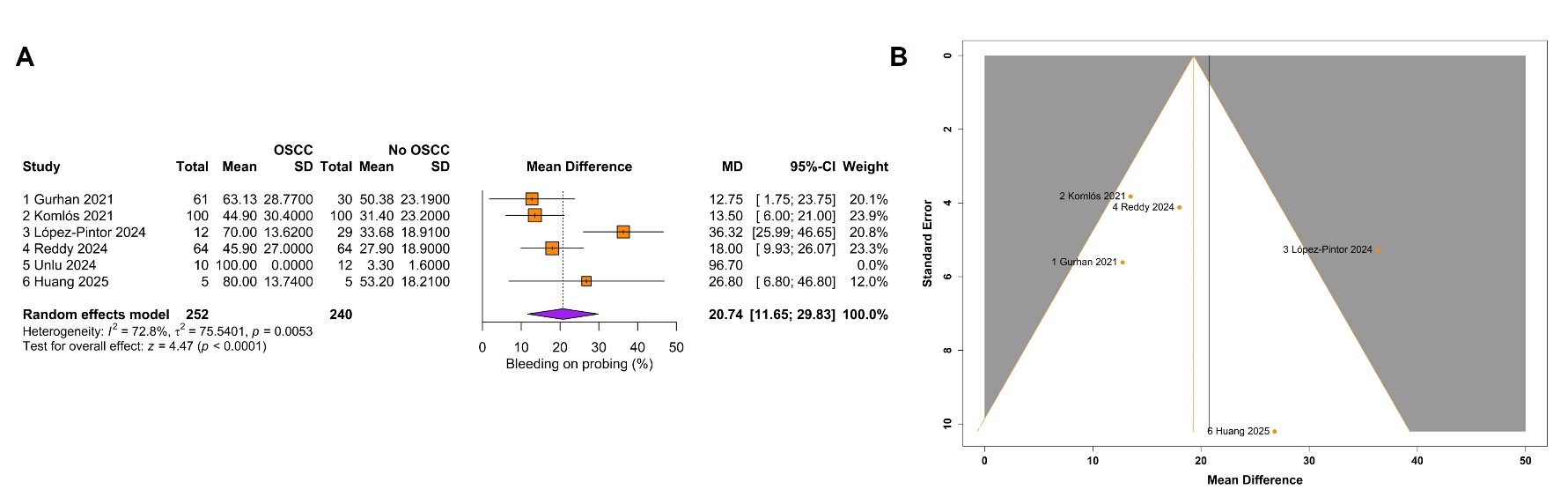


Figure S10: Oral squamous cell carcinoma (OSCC) occurrence based on bleeding on probing (%). A. Forest plot. B: Funnel plot.

**REFERENCES**

Ahlstrand, S. (2024). Active MMP-8 in Oral Fluid for the Detection of Oral and Oropharyngeal Cancer. In.

Ahn, J., Segers, S., & Hayes, R. B. (2012). Periodontal disease, Porphyromonas gingivalis serum antibody levels and orodigestive cancer mortality. *CARCINOGENESIS*, *33*(5), 1055-1058. <https://doi.org/10.1093/carcin/bgs112>

Al-Hebshi, N. N., Nasher, A. T., Maryoud, M. Y., Homeida, H. E., Chen, T., Idris, A. M., & Johnson, N. W. (2017). Inflammatory bacteriome featuring Fusobacterium nucleatum and Pseudomonas aeruginosa identified in association with oral squamous cell carcinoma. *Sci Rep*, *7*(1), 1834. <https://doi.org/10.1038/s41598-017-02079-3>

Al-Nawas, B., & Grötz, K. A. (2006). Prospective study of the long term change of the oral flora after radiation therapy [Article]. *SUPPORTIVE CARE IN CANCER*, *14*(3), 291-296. <https://doi.org/10.1007/s00520-005-0895-3>

Al-Wesabi, M. A., Al –Matari, S. M., & Al-Jawfi, K. A. (2021). Histopathological Findings of Oral and Maxillofacial Biopsies from a Sample of Yemeni Patients [Article]. *Journal of International Dental and Medical Research*, *14*(1), 235-241. <https://www.scopus.com/inward/record.uri?eid=2-s2.0-85104731702&partnerID=40&md5=d009227ff81f47e7bd9976bb41f46d71>

Ali, A., Rumbold, A. R., Kapellas, K., Lassi, Z. S., Hedges, J., & Jamieson, L. (2022). Association between self-reported periodontitis and high-risk oral human papillomavirus infection among Indigenous South Australians: A cross-sectional study [Article]. *PLOS ONE*, *17*(3 March). <https://doi.org/10.1371/journal.pone.0265840>

Anjali, K., Manzoor, M., Suryavanshi, M. V., Rudrapathy, P., Rekha, P. D., Das, R., Hameed, A., & Arun, A. B. (2023). Dysbiosis of the oral microbiota composition is associated with oral squamous cell carcinoma and the impact of radiotherapy: a pilot study. *FEMS Microbiology Letters*, *370*. <https://doi.org/10.1093/femsle/fnad111>

Aral, C. A., Ölçer, S. N., Aral, K., & Kapila, Y. (2020). Oxidative stress, neutrophil elastase and IGFBP7 levels in patients with oropharyngeal cancer and chronic periodontitis. *ORAL DISEASES*, *26*(7), 1393-1401. <https://doi.org/10.1111/odi.13370>

Arduino, P. G., Carbone, M., Gambino, A., Cabras, M., Cannarsa, F., Macciotta, A., Conrotto, D., & Broccoletti, R. (2021). Challenging management of gingival squamous cell carcinoma: A 10 years single-center retrospective study on Northern-Italian patients [Article]. *MEDICINA ORAL PATOLOGIA ORAL Y CIRUGIA BUCAL*, *26*(1), e21-e27. <https://doi.org/10.4317/medoral.23913>

Bachar, G., Hod, R., Goldstein, D. P., Irish, J. C., Gullane, P. J., Brown, D., Gilbert, R. W., Hadar, T., Feinmesser, R., & Shpitzer, T. (2011). Outcome of oral tongue squamous cell carcinoma in patients with and without known risk factors [Article]. *ORAL ONCOLOGY*, *47*(1), 45-50. <https://doi.org/10.1016/j.oraloncology.2010.11.003>

Barbarisi, A., Cremonini, F., Lauritano, D., Visconti, V., Caccianiga, G., & Ceraulo, S. (2024). Association between Periodontal Disease and Oral Benign, Potentially Malignant, Malignant, and Chronic Immune-Mediated Disorders: A Clinical Study [Article]. *Healthcare (Switzerland)*, *12*(19), Article 1999. <https://doi.org/10.3390/healthcare12191999>

Benito-Ramal, E., Camacho-Mourelo, A., González-Navarro, B., López, J. L., & Jané-Salas, E. (2024). Prevalence and risk factors of chronic oral complications in head and neck cancer therapies: A retrospective study [Article]. *Medicina Oral Patologia Oral y Cirugia Bucal*, *29*(6), e850-e856. <https://doi.org/10.4317/medoral.26823>

Benjamin, W. J., Wang, K., Zarins, K., Bellile, E., Blostein, F., Argirion, I., Taylor, J. M. G., D'Silva, N. J., Chinn, S. B., Rifkin, S., Sartor, M. A., & Rozek, L. S. (2023). Oral Microbiome Community Composition in Head and Neck Squamous Cell Carcinoma. *CANCERS*, *15*(9), Article 2549. <https://doi.org/10.3390/cancers15092549>

Bertl, K., Loidl, S., Kotowski, U., Heiduschka, G., Thurnher, D., Stavropoulos, A., & Schneider-Stickler, B. (2016). Oral health status and dental care behaviours of head and neck cancer patients: a cross-sectional study in an Austrian tertiary hospital. *CLINICAL ORAL INVESTIGATIONS*, *20*(6), 1317-1327. <https://doi.org/10.1007/s00784-015-1618-x>

Bertl, K., Savvidis, P., Kukla, E. B., Schneider, S., Zauza, K., Bruckmann, C., & Stavropoulos, A. (2022). Including dental professionals in the multidisciplinary treatment team of head and neck cancer patients improves long-term oral health status. *CLINICAL ORAL INVESTIGATIONS*, *26*(3), 2937-2948. <https://doi.org/10.1007/s00784-021-04276-x>

Bhat, M., Bhat, S., Roberts-Thomson, K., & Do, L. G. (2019). Is Periodontitis Independently Associated with Potentially Malignant Disorders of the Oral Cavity? *Asian Pac J Cancer Prev*, *20*(10), 283-287. <https://doi.org/10.31557/APJCP.2019.20.1.283>

Bloching, M., Reich, W., Schubert, J., Grummt, T., & Sandner, A. (2007). The influence of oral hygiene on salivary quality in the Ames Test, as a marker for genotoxic effects [Article]. *ORAL ONCOLOGY*, *43*(9), 933-939. <https://doi.org/10.1016/j.oraloncology.2006.11.006>

Bloching, M., Reich, W., Schubert, J., Grummt, T., & Sandner, A. (2008). Micronucleus rate of buccal mucosal epithelial cells in relation to oral hygiene and dental factors [Article]. *ORAL ONCOLOGY*, *44*(3), 220-226. <https://doi.org/10.1016/j.oraloncology.2007.02.002>

Boia, S., Ursoniu, S., Boia, E. R., & Borza, C. (2018). *Oxidative Stress Evaluation in Patients with Periodontal Disease and Oral Squamous Cell Carcinoma* PROCEEDINGS OF NATIONAL ENT, HEAD AND NECK SURGERY CONFERENCE,

Bolyarova-Konova, T., Mihailova, Z., Adamov, A., Stanimirov, P., Dzhenkov, S., Stamatov, K., & Aleksiev, E. (2025). CLINICAL DATA ANALYSIS ON THE ASSOCIATION BETWEEN PERIODONTAL DISEASE, THE DEVELOPMENT OF ORAL SQUAMOUS CELL CARCINOMA AND ORAL POTENTIALLY MALIG NANT DISORDERS [Article]. *Journal of IMAB - Annual Proceeding (Scientific Papers)*, *31*(2), 6153-6158. <https://doi.org/10.5272/jimab.2025312.6153>

Bonan, P. R. F., Lopes, M. A., Pires, F. R., & de Almeida, O. P. (2006). Dental management of low socioeconomic level patients before radiotherapy of the head and neck with special emphasis on the prevention of osteoradionecrosis [Article]. *Brazilian Dental Journal*, *17*(4), 336-342. <https://doi.org/10.1590/s0103-64402006000400013>

Brandt, E., Keskin, M., Tervahartiala, T., Yilmaz, M., Harmankaya, I., Karaçetin, D., Ipek, T., Gürsoy, U. K., Rautava, J., Gupta, S., Hagström, J., Räisänen, I. T., & Sorsa, T. (2023). Radiotherapy Increases aMMP-8-Levels and Neutrophil/Lymphocyte Ratio Rapidly in Head and Neck Cancer Patients: A Pilot Study. *CANCER CONTROL*, *30*, Article 10732748231163653. <https://doi.org/10.1177/10732748231163653>

Brennan, M. T., Treister, N. S., Sollecito, T. P., Schmidt, B. L., Patton, L. L., Lin, A., Elting, L. S., Hodges, J. S., & Lalla, R. V. (2022). Tooth Failure Post-Radiotherapy in Head and Neck Cancer: Primary Report of the Clinical Registry of Dental Outcomes in Head and Neck Cancer Patients (OraRad) Study [Article]. *International Journal of Radiation Oncology Biology Physics*, *113*(2), 320-330. <https://doi.org/10.1016/j.ijrobp.2021.11.021>

Brennan, M. T., Treister, N. S., Sollecito, T. P., Schmidt, B. L., Patton, L. L., Yang, Y., Lin, A., Elting, L. S., Hodges, J. S., & Lalla, R. V. (2021). Epidemiologic factors in patients with advanced head and neck cancer treated with radiation therapy [Article]. *Head and Neck*, *43*(1), 164-172. <https://doi.org/10.1002/hed.26468>

Bueno, A. C., Ferreira, R. C., Barbosa, F. I., Jham, B. C., Magalhães, C. S., & Moreira, A. N. (2013). Periodontal care in patients undergoing radiotherapy for head and neck cancer [Article]. *SUPPORTIVE CARE IN CANCER*, *21*(4), 969-975. <https://doi.org/10.1007/s00520-012-1614-5>

Bueno, A. C., Ferreira, R. C., Cota, L. O. M., Silva, G. C., Magalhães, C. S., & Moreira, A. N. (2015). Comparison of different criteria for periodontitis case definition in head and neck cancer individuals [Article]. *SUPPORTIVE CARE IN CANCER*, *23*(9), 2599-2604. <https://doi.org/10.1007/s00520-015-2618-8>

Bundugji, M. H., Bafarat, D., Bundagji, N., Arafsha, Y., & Hassan, H. (2021). Assessment of oral health and care in head and neck oncology patients in King Faisal Specialist Hospital and Research Center-Jeddah [Article]. *SAUDI DENTAL JOURNAL*, *33*(8), 1174-1183. <https://doi.org/10.1016/j.sdentj.2021.01.004>

Buurman, D. J. M., Willemsen, A. C. H., Speksnijder, C. M., Baijens, L. W. J., Hoeben, A., Hoebers, F. J. P., Kessler, P., & Schols, A. (2022). Tooth extractions prior to chemoradiation or bioradiation are associated with weight loss during treatment for locally advanced oropharyngeal cancer. *SUPPORTIVE CARE IN CANCER*, *30*(6), 5329-5338. <https://doi.org/10.1007/s00520-022-06942-6>

Byakodi, R., Krishnappa, R., Keluskar, V., Bagewadi, A., & Shetti, A. (2011). The microbial flora associated with oral carcinomas [Article]. *QUINTESSENCE INTERNATIONAL*, *42*(9), e118-e123. <https://www.scopus.com/inward/record.uri?eid=2-s2.0-84857406088&partnerID=40&md5=b639382d682c44e0b94035e2a99d1296>

Castañeda-Corzo, G. J., Infante-Rodríguez, L. F., Villamil-Poveda, J. C., Bustillo, J., Cid-Arregui, A., & García-Robayo, D. A. (2023). Association of<i> Prevotella</i><i> intermedia</i> with oropharyngeal cancer: A patient-control study. *HELIYON*, *9*(3), Article e14293. <https://doi.org/10.1016/j.heliyon.2023.e14293>

Chan, J. Y. K., Ng, C. W. K., Lan, L., Fung, S., Li, J. W., Cai, L., Lei, P., Mou, Q., Meehan, K., Lau, E. H. L., Yeung, Z., Chan, K. C. A., Wong, E. W. Y., Chan, P. K. S., & Chen, Z. (2021). Restoration of the Oral Microbiota After Surgery for Head and Neck Squamous Cell Carcinoma Is Associated With Patient Outcomes [Article]. *FRONTIERS IN ONCOLOGY*, *11*, Article 737843. <https://doi.org/10.3389/fonc.2021.737843>

Chellappa, L. R., & Leelavathi, L. (2020). Association of systemic diseases with periodontitis among patients attending a private dental college in Chennai-a record based study [Article]. *Indian Journal of Forensic Medicine and Toxicology*, *14*(4), 4746-4754. <https://doi.org/10.37506/ijfmt.v14i4.12384>

Chen, F., He, B. C., Yan, L. J., Qiu, Y., Lin, L. S., & Cai, L. (2017). Influence of oral hygiene and its interaction with standard of education on the risk of oral cancer in women who neither smoked nor drank alcohol: a hospital-based, case-control study [Article]. *British Journal of Oral and Maxillofacial Surgery*, *55*(3), 260-265. <https://doi.org/10.1016/j.bjoms.2016.11.316>

Chen, F., Lin, L., Yan, L., Liu, F., Qiu, Y., Wang, J., Hu, Z., Wu, J., Bao, X., Lin, L., Wang, R., Cai, G., Aoyagi, K., Cai, L., & He, B. (2018). Nomograms and risk scores for predicting the risk of oral cancer in different sexes: A large-scale case-control study [Article]. *JOURNAL OF CANCER*, *9*(14), 2543-2548. <https://doi.org/10.7150/jca.24431>

Chen, J. W., Wu, J. H., Chiang, W. F., Chen, Y. L., Wu, W. S., & Wu, L. W. (2021). Taxonomic and Functional Dysregulation in Salivary Microbiomes During Oral Carcinogenesis [Article]. *FRONTIERS IN CELLULAR AND INFECTION MICROBIOLOGY*, *11*, Article 663068. <https://doi.org/10.3389/fcimb.2021.663068>

Chen, M. F., Lu, M. S., Hsieh, C. C., & Chen, W. C. (2021). <i>Porphyromonas gingivalis</i> promotes tumor progression in esophageal squamous cell carcinoma. *CELLULAR ONCOLOGY*, *44*(2), 373-384. <https://doi.org/10.1007/s13402-020-00573-x>

Chen, P. J., Chen, Y. Y., Lin, C. W., Yeh, Y. T., Yeh, H. W., Huang, J. Y., Yang, S. F., & Yeh, C. B. (2021). Effect of Periodontitis and Scaling and Root Planing on Risk of Pharyngeal Cancer: A Nested Case-Control Study. *INTERNATIONAL JOURNAL OF ENVIRONMENTAL RESEARCH AND PUBLIC HEALTH*, *18*(1), Article 8. <https://doi.org/10.3390/ijerph18010008>

Chen, Q., Shao, Z., Liu, K., Zhou, X., Wang, L., Jiang, E., Luo, T., & Shang, Z. (2021). Salivary Porphyromonas gingivalis predicts outcome in oral squamous cell carcinomas: a cohort study. *BMC ORAL HEALTH*, *21*(1), 228. <https://doi.org/10.1186/s12903-021-01580-6>

Chen, X., Lei, H., Cheng, Y. X., Fang, S. S., Sun, W. F., Zhang, X. C., & Jin, Z. L. (2022). CXCL8, MMP12, and MMP13 are common biomarkers of periodontitis and oral squamous cell carcinoma. *ORAL DISEASES*. <https://doi.org/10.1111/odi.14419>

Cheng, Y. L., Jordan, L., Chen, H. S., Kang, D., Oxford, L., Plemons, J., Parks, H., & Rees, T. (2017). Chronic periodontitis can affect the levels of potential oral cancer salivary mRNA biomarkers. *J Periodontal Res*, *52*(3), 428-437. <https://doi.org/10.1111/jre.12407>

Cheng, Y. S. L., Jordan, L., Gorugantula, L. M., Schneiderman, E., Chen, H. S., & Rees, T. (2014). Salivary Interleukin-6 and-8 in Patients With Oral Cancer and Patients With Chronic Oral Inflammatory Diseases. *JOURNAL OF PERIODONTOLOGY*, *85*(7), 956-965. <https://doi.org/10.1902/jop.2013.130320>

Chung, S. D., Tsai, M. C., Huang, C. C., Kao, L. T., & Chen, C. H. (2016). A population-based study on the associations between chronic periodontitis and the risk of cancer. *Int J Clin Oncol*, *21*(2), 219-223. <https://doi.org/10.1007/s10147-015-0884-6>

Cochrane, K., Podar, M., Carlson, E., Yang, Z., & Nodit, L. (2020). Oral microbiome changes associated with oral squamous cell carcinoma and salivary gland tumors [Conference Abstract]. *Modern Pathology*, *33*(3), 1185-1186. <https://www.embase.com/search/results?subaction=viewrecord&id=L631878362&from=export>

Contaldo, M., Boccellino, M., Zannini, G., Romano, A., Sciarra, A., Sacco, A., Settembre, G., Coppola, M., Di Carlo, A., D'Angelo, L., Inchingolo, F., Feola, A., & Di Domenico, M. (2020). Sex Hormones and Inflammation Role in Oral Cancer Progression: A Molecular and Biological Point of View. *JOURNAL OF ONCOLOGY*, *2020*, Article 9587971. <https://doi.org/10.1155/2020/9587971>

Conway, D. (2009). Oral health, mouthwashes and cancer--what is the story? *Evid Based Dent*, *10*(1), 6-7. <https://doi.org/10.1038/sj.ebd.6400624>

Critchlow, S. B., Morgan, C., & Leung, T. (2014). The oral health status of pre-treatment head and neck cancer patients [Article]. *BRITISH DENTAL JOURNAL*, *216*(1), Article E1. <https://doi.org/10.1038/sj.bdj.2013.1246>

da Silva, A. M., Falcão, M. M. L., Freitas, V. S., & Vieira, A. R. (2024). Genetic and environmental contributions for the relationship between tooth loss and oral potentially malignant disorders and oral squamous cell carcinoma [Article in Press]. *Head and Neck*. <https://doi.org/10.1002/hed.27648>

Daily, Z. A., Mohammed, N. B., Mohammed, S. M., & Hussein, H. M. (2025). Correlation Between Periodontal Disease and Oral, Oropharyngeal, and Parapharyngeal Cancers [Article]. *Clinical, Cosmetic and Investigational Dentistry*, *17*, 147-158. <https://doi.org/10.2147/CCIDE.S512557>

Dar, N. A., Islami, F., Bhat, G. A., Shah, I. A., Makhdoomi, M. A., Iqbal, B., Rafiq, R., Lone, M. M., Abnet, C. C., & Boffetta, P. (2013). Poor oral hygiene and risk of esophageal squamous cell carcinoma in Kashmir. *BRITISH JOURNAL OF CANCER*, *109*(5), 1367-1372. <https://doi.org/10.1038/bjc.2013.437>

Dhingra, K. (2022). Is periodontal disease a risk factor for oral cancer? [Review]. *Evidence-Based Dentistry*, *23*(1), 20-21. <https://doi.org/10.1038/s41432-022-0245-z>

Dholam, K. P., Sharma, M. R., Gurav, S. V., Singh, G. P., & Prabhash, K. (2021). Oral and dental health status in patients undergoing neoadjuvant chemotherapy for locally advanced head and neck cancer [Article]. *Oral Surgery, Oral Medicine, Oral Pathology and Oral Radiology*, *132*(5), 539-548. <https://doi.org/10.1016/j.oooo.2021.07.018>

Di Spirito, F., Amato, A., Romano, A., Dipalma, G., Xhajanka, E., Baroni, A., Serpico, R., Inchingolo, F., & Contaldo, M. (2022). Analysis of Risk Factors of Oral Cancer and Periodontitis from a Sex- and Gender-Related Perspective: Gender Dentistry. *APPLIED SCIENCES-BASEL*, *12*(18), Article 9135. <https://doi.org/10.3390/app12189135>

Dizdar, O., Hayran, M., Guven, D. C., Yılmaz, T. B., Taheri, S., Akman, A. C., Bilgin, E., Hüseyin, B., & Berker, E. (2017). Increased cancer risk in patients with periodontitis. *Curr Med Res Opin*, *33*(12), 2195-2200. <https://doi.org/10.1080/03007995.2017.1354829>

Epstein, J. B., Emerton, S., Lunn, R., Le, N., & Wong, F. L. W. (1999). Pretreatment assessment and dental management of patients with nasopharyngeal carcinoma [Article]. *ORAL ONCOLOGY*, *35*(1), 33-39. <https://doi.org/10.1016/S1368-8375(98)00072-4>

Epstein, J. B., Lunn, R., Le, N., & Stevenson-Moore, P. (1998). Periodontal attachment loss in patients after head and neck radiation therapy [Article]. *Oral Surgery, Oral Medicine, Oral Pathology, Oral Radiology, and Endodontics*, *86*(6), 673-677. <https://doi.org/10.1016/S1079-2104(98)90202-5>

Erira, A., Gamboa, F., Cid-Arregui, Á., Tobar-Tosse, F., & García, D. A. (2022). Transcriptomic characterization of bacteria related to eubiosis and dysbiosis in patients with and without oral squamous cell carcinoma [Article]. *Infectio*, *26*(4), 425-434. <https://doi.org/10.22354/24223794.1092>

Erira, A., García Robayo, D. A., Chalá, A. I., Moreno Torres, A., Muñoz Lopez, E. E., Cid Arregui, A., Tobar Tosse, F., & Gamboa Jaimes, F. O. (2021). Bacteriome Identified by Next-Generation Sequencing in Saliva, Dental Plaque, and Tumor Tissue of Patients with Oral Squamous Cell Carcinoma. *The Open Microbiology Journal*.

Escoda-Francolí, J., Rodríguez-Rodríguez, A., Pérez-García, S., Gargallo-Albiol, J., & Gay-Escoda, C. (2011). Dental implications in oral cancer patients [Article]. *Medicina Oral, Patologia Oral y Cirugia Bucal*, *16*(4), e508-e513. <https://doi.org/10.4317/medoral.16.e508>

Eun, Y. G., Lee, J. W., Kim, S. W., Hyun, D. W., Bae, J. W., & Lee, Y. C. (2021). Oral microbiome associated with lymph node metastasis in oral squamous cell carcinoma [Article]. *SCIENTIFIC REPORTS*, *11*(1), Article 23176. <https://doi.org/10.1038/s41598-021-02638-9>

Fitzsimonds, Z. R., Rodriguez-Hernandez, C. J., Bagaitkar, J., & Lamont, R. J. (2020). From Beyond the Pale to the Pale Riders: The Emerging Association of Bacteria with Oral Cancer. *JOURNAL OF DENTAL RESEARCH*, *99*(6), 604-612, Article 0022034520907341. <https://doi.org/10.1177/0022034520907341>

Fotedar, V., Ganju, S., Fotedar, S., Thakur, P., Sharma, A., & Bhardwaj, V. (2019). Oral microflora among oral cancer patients undergoing radiotherapy in regional cancer center, Indira Gandhi Medical College, Shimla [Article]. *Indian Journal of Medical and Paediatric Oncology*, *40*(5), S61-S64. <https://doi.org/10.4103/ijmpo.ijmpo_247_17>

Friemel, J., Foraita, R., Günther, K., Heibeck, M., Günther, F., Pflueger, M., Pohlabeln, H., Behrens, T., Bullerdiek, J., Nimzyk, R., & Ahrens, W. (2016). Pretreatment oral hygiene habits and survival of head and neck squamous cell carcinoma (HNSCC) patients. *BMC ORAL HEALTH*, *16*, Article 33. <https://doi.org/10.1186/s12903-016-0185-0>

Frydrych, A. M., & Slack-Smith, L. M. (2011). Dental attendance of oral and oropharyngeal cancer patients in a public hospital in Western Australia [Article]. *Australian Dental Journal*, *56*(3), 278-283. <https://doi.org/10.1111/j.1834-7819.2011.01343.x>

Fu, Y., & Zheng, Y. (2023). The identification of tumor antigens and immune subtypes based on the development of immunotherapies targeting head and neck squamous cell carcinomas resulting from periodontal disease [Article]. *FRONTIERS IN ONCOLOGY*, *13*, Article 1256105. <https://doi.org/10.3389/fonc.2023.1256105>

Gabusi, A., Gissi, D. B., Grillini, S., Stefanini, M., Tarsitano, A., Marchetti, C., Foschini, M. P., Montebugnoli, L., & Morandi, L. (2023). Shared epigenetic alterations between oral cancer and periodontitis: A preliminary study. *ORAL DISEASES*, *29*(5), 2052-2060. <https://doi.org/10.1111/odi.14251>

Gaetti-Jardim, E., Jardim, E. C. G., Schweitzer, C. M., da Silva, J. C. L., Oliveira, M. M., Masocatto, D. C., & dos Santos, C. M. (2018). Supragingival and subgingival microbiota from patients with poor oral hygiene submitted to radiotherapy for head and neck cancer treatment [Article]. *ARCHIVES OF ORAL BIOLOGY*, *90*, 45-52. <https://doi.org/10.1016/j.archoralbio.2018.01.003>

Ganly, I., Yang, L., Giese, R. A., Hao, Y., Nossa, C. W., Morris, L. G. T., Rosenthal, M., Migliacci, J., Kelly, D., Tseng, W., Hu, J., Li, H., Brown, S., & Pei, Z. (2019). Periodontal pathogens are a risk factor of oral cavity squamous cell carcinoma, independent of tobacco and alcohol and human papillomavirus. *Int J Cancer*, *145*(3), 775-784. <https://doi.org/10.1002/ijc.32152>

Gao, L., Hu, Y., Wang, Y., Jiang, W., He, Z., Zhu, C., Ma, R., & Huang, Z. (2015). Exploring the variation of oral microbiota in supragingival plaque during and after head-and-neck radiotherapy using pyrosequencing [Article]. *ARCHIVES OF ORAL BIOLOGY*, *60*(9), 1222-1230, Article 3390. <https://doi.org/10.1016/j.archoralbio.2015.05.006>

Garrote, L. F., Herrero, R., Reyes, R. M., Vaccarella, S., Anta, J. L., Ferbeye, L., Muñoz, N., & Franceschi, S. (2001). Risk factors for cancer of the oral cavity and oro-pharynx in Cuba. *Br J Cancer*, *85*(1), 46-54. <https://doi.org/10.1054/bjoc.2000.1825>

Ghanem, A. S., Sipos, K., Tóth, Á., & Nagy, A. C. (2025). Inflammatory Biomarkers and Oral Health Disorders as Predictors of Head and Neck Cancer: A Retrospective Longitudinal Study [Article]. *International Journal of Molecular Sciences*, *26*(5). <https://doi.org/10.3390/ijms26052279>

Gopinath, D., Kunnath Menon, R., Chun Wie, C., Banerjee, M., Panda, S., Mandal, D., Behera, P. K., Roychoudhury, S., Kheur, S., George Botelho, M., & Johnson, N. W. (2021). Salivary bacterial shifts in oral leukoplakia resemble the dysbiotic oral cancer bacteriome [Article]. *JOURNAL OF ORAL MICROBIOLOGY*, *13*(1), Article 1857998. <https://doi.org/10.1080/20002297.2020.1857998>

Gopinath, D., Menon, R. K., Wie, C. C., Banerjee, M., Panda, S., Mandal, D., Behera, P. K., Roychoudhury, S., Kheur, S., Botelho, M. G., & Johnson, N. W. (2021). Differences in the bacteriome of swab, saliva, and tissue biopsies in oral cancer [Article]. *SCIENTIFIC REPORTS*, *11*(1), Article 1181. <https://doi.org/10.1038/s41598-020-80859-0>

Guerrero-Preston, R., White, J. R., Godoy-Vitorino, F., Rodríguez-Hilario, A., Navarro, K., González, H., Michailidi, C., Jedlicka, A., Canapp, S., Bondy, J., Dziedzic, A., Lagos, B. M., Rivera-Alvarez, G., Ili-Gangas, C., Brebi-Mieville, P., Westra, W., Koch, W., Kang, H., Marchionni, L., . . . Sidransky, D. (2017). High-resolution microbiome profiling uncovers Fusobacterium nucleatum, Lactobacillus gasseri/johnsonii, and Lactobacillus vaginalis associated to oral and oropharyngeal cancer in saliva from HPV positive and HPV negative patients treated with surgery and chemo-radiation [Article]. *ONCOTARGET*, *8*(67), 110931-110948. <https://doi.org/10.18632/oncotarget.20677>

Guha, N., Boffetta, P., Wünsch Filho, V., Eluf Neto, J., Shangina, O., Zaridze, D., Curado, M. P., Koifman, S., Matos, E., Menezes, A., Szeszenia-Dabrowska, N., Fernandez, L., Mates, D., Daudt, A. W., Lissowska, J., Dikshit, R., & Brennan, P. (2007). Oral health and risk of squamous cell carcinoma of the head and neck and esophagus: results of two multicentric case-control studies. *Am J Epidemiol*, *166*(10), 1159-1173. <https://doi.org/10.1093/aje/kwm193>

Guo, Y. B., Liu, Y. W., Yang, H. J., Dai, N. T., Zhou, F. Y., Yang, H., Sun, W., Kong, J. Y., Yuan, X., & Gao, S. G. (2021). RETRACTED: Associations of <i>Porphyromonas gingivalis</i> Infection and Low Beclin1 Expression With Clinicopathological Parameters and Survival of Esophageal Squamous Cell Carcinoma Patients (Retracted Article). *PATHOLOGY & ONCOLOGY RESEARCH*, *27*, Article 1609976. <https://doi.org/10.3389/pore.2021.1609976>

Gupta, B., & Johnson, N. W. (2017). Oral cancer in Maharashtra, India: The influence of chewing tobacco, diet and oral hygiene [Conference Abstract]. *Head and Neck*, *39*, E221-E222. <https://www.embase.com/search/results?subaction=viewrecord&id=L616608007&from=export>

Güven, D. C., Dizdar, Ö., Akman, A. C., Berker, E., Yekedüz, E., Ceylan, F., Başpınar, B., Akbıyık, İ., Aktaş, B. Y., Yüce, D., Erman, M., & Hayran, M. (2019). Evaluation of cancer risk in patients with periodontal diseases. *Turk J Med Sci*, *49*(3), 826-831. <https://doi.org/10.3906/sag-1812-8>

Han, Y. W., Houcken, W., Loos, B. G., Schenkein, H. A., & Tezal, M. (2014). Periodontal disease, atherosclerosis, adverse pregnancy outcomes, and head-and-neck cancer [Review]. *Advances in Dental Research*, *26*(1), 47-55. <https://doi.org/10.1177/0022034514528334>

Hashim, D., Sartori, S., Brennan, P., Curado, M. P., Wünsch, V., Divaris, K., Olshan, A. F., Zevallos, J. P., Winn, D. M., Franceschi, S., Castellsagué, X., Lissowska, J., Rudnai, P., Matsuo, K., Morgenstern, H., Chen, C., Vaughan, T. L., Hofmann, J. N., D'Souza, G., . . . Boffetta, P. (2016). The role of oral hygiene in head and neck cancer: results from International Head and Neck Cancer Epidemiology (INHANCE) consortium. *ANNALS OF ONCOLOGY*, *27*(8), 1619-1625. <https://doi.org/10.1093/annonc/mdw224>

Hashimoto, K., Shimizu, D., Hirabayashi, S., Ueda, S., Miyabe, S., Oh-iwa, I., Nagao, T., Shimozato, K., & Nomoto, S. (2019). Changes in oral microbial profiles associated with oral squamous cell carcinoma vs leukoplakia [Article]. *Journal of Investigative and Clinical Dentistry*, *10*(4), Article e12445. <https://doi.org/10.1111/jicd.12445>

Hashimoto, K., Shimizu, D., Ueda, S., Miyabe, S., Oh-Iwa, I., Nagao, T., Shimozato, K., & Nomoto, S. (2022). Feasibility of oral microbiome profiles associated with oral squamous cell carcinoma [Article]. *JOURNAL OF ORAL MICROBIOLOGY*, *14*(1), Article 2105574. <https://doi.org/10.1080/20002297.2022.2105574>

Hasnat, S., Metsaeniitty, M., Nurmi, K., Eklund, K. K., & Salem, A. (2025). Intracellular bacterial LPS drives pyroptosis and promotes aggressive phenotype in oral squamous cell carcinoma. *Medical Oncology*, *42*(6), Article 205. <https://doi.org/10.1007/s12032-025-02766-6>

Hayes, R. B., Ahn, J., Fan, X., Peters, B. A., Ma, Y., Yang, L., Agalliu, I., Burk, R. D., Ganly, I., Purdue, M. P., Freedman, N. D., Gapstur, S. M., & Pei, Z. (2018). Association of Oral Microbiome With Risk for Incident Head and Neck Squamous Cell Cancer. *JAMA Oncol*, *4*(3), 358-365. <https://doi.org/10.1001/jamaoncol.2017.4777>

Heikkilä, P., But, A., Sorsa, T., & Haukka, J. (2018). Periodontitis and cancer mortality: Register-based cohort study of 68,273 adults in 10-year follow-up [Article]. *INTERNATIONAL JOURNAL OF CANCER*, *142*(11), 2244-2253. <https://doi.org/10.1002/ijc.31254>

Heng, W., Wang, W., Dai, T., Jiang, P., Lu, Y., Li, R., Zhang, M., Xie, R., Zhou, Y., Zhao, M., Duan, N., Ye, Z., Yan, F., & Wang, X. (2022). Oral Bacteriome and Mycobiome across Stages of Oral Carcinogenesis [Article]. *Microbiology Spectrum*, *10*(6). <https://doi.org/10.1128/spectrum.02737-22>

Heredia, G. L. G., Nuñez, M. O., Rivas, I. C., Guerrero, J. J. M., & Herrera, D. G. B. (2017). Oral manifestations due to radiotherapy in geriatric patients with head and neck cancer [Article]. *Revista Cubana de Estomatologia*, *54*(4). <https://www.scopus.com/inward/record.uri?eid=2-s2.0-85041722403&partnerID=40&md5=e21ad7e6f791ed2b1e0cfc13135b168b>

Herreros-Pomares, A., Hervás, D., Bagan-Debon, L., Jantus-Lewintre, E., Gimeno-Cardona, C., & Bagan, J. (2023). On the Oral Microbiome of Oral Potentially Malignant and Malignant Disorders: Dysbiosis, Loss of Diversity, and Pathogens Enrichment. *INTERNATIONAL JOURNAL OF MOLECULAR SCIENCES*, *24*(4), Article 3466. <https://doi.org/10.3390/ijms24043466>

Hosainzadegan, H., Parvan, R., & Hosainzadegan, M. (2022). A retrospective study comparing oral health in cancer patients and healthy people [Article]. *European Journal of Translational Myology*, *32*(4), Article 10672. <https://doi.org/10.4081/ejtm.2022.10672>

Hsiao, J. R., Chang, C. C., Lee, W. T., Huang, C. C., Ou, C. Y., Tsai, S. T., Chen, K. C., Huang, J. S., Wong, T. Y., Lai, Y. H., Wu, Y. H., Hsueh, W. T., Wu, S. Y., Yen, C. J., Chang, J. Y., Lin, C. L., Weng, Y. L., Yang, H. C., Chen, Y. S., & Chang, J. S. (2018). The interplay between oral microbiome, lifestyle factors and genetic polymorphisms in the risk of oral squamous cell carcinoma. *CARCINOGENESIS*, *39*(6), 778-787. <https://doi.org/10.1093/carcin/bgy053>

Iturbide, A., Dhaliwal, V., Noll, J., Hodges, J., Von Bültzingslöwen, I., & Brennan, M. (2017). Pre-radiation dental treatment in the head and neck cancer patient [Conference Abstract]. *SUPPORTIVE CARE IN CANCER*, *25*(2), S152-S153. <https://doi.org/10.1007/s00520-017-3704-x>

Jácome-Santos, H., da Silva E Silva, N., Resende, R. G., Costa Pinheiro, H. H., Almeida Machado, L. F., de Souza Silva, G., de Oliveira Costa, F., Brasil-Costa, I., Amoras-Alves, A. C. B., Mesquita, R. A., & de Melo Alves-Junior, S. (2022). Simultaneous occurrence of Epstein-Barr virus (EBV) in periodontal pockets and in oral squamous cell carcinoma: a cross-sectional study [Article]. *CLINICAL ORAL INVESTIGATIONS*, *26*(3), 2807-2815. <https://doi.org/10.1007/s00784-021-04258-z>

Jebril, A., Jarad, F., Butterworth, C. J., & Bebb, K. (2024). Assessment of oral disease burden among head and neck cancer patients in the Merseyside region [Article]. *British Dental Journal*. <https://doi.org/10.1038/s41415-024-8060-x>

John, A. A., Kumar, C. N., Ranganath, V., Rao, S. M., Patil, A. S., & Jumani, P. N. (2019). Relationship between the nutritional status and antimicrobial protein levels with the periodontal condition in untreated head and neck cancer patients. *JOURNAL OF FAMILY MEDICINE AND PRIMARY CARE*, *8*(10), 3325-3333. <https://doi.org/10.4103/jfmpc.jfmpc_658_19>

Jouhi, L., Sikiö, J., Suomalainen, A., Mroueh, R., Mäkitie, A., & Meurman, J. H. (2022). Dental health in patients with and without HPV-positive oropharyngeal and tongue cancer [Article]. *PLOS ONE*, *17*(9 September), Article e0274813. <https://doi.org/10.1371/journal.pone.0274813>

Kaliamoorthy, S., Saranyan, R., Govindasamy, A., & Balakrishnan, J. (2021). Exploring the presence of Treponema denticola in oral squamous cell carcinoma [Article]. *Journal of Medical Pharmaceutical and Allied Sciences*, *10*(5), 3686-3688. <https://doi.org/10.22270/jmpas.V10I5.1679>

Kamarajan, P., Ateia, I., Shin, J. M., Fenno, J. C., & Kapila, Y. L. (2016). Treponema denticola, a periodontal pathogen, promotes stemness and migration in oral squamous cell carcinoma [Conference Abstract]. *Cancer Research*, *76*(14). <https://doi.org/10.1158/1538-7445.AM2016-3294>

Kang, M.-S., Oh, J.-S., Kim, H.-J., Kim, H.-N., Lee, I.-K., Choi, H.-R., Kim, O.-J., Ko, Y.-J., Lim, W.-B., Park, H.-J., Yu, M.-G., Chung, K.-Y., Kim, S.-M., & Lim, H.-S. (2009). Prevalence of Oral Microbes in the Saliva of Oncological Patients. *jbv*, *39*(4), 277-285. <https://doi.org/10.4167/jbv.2009.39.4.277>

Karri, R. L., Amrutha, R., Shrinivas, Bojji, M., Kumar, K. M., & Benarji, K. A. (2024). Analyzing Pooled Microarray Gene Expression Data to Uncover Common Pathways in Periodontitis and Oral Squamous Cell Carcinoma from the Gene Expression Omnibus. *J Pharm Bioallied Sci*, *16*(Suppl 2), S1515-s1521. <https://doi.org/10.4103/jpbs.jpbs_1180_23>

Kasimov, A. E., Grigorievskaya, Z. V., Kropotov, M. A., Bagirova, N. S., Petukhova, I. N., Tereshchenko, I. V., & Pak, M. B. (2021). Periodontal pathogens as a risk factor for oral squamous cell carcinoma [Article]. *Opuholi Golovy i Sei*, *11*(3), 83-93. <https://doi.org/10.17650/2222-1468-2021-11-3-83-93>

Katz, J., Onate, M. D., Pauley, K. M., Bhattacharyya, I., & Cha, S. (2011). Presence of Porphyromonas gingivalis in gingival squamous cell carcinoma. *Int J Oral Sci*, *3*(4), 209-215. <https://doi.org/10.4248/ijos11075>

Keskin, M., Lähteenmäki, H., Rathnayake, N., Räisänen, I. T., Tervahartiala, T., Pärnänen, P., Şenışık, A. M., Karaçetin, D., Yentek Balkanay, A., Heikkilä, P., Hagström, J., Rautava, J., Haglund, C., Gursoy, U. K., Silbereisen, A., Bostanci, N., & Sorsa, T. (2020). Active matrix metalloproteinase-8 and interleukin-6 detect periodontal degeneration caused by radiotherapy of head and neck cancer: a pilot study [Article]. *Expert Review of Proteomics*, *17*(10), 777-784. <https://doi.org/10.1080/14789450.2020.1858056>

Khan, M., Khan, S., Mandal, R. K., Mahto, H. S., Lohani, M., Ahmad, S., Sherwani, S., Jandrajupalli, S. B., & Haque, S. (2021). Cell Cycle Regulatory CCND1 G870A Gene Polymorphism and Periodontitis-Induced Oral Cancer: A Risk Analysis. *REVISTA ROMANA DE MEDICINA DE LABORATOR*, *29*(4), 349-363. <https://doi.org/10.2478/rrlm-2021-0028>

Kim, Y. K., Kwon, E. J., Yu, Y., Kim, J., Woo, S. Y., Choi, H. S., Kwon, M., Jung, K., Kim, H. S., Park, H. R., Lee, D., & Kim, Y. H. (2022). Microbial and molecular differences according to the location of head and neck cancers. *CANCER CELL INTERNATIONAL*, *22*(1), Article 135. <https://doi.org/10.1186/s12935-022-02554-6>

Kim, Y. T., Kang, M. J., Lee, B. A., Kang, S. H., & Kim, R. H. (2025). Risk factors and incidence of oral tumors: Findings from a longitudinal population-based study [Article]. *Oral Diseases*, *31*(3), 846-856. <https://doi.org/10.1111/odi.15125>

Kindler, S., Samietz, S., Dickel, S., Mksoud, M., Kocher, T., Lucas, C., Seebauer, C., Doberschütz, P., Holtfreter, B., Völzke, H., Metelmann, H. R., & Ittermann, T. (2021). Prevalence and risk factors of potentially malignant disorders of the mucosa in the general population: Mucosa lesions a general health problem? [Article]. *Annals of Anatomy*, *237*, Article 151724. <https://doi.org/10.1016/j.aanat.2021.151724>

Kioi, M., Isono, H., & Nakajima, S. (2023). Specific oral microbiome is closely associated with oral potentially malignant disorders and oral squamous cell carcinoma [Conference Abstract]. *Cancer Research*, *83*(7). <https://doi.org/10.1158/1538-7445.AM2023-4238>

Ko, H. H., Wu, F. Y., Kao, H. F., Lin, W., Chen, Y. S., & Cheng, S. J. (2025). Exploring gender-specific prognostic factors and survival outcomes in oral squamous cell carcinoma: Insights from a Taiwanese cohort [Article]. *Journal of Dental Sciences*. <https://doi.org/10.1016/j.jds.2025.04.026>

Kozłowski, Z., Konopka, T., Karolewska, E., Kaczmarek, U., & Wnukiewicz, J. (2009). Evaluation of nitric oxide levels in the saliva of patients with oral squamous carcinoma and chronic periodontitis [Article]. *Dental and Medical Problems*, *46*(1), 55-62. <https://www.scopus.com/inward/record.uri?eid=2-s2.0-81455161822&partnerID=40&md5=7a40d229cd4a88c317992db956b95666>

Krüger, M., Hansen, T., Kasaj, A., & Moergel, M. (2013). The Correlation between Chronic Periodontitis and Oral Cancer. *Case Rep Dent*, *2013*, 262410. <https://doi.org/10.1155/2013/262410>

Kylmä, A. K., Jouhi, L., Listyarifah, D., Mohamed, H., Mäkitie, A., Remes, S. M., Haglund, C., Atula, T., Nieminen, M. T., Sorsa, T., & Hagström, J. (2018). Treponema denticola chymotrypsin-like protease as associated with HPV-negative oropharyngeal squamous cell carcinoma [Article]. *BRITISH JOURNAL OF CANCER*, *119*(1), 89-95. <https://doi.org/10.1038/s41416-018-0143-5>

Kylmae, A. K., Sorsa, T., Jouhi, L., Mustonen, H. K., Mohamed, H., Randen-brady, R., Maekitie, A., Atula, T., Hagstrom, J., & Haglund, C. (2022). Prognostic Role of Porphyromonas gingivalis Gingipain Rgp and Matrix Metalloproteinase 9 in Oropharyngeal Squamous Cell Carcinoma. *ANTICANCER RESEARCH*, *42*(11), 5415-5430. <https://doi.org/10.21873/anticanres.16046>

Laheij, A. M., de Soet, J. J., Veerman, E. C., Bolscher, J. G., & van Loveren, C. (2013). The influence of oral bacteria on epithelial cell migration in vitro. *Mediators Inflamm*, *2013*, 154532. <https://doi.org/10.1155/2013/154532>

Laliberte, C., Ng, N., Eymael, D., Higgins, K., Ali, A., Kiss, A., Bradley, G., & Magalhaes, M. A. O. (2021). Characterization of Oral Squamous Cell Carcinoma Associated Inflammation: A Pilot Study. *FRONTIERS IN ORAL HEALTH*, *2*, Article 740469. <https://doi.org/10.3389/froh.2021.740469>

Lalla, R. V., Long-Simpson, L., Hodges, J. S., Treister, N., Sollecito, T., Schmidt, B., Patton, L. L., & Brennan, M. T. (2017). Clinical registry of dental outcomes in head and neck cancer patients (OraRad): Rationale, methods, and recruitment considerations [Article]. *BMC ORAL HEALTH*, *17*(1), Article 59. <https://doi.org/10.1186/s12903-017-0344-y>

Lenz, B., Crameri, F. M., Eichler, D. A., Schläppi, B., Wiltshire, H. R., Wood, J., & Seymour, R. A. (2005). Modulation of oral squamous cell carcinoma incidence in rats via diet and a novel calcium channel antagonist. *TOXICOLOGIC PATHOLOGY*, *33*(3), 356-364. <https://doi.org/10.1080/01926230590930119>

Li, S., Liu, X., Zhou, Y., Acharya, A., Savkovic, V., Xu, C., Wu, N., Deng, Y., Hu, X., Li, H., Haak, R., Schmidt, J., Shang, W., Pan, H., Shang, R., Yu, Y., Ziebolz, D., & Schmalz, G. (2018). Shared genetic and epigenetic mechanisms between chronic periodontitis and oral squamous cell carcinoma [Article]. *ORAL ONCOLOGY*, *86*, 216-224. <https://doi.org/10.1016/j.oraloncology.2018.09.029>

Li, Y., Tan, X., Zhao, X., Xu, Z., Dai, W., Duan, W., Huang, S., Zhang, E., Liu, J., Zhang, S., Yin, R., Shi, X., Lu, Z., & Pan, Y. (2020). Composition and function of oral microbiota between gingival squamous cell carcinoma and periodontitis. *Oral Oncol*, *107*, 104710. <https://doi.org/10.1016/j.oraloncology.2020.104710>

Li, Z., Fu, R., Wen, X., & Zhang, L. (2023). Network analysis reveals miRNA crosstalk between periodontitis and oral squamous cell carcinoma. *BMC ORAL HEALTH*, *23*(1), 19. <https://doi.org/10.1186/s12903-022-02704-2>

Li, Z. X., Chen, G., Wang, P. P., Sun, M. L., Zhao, J. F., Li, A., & Sun, Q. (2021). Alterations of the Oral Microbiota Profiles in Chinese Patient With Oral Cancer. *FRONTIERS IN CELLULAR AND INFECTION MICROBIOLOGY*, *11*, Article 780067. <https://doi.org/10.3389/fcimb.2021.780067>

Lim, Y., Fukuma, N., Totsika, M., Kenny, L., Morrison, M., & Punyadeera, C. (2018). The Performance of an Oral Microbiome Biomarker Panel in Predicting Oral Cavity and Oropharyngeal Cancers. *FRONTIERS IN CELLULAR AND INFECTION MICROBIOLOGY*, *8*, Article 267. <https://doi.org/10.3389/fcimb.2018.00267>

Lin, F. Y., Huang, C. Y., Lu, H. Y., Shih, C. M., Tsao, N. W., Shyue, S. K., Lin, C. Y., Chang, Y. J., Tsai, C. S., Lin, Y. W., & Lin, S. J. (2015). The GroEL protein of Porphyromonas gingivalis accelerates tumor growth by enhancing endothelial progenitor cell function and neovascularization [Article]. *MOLECULAR ORAL MICROBIOLOGY*, *30*(3), 198-216. <https://doi.org/10.1111/omi.12083>

Listyarifah, D., Nieminen, M. T., Mäkinen, L. K., Haglund, C., Grenier, D., Häyry, V., Nordström, D., Hernandez, M., Yucel-Lindberg, T., Tervahartiala, T., Ainola, M., Sorsa, T., & Hagström, J. (2018). Treponema denticola chymotrypsin-like proteinase is present in early-stage mobile tongue squamous cell carcinoma and related to the clinicopathological features. *J Oral Pathol Med*, *47*(8), 764-772. <https://doi.org/10.1111/jop.12729>

Liu, T., Li, G., Huang, Z., Bu, X., Ma, J., Duan, N., Wang, W., & Wang, X. (2025). Study on the invasion of Prevotella intermedia in tissues during carcinogenesis of oral mucosa [Article]. *Chinese Journal of Stomatology*, *60*(3), 211-222. <https://doi.org/10.3760/cma.j.cn112144-20241212-00476>

Lockhart, P. B., & Clark, J. (1994). Pretherapy dental status of patients with malignant conditions of the head and neck [Article]. *Oral Surgery, Oral Medicine, Oral Pathology*, *77*(3), 236-241. <https://doi.org/10.1016/0030-4220(94)90291-7>

Longo, B. C., Rohling, I. B., Silva, P. L. M. O. E., de Morais, M. E. F., Paz, H. E. S., Casarin, R. C. V., Nishiyama, S. A. B., de Souza, M. D. B., & Silva, C. O. (2023). Antineoplastic therapy in childhood cancer patients presents a negative impact in the periodontal tissues: a cohort study [Article]. *CLINICAL ORAL INVESTIGATIONS*, *27*(11), 6637-6644. <https://doi.org/10.1007/s00784-023-05270-1>

Lyu, W. N., Lin, M. C., Lou, P. J., Lai, L. C., & Tsai, M. H. (2023). Identification of microbial biomarkers to predict recurrence of oral squamous cell carcinoma [Conference Abstract]. *Cancer Research*, *83*(7). <https://doi.org/10.1158/1538-7445.AM2023-5896>

Mager, D. L., Haffajee, A. D., Devlin, P. M., Norris, C. M., Posner, M. R., & Goodson, J. M. (2005). The salivary microbiota as a diagnostic indicator of oral cancer: A descriptive, non-randomized study of cancer-free and oral squamous cell carcinoma subjects. *JOURNAL OF TRANSLATIONAL MEDICINE*, *3*. <https://doi.org/10.1186/1479-5876-3-27>

Magrin, G. L., Di Summa, F., Strauss, F. J., Panahipour, L., Mildner, M., Benfatti, C. A. M., & Gruber, R. (2020). Butyrate Decreases ICAM-1 Expression in Human Oral Squamous Cell Carcinoma Cells. *INTERNATIONAL JOURNAL OF MOLECULAR SCIENCES*, *21*(5), Article 1679. <https://doi.org/10.3390/ijms21051679>

Mäkinen, A. I., Pappalardo, V. Y., Buijs, M. J., Brandt, B. W., Mäkitie, A. A., Meurman, J. H., & Zaura, E. (2023). Salivary microbiome profiles of oral cancer patients analyzed before and after treatment [Article]. *Microbiome*, *11*(1), Article 171. <https://doi.org/10.1186/s40168-023-01613-y>

Marinescu-Gava, M., Keinänen, A., Uittamo, J., & Snäll, J. (2019). Dental findings in tonsil carcinoma patients [Conference Abstract]. *International Journal of Oral and Maxillofacial Surgery*, *48*, 235-236. <https://doi.org/10.1016/j.ijom.2019.03.721>

Mauceri, R., Coppini, M., Vacca, D., Bertolazzi, G., Cancila, V., Tripodo, C., & Campisi, G. (2023). No Clear Clustering Dysbiosis from Salivary Microbiota Analysis by Long Sequencing Reads in Patients Affected by Oral Squamous Cell Carcinoma: A Single Center Study [Article]. *CANCERS*, *15*(17), Article 4211. <https://doi.org/10.3390/cancers15174211>

Medeiros, M. C., The, S., Bellile, E., Russo, N., Schmitd, L., Danella, E., Singh, P., Banerjee, R., Bassis, C., Murphy, G. R., Sartor, M. A., Lombaert, I., Schmidt, T. M., Eisbruch, A., Murdoch-Kinch, C. A., Rozek, L., Wolf, G. T., Li, G., Chen, G. Y., & D’Silva, N. J. (2023). Salivary microbiome changes distinguish response to chemoradiotherapy in patients with oral cancer [Article]. *Microbiome*, *11*(1), Article 268. <https://doi.org/10.1186/s40168-023-01677-w>

Meng, L., Zhao, Y., Bu, W., Li, X., Liu, X., Zhou, D., Chen, Y., Zheng, S., Lin, Q., Liu, Q., & Sun, H. (2020). Bone mesenchymal stem cells are recruited via CXCL8-CXCR2 and promote EMT through TGF-β signal pathways in oral squamous carcinoma. *Cell Prolif*, *53*(8), e12859. <https://doi.org/10.1111/cpr.12859>

Meyle, J., Domann, E., Charkraborty, T., Ruf, S., & Groeger, S. (2022). Immune response and metabolic gene expression induced by P. gingivalis FimA [Conference Abstract]. *JOURNAL OF CLINICAL PERIODONTOLOGY*, *49*, 126. <https://doi.org/10.1111/jcpe.13635>

Michaud, D. S., Fu, Z., Shi, J., & Chung, M. (2017). Periodontal Disease, Tooth Loss, and Cancer Risk. *Epidemiol Rev*, *39*(1), 49-58. <https://doi.org/10.1093/epirev/mxx006>

Michaud, D. S., Liu, Y., Meyer, M., Giovannucci, E., & Joshipura, K. (2008). Periodontal disease, tooth loss, and cancer risk in male health professionals: a prospective cohort study. *Lancet Oncol*, *9*(6), 550-558. <https://doi.org/10.1016/s1470-2045(08)70106-2>

Mok, S. F., Karuthan, C., Cheah, Y. K., Ngeow, W. C., Rosnah, B. Z., Yap, S. F., & Ong, H. K. A. (2017). The oral microbiome community variations associated with normal, potentially malignant disorders and malignant lesions of the oral cavity [Article]. *Malaysian Journal of Pathology*, *39*(1), 1-15. <https://www.scopus.com/inward/record.uri?eid=2-s2.0-85037035714&partnerID=40&md5=e5a85691892ee13a6be7e7e9dfe61a69>

Monier, N. M., Atteya, I. M., Askar, H., & Helmy, S. (2020). Detection of the periodontal pathogen Porphyromonas Gingivalis in Oral Squamous Cell Carcinoma. *Mansoura Journal of Dentistry*, *7*(3), 24-28. <https://doi.org/10.21608/mjd.2020.198725>

Moreno-López, L. A., Esparza-Gómez, G. C., González-Navarro, A., Cerero-Lapiedra, R., González-Hernández, M. J., & Domínguez-Rojas, V. (2000). Risk of oral cancer associated with tobacco smoking, alcohol consumption and oral hygiene: a case-control study in Madrid, Spain. *Oral Oncol*, *36*(2), 170-174. <https://doi.org/10.1016/s1368-8375(99)00084-6>

Na, H. S., Kim, S., Choi, Y. H., Lee, J.-Y., & Chung, J. (2013). Oral Microbiota Comparison between Healthy volunteers, Periodontitis patients and Oral cancer patients. *International Journal of Oral Biology*, *38*(4), 181-188.

Naavaal, S., Garcia, D. T., Deng, X., & Bandyopadhyay, D. (2022). Association between periodontal disease and oral cancer screening among US adults: NHANES 2011-2014 [Article]. *COMMUNITY DENTISTRY AND ORAL EPIDEMIOLOGY*, *50*(3), 216-224. <https://doi.org/10.1111/cdoe.12655>

Nagy, K. N., Sonkodi, I., Szöke, I., Nagy, E., & Newman, H. N. (1998). The microflora associated with human oral carcinomas [Article]. *ORAL ONCOLOGY*, *34*(4), 304-308. <https://doi.org/10.1016/S1368-8375(98)00008-6>

Nie, F., Wang, L., Huang, Y., Yang, P., Gong, P., Feng, Q., & Yang, C. (2022). Characteristics of Microbial Distribution in Different Oral Niches of Oral Squamous Cell Carcinoma [Article]. *FRONTIERS IN CELLULAR AND INFECTION MICROBIOLOGY*, *12*, Article 905653. <https://doi.org/10.3389/fcimb.2022.905653>

Nieminen, M. T., Listyarifah, D., Hagström, J., Haglund, C., Grenier, D., Nordström, D., Uitto, V. J., Hernandez, M., Yucel-Lindberg, T., Tervahartiala, T., Ainola, M., & Sorsa, T. (2018). Treponema denticola chymotrypsin-like proteinase may contribute to orodigestive carcinogenesis through immunomodulation. *Br J Cancer*, *118*(3), 428-434. <https://doi.org/10.1038/bjc.2017.409>

Nierengarten, M. B. (2024). Oral health linked to survival in head and neck cancer [Note]. *CANCER*, *130*(2), 171. <https://doi.org/10.1002/cncr.35161>

Nikakhlagh, S., Saki, N., shoar, M. H., Sartipipor, A., & Saki, S. (2012). Incidence of etiologic factors in squamous cell carcinoma of head and neck in Ahvaz [Article]. *Iranian Journal of Otorhinolaryngology*, *24*(67), 85-90. <https://www.scopus.com/inward/record.uri?eid=2-s2.0-84860486041&partnerID=40&md5=14546e80a3b9dc63998bcebee2533395>

Nitescu, D. C. K., Constantin, M., Oanta, C., Martu, I., Volovat, S. R., & Martu, S. (2017). Evaluation of Cumulative Effects of Chemotherapy and Bevacizumab (Avastin) in Oncological Patients with Periodontal Disease. *REVISTA DE CHIMIE*, *68*(3), 549-552.

Nuñez-Aguilar, J., Fernández-Olavarría, A., Oliveros-López, L. G., Torres-Lagares, D., Serrera-Figallo, M. A., Gutiérrez-Corrales, A., & Gutiérrez-Pérez, J. L. (2018). Evolution of oral health in oral cancer patients with and without dental treatment in place: Before, during and after cancer treatment [Article]. *Journal of Clinical and Experimental Dentistry*, *10*(2), e158-e165. <https://doi.org/10.4317/jced.54608>

Oballe, H. J. R., Muniz, F., Bueno, C. C., Klein, I. P., Carrard, V. C., Rösing, C. K., & Gaio, E. J. (2018). Spontaneous alveolar bone loss after 4NQO exposure in Wistar rats. *ARCHIVES OF ORAL BIOLOGY*, *89*, 44-48. <https://doi.org/10.1016/j.archoralbio.2018.02.001>

Ohshima, J., Wang, Q., Fitzsimonds, Z. R., Miller, D. P., Sztukowska, M. N., Jung, Y. J., Hayashi, M., Whiteley, M., & Lamont, R. J. (2019). Streptococcus gordonii programs epithelial cells to resist ZEB2 induction by Porphyromonas gingivalis. *Proc Natl Acad Sci U S A*, *116*(17), 8544-8553. <https://doi.org/10.1073/pnas.1900101116>

Panda, M., Rai, A. K., Rahman, T., Das, A., Das, R., Sarma, A., Kataki, A. C., & Chattopadhyay, I. (2020). Alterations of salivary microbial community associated with oropharyngeal and hypopharyngeal squamous cell carcinoma patients. *ARCHIVES OF MICROBIOLOGY*, *202*(4), 785-805. <https://doi.org/10.1007/s00203-019-01790-1>

Park, D. G., Woo, B. H., Lee, B. J., Yoon, S., Cho, Y., Kim, Y. D., Park, H. R., & Song, J. M. (2019). Serum Levels of Interleukin-6 and Titers of Antibodies against Porphyromonas gingivalis Could Be Potential Biomarkers for the Diagnosis of Oral Squamous Cell Carcinoma. *INTERNATIONAL JOURNAL OF MOLECULAR SCIENCES*, *20*(11), Article 2749. <https://doi.org/10.3390/ijms20112749>

Perera, I. R., Attygalla, M., Jayasuriya, N., Dias, D. K., & Perera, M. L. (2018). Oral hygiene and periodontal disease in male patients with oral cancer. *Br J Oral Maxillofac Surg*, *56*(9), 901-903. <https://doi.org/10.1016/j.bjoms.2018.09.015>

Pranata, N., Maskoen, A. M., Sahiratmadja, E., & Widyaputra, S. (2020). Dental Calculus as a Potential Biosource for Human Papillomavirus Detection in Oral Squamous Cell Carcinoma [Article]. *ASIAN PACIFIC JOURNAL OF CANCER PREVENTION*, *21*(10), 3093-3097. <https://doi.org/10.31557/APJCP>. 2020.21.10.3093

Pushalkar, S., Ji, X. J., Li, Y. H., Estilo, C., Yegnanarayana, R., Singh, B., Li, X., & Saxena, D. (2012). Comparison of oral microbiota in tumor and non-tumor tissues of patients with oral squamous cell carcinoma. *BMC MICROBIOLOGY*, *12*, Article 144. <https://doi.org/10.1186/1471-2180-12-144>

Rai, A. K., Panda, M., Das, A. K., Rahman, T., Das, R., Das, K., Sarma, A., Kataki, A. C., & Chattopadhyay, I. (2021). Dysbiosis of salivary microbiome and cytokines influence oral squamous cell carcinoma through inflammation [Article]. *ARCHIVES OF MICROBIOLOGY*, *203*(1), 137-152. <https://doi.org/10.1007/s00203-020-02011-w>

Rani, N. A. J., Vardhan, B. G. H., Srinivasan, S., & Gopal, S. K. (2023). Evaluation of Salivary Interleukin-6 in Patients with Oral Squamous Cell Carcinoma, Oral Potentially Malignant Disorders, Chronic Periodontitis and in Healthy Controls - A Cross-Sectional Comparative Study [Article]. *Annals of Maxillofacial Surgery*, *13*(1), 70-75. <https://doi.org/10.4103/ams.ams_240_22>

Rodrigues, I., Botelho, J., Machado, V., Proença, L., Mendes, J. J., & Zagalo, C. (2023). Profiling oral health status, values, and related quality of life in patients with oral cancer: a pilot study. *FRONTIERS IN ORAL HEALTH*, *4*, Article 1268657. <https://doi.org/10.3389/froh.2023.1268657>

Rouers, M., Dubourg, S., Bornert, F., Truntzer, P., Antoni, D., Couchot, J., Ganansia, V., Bourrier, C., Guihard, S., & Noël, G. (2016). Orodental status before radiation therapy of the head and neck area: A prospective analysis on 48 patients [Article]. *Cancer/Radiotherapie*, *20*(3), 199-204. <https://doi.org/10.1016/j.canrad.2015.12.008>

Rupe, C., Basco, A., Schiavelli, A., Cassano, A., Micciche, F., Galli, J., Cordaro, M., & Lajolo, C. (2022). Oral Health Status in Patients with Head and Neck Cancer before Radiotherapy: Baseline Description of an Observational Prospective Study. *Cancers (Basel)*, *14*(6). <https://doi.org/10.3390/cancers14061411>

Sahni, V. (2023). Is there an association between oral hygiene and head and neck cancer? *Evid Based Dent*, *24*(2), 57-58. <https://doi.org/10.1038/s41432-023-00879-3>

Sarkar, P., Malik, S., Laha, S., Das, S., Bunk, S., Ray, J. G., Chatterjee, R., & Saha, A. (2021). Dysbiosis of Oral Microbiota During Oral Squamous Cell Carcinoma Development [Article]. *FRONTIERS IN ONCOLOGY*, *11*, Article 614448. <https://doi.org/10.3389/fonc.2021.614448>

Sawant, S., Dugad, J., Parikh, D., Srinivasan, S., & Singh, H. (2021). Identification & correlation of bacterial diversity in oral cancer and long-term tobacco chewers- A case-control pilot study [Article]. *JOURNAL OF MEDICAL MICROBIOLOGY*, *70*(9), Article 001417. <https://doi.org/10.1099/jmm.0.001417>

Saxena, R., Vishnu Prasoodanan, P. K., Gupta, S. V., Gupta, S., Waiker, P., Samaiya, A., Sharma, A. K., & Sharma, V. K. (2022). Assessing the Effect of Smokeless Tobacco Consumption on Oral Microbiome in Healthy and Oral Cancer Patients [Article]. *FRONTIERS IN CELLULAR AND INFECTION MICROBIOLOGY*, *12*, Article 841465. <https://doi.org/10.3389/fcimb.2022.841465>

Schmidt, B. L., Kuczynski, J., Bhattacharya, A., Huey, B., Corby, P. M., Queiroz, E. L. S., Nightingale, K., Kerr, A. R., DeLacure, M. D., Veeramachaneni, R., Olshen, A. B., & Albertson, D. G. (2014). Changes in abundance of oral microbiota associated with oral cancer [Article]. *PLOS ONE*, *9*(6), Article e98741. <https://doi.org/10.1371/journal.pone.0098741>

Shankarram, V., Narayanan, L., Selvan, T., Sudhakar, U., Moses, J., & Parthiban, S. (2015). Detection of oxidative stress in periodontal disease and oral cancer [Article]. *Biomedical and Pharmacology Journal*, *8*(2), 725-729. <https://doi.org/10.13005/bpj/819>

Shay, E., Sangwan, N., Padmanabhan, R., Lundy, S., Burkey, B., & Eng, C. (2020). Bacteriome and mycobiome and bacteriome-mycobiome interactions in head and neck squamous cell carcinoma [Article]. *ONCOTARGET*, *11*(25), 2375-2386. <https://doi.org/10.18632/oncotarget.27629>

Shin, J. M., Luo, T., Kamarajan, P., Fenno, J. C., Rickard, A. H., & Kapila, Y. L. (2017). Microbial Communities Associated with Primary and Metastatic Head and Neck Squamous Cell Carcinoma - A High Fusobacterial and Low Streptococcal Signature [Article]. *SCIENTIFIC REPORTS*, *7*(1), Article 9934. <https://doi.org/10.1038/s41598-017-09786-x>

Silver, N., Hoying, D., Lamarre, E., Prendes, B., Ku, J., Dai, J., McGrail, D., Scharpf, J., Culbert, A., Campbell, S., Yilmaz, E., Geiger, J., Shah, A., Myers, J., Fredenburg, K., Woody, N., & Koyfman, S. (2023). Characterizing the intra-tumoral microbiome of laryngeal squamous cell carcinoma [Conference Abstract]. *CLINICAL CANCER RESEARCH*, *29*(18). <https://doi.org/10.1158/1557-3265.AACRAHNS23-PO-090>

Singh, S., Singh, S., Tiwari, M. B., Pal, U. S., & Kumar, S. (2019). Microflora analysis in the postchemotherapy patients of oral cancer [Article]. *National Journal of Maxillofacial Surgery*, *10*(2), 141-145. <https://doi.org/10.4103/njms.NJMS_7_19>

Singh, S., Yadav, P. K., & Singh, A. K. (2023). Structure based High-Throughput Virtual Screening, Molecular Docking and Molecular Dynamics Study of anticancer natural compounds against fimbriae (FimA) protein of Porphyromonas gingivalis in oral squamous cell carcinoma [Article]. *Molecular Diversity*. <https://doi.org/10.1007/s11030-023-10643-5>

Sobczynska-Rak, A., Zylinska, B., Polkowska, I., & Szponder, T. (2018). Elevated EGF Levels in the Blood Serum of Dogs with Periodontal Diseases and Oral Tumours. *IN VIVO*, *32*(3), 507-515. <https://doi.org/10.21873/invivo.11268>

Stashenko, P., Yost, S., Choi, Y., Danciu, T., Chen, T., Yoganathan, S., Kressirer, C., Ruiz-Tourrella, M., Das, B., Kokaras, A., & Frias-Lopez, J. (2019). The Oral Mouse Microbiome Promotes Tumorigenesis in Oral Squamous Cell Carcinoma. *MSYSTEMS*, *4*(4), Article e00323-19. <https://doi.org/10.1128/mSystems.00323-19>

Su, S. C., Chang, L. C., Huang, H. D., Peng, C. Y., Chuang, C. Y., Chen, Y. T., Lu, M. Y., Chiu, Y. W., Chen, P. Y., & Yang, S. F. (2021). Oral microbial dysbiosis and its performance in predicting oral cancer [Article]. *CARCINOGENESIS*, *42*(1), 127-135. <https://doi.org/10.1093/carcin/bgaa062>

Talamini, R., Vaccarella, S., Barbone, F., Tavani, A., La Vecchia, C., Herrero, R., Muñoz, N., & Franceschi, S. (2000). Oral hygiene, dentition, sexual habits and risk of oral cancer. *Br J Cancer*, *83*(9), 1238-1242. <https://doi.org/10.1054/bjoc.2000.1398>

Tezal, M., Grossi, S. G., & Genco, R. J. (2005). Is periodontitis associated with oral neoplasms? [Article]. *JOURNAL OF PERIODONTOLOGY*, *76*(3), 406-410. <https://doi.org/10.1902/jop.2005.76.3.406>

Ueda, S., Goto, M., Hashimoto, K., Hasegawa, S., Imazawa, M., Takahashi, M., Oh-Iwa, I., Shimozato, K., Nagao, T., & Nomoto, S. (2021). Salivary CCL20 level as a biomarker for oral squamous cell carcinoma [Article]. *Cancer Genomics and Proteomics*, *18*(2), 103-112. <https://doi.org/10.21873/CGP.20245>

Vesty, A., Gear, K., Biswas, K., Radcliff, F. J., Taylor, M. W., & Douglas, R. G. (2018). Microbial and inflammatory-based salivary biomarkers of head and neck squamous cell carcinoma [Article]. *Clinical and Experimental Dental Research*, *4*(6), 255-262. <https://doi.org/10.1002/cre2.139>

Vozza, I., Caldarazzo, V., Polimeni, A., & Ottolenghi, L. (2015). Periodontal disease and cancer patients undergoing chemotherapy [Article]. *INTERNATIONAL DENTAL JOURNAL*, *65*(1), 45-48. <https://doi.org/10.1111/idj.12133>

Wen, B. W., Tsai, C. S., Lin, C. L., Chang, Y. J., Lee, C. F., Hsu, C. H., & Kao, C. H. (2014). Cancer risk among gingivitis and periodontitis patients: A nationwide cohort study [Article]. *QJM: An International Journal of Medicine*, *107*(4), 283-290. <https://doi.org/10.1093/qjmed/hct248>

Wu, L., Yang, J., She, P., Kong, F., Mao, Z., & Wang, S. (2023). Single-cell RNA sequencing and traditional RNA sequencing reveals the role of cancer-associated fibroblasts in oral squamous cell carcinoma cohort [Article]. *FRONTIERS IN ONCOLOGY*, *13*, Article 1195520. <https://doi.org/10.3389/fonc.2023.1195520>

Xiao, T., Hu, G., Li, T., Zhu, X., Wang, H., & Zhu, Z. (2025). Causal association between periodontitis and oral cancer: a two-sample Mendelian randomization study [Article]. *Discover Oncology*, *16*(1), Article 964. <https://doi.org/10.1007/s12672-025-02528-w>

Xiong, J., Liu, H., Li, C. H., Li, Y., & Feng, J. L. (2024). Linking periodontitis with 20 cancers, emphasis on oropharyngeal cancer: a Mendelian randomization analysis. *Scientific Reports*, *14*(1), Article 12511. <https://doi.org/10.1038/s41598-024-63447-4>

Yan, K., Auger, S., Diaz, A., Naman, J., Vemulapalli, R., Hasina, R., Izumchenko, E., Shogan, B., & Agrawal, N. (2023). Microbial Changes Associated With Oral Cavity Cancer Progression [Article]. *Otolaryngology - Head and Neck Surgery (United States)*, *168*(6), 1443-1452. <https://doi.org/10.1002/ohn.211>

Yang, C. Y., Yeh, Y. M., Yu, H. Y., Chin, C. Y., Hsu, C. W., Liu, H., Huang, P. J., Hu, S. N., Liao, C. T., Chang, K. P., & Chang, Y. L. (2018). Oral Microbiota Community Dynamics Associated With Oral Squamous Cell Carcinoma Staging. *FRONTIERS IN MICROBIOLOGY*, *9*, Article 862. <https://doi.org/10.3389/fmicb.2018.00862>

Yang, J., He, P., Zhou, M., Li, S., Zhang, J., Tao, X., Wang, A., & Wu, X. (2022). Variations in oral microbiome and its predictive functions between tumorous and healthy individuals. *J Med Microbiol*, *71*(8). <https://doi.org/10.1099/jmm.0.001568>

Yang, K., Wang, Y., Zhang, S., Zhang, D., Hu, L., Zhao, T., & Zheng, H. (2021). Oral Microbiota Analysis of Tissue Pairs and Saliva Samples From Patients With Oral Squamous Cell Carcinoma – A Pilot Study [Article]. *FRONTIERS IN MICROBIOLOGY*, *12*, Article 719601. <https://doi.org/10.3389/fmicb.2021.719601>

Yang, S. F., Huang, H. D., Fan, W. L., Jong, Y. J., Chen, M. K., Huang, C. N., Chuang, C. Y., Kuo, Y. L., Chung, W. H., & Su, S. C. (2018). Compositional and functional variations of oral microbiota associated with the mutational changes in oral cancer. *ORAL ONCOLOGY*, *77*, 1-8. <https://doi.org/10.1016/j.oraloncology.2017.12.005>

Yang, Y., Li, Q., Qiao, Q., Zhao, N., Huang, H., Zhou, Y., Guo, C., & Guo, Y. (2023). Bacterial distribution and inflammatory cytokines associated with oral cancer with and without jawbone invasion—a pilot study [Article]. *CLINICAL ORAL INVESTIGATIONS*, *27*(12), 7285-7293. <https://doi.org/10.1007/s00784-023-05319-1>

Ye, P., Liu, Y., Cai, Y. J., Yang, H., Xu, H. T., & Lu, Z. Y. (2021). Microbial community alteration in tongue squamous cell carcinoma [Article]. *APPLIED MICROBIOLOGY AND BIOTECHNOLOGY*, *105*(21-22), 8457-8467. <https://doi.org/10.1007/s00253-021-11593-4>

Yost, S., Stashenko, P., Choi, Y., Kukuruzinska, M., Genco, C. A., Salama, A., Weinberg, E. O., Kramer, C. D., & Frias-Lopez, J. (2018). Increased virulence of the oral microbiome in oral squamous cell carcinoma revealed by metatranscriptome analyses [Article]. *INTERNATIONAL JOURNAL OF ORAL SCIENCE*, *10*(4), Article 32. <https://doi.org/10.1038/s41368-018-0037-7>

Zhang, L., Liu, Y., Zheng, H. J., & Zhang, C. P. (2020). The Oral Microbiota May Have Influence on Oral Cancer. *FRONTIERS IN CELLULAR AND INFECTION MICROBIOLOGY*, *9*, Article 476. <https://doi.org/10.3389/fcimb.2019.00476>

Zhang, M., Zhang, H., Hong, A., Huang, J., Yang, L., Long, Y., & Yu, Z. (2025). Dynamic changes of dental plaque and saliva microbiota in OSCC progression [Article]. *Clinical Oral Investigations*, *29*(6), Article 314. <https://doi.org/10.1007/s00784-025-06391-5>

Zhang, Z., Feng, Q., Li, M., Li, Z., Xu, Q., Pan, X., & Chen, W. (2022). Age-Related Cancer-Associated Microbiota Potentially Promotes Oral Squamous Cell Cancer Tumorigenesis by Distinct Mechanisms [Article]. *FRONTIERS IN MICROBIOLOGY*, *13*, Article 852566. <https://doi.org/10.3389/fmicb.2022.852566>

Zhao, H., Chu, M., Huang, Z., Yang, X., Ran, S., Hu, B., Zhang, C., & Liang, J. (2017). Variations in oral microbiota associated with oral cancer. *Sci Rep*, *7*(1), 11773. <https://doi.org/10.1038/s41598-017-11779-9>

Zheng, T. Z., Boyle, P., Hu, H. F., Duan, J., Jian, P. J., Ma, D. Q., Shui, L. P., Niu, S. R., Scully, C., & MacMahon, B. (1990). Dentition, oral hygiene, and risk of oral cancer: a case-control study in Beijing, People's Republic of China. *Cancer Causes Control*, *1*(3), 235-241. <https://doi.org/10.1007/bf00117475>

Zhong, X., Lu, Q., Zhang, Q., He, Y., Wei, W., & Wang, Y. (2021). Oral microbiota alteration associated with oral cancer and areca chewing [Article]. *ORAL DISEASES*, *27*(2), 226-239. <https://doi.org/10.1111/odi.13545>

Zhong, Z., Jin, Q., Zhang, J., Park, Y. M., Shrestha, D., Bai, J., & Merchant, A. T. (2020). Serum IgG Antibodies against Periodontal Microbes and Cancer Mortality [Article]. *JDR Clinical and Translational Research*, *5*(2), 166-175. <https://doi.org/10.1177/2380084419859484>

Zhou, J. H., Wang, L. L., Yuan, R. T., Yu, X. J., Chen, Z. G., Yang, F., Sun, G. R., & Dong, Q. J. (2020). Signatures of Mucosal Microbiome in Oral Squamous Cell Carcinoma Identified Using a Random Forest Model. *CANCER MANAGEMENT AND RESEARCH*, *12*, 5353-5363. <https://doi.org/10.2147/CMAR.S251021>
